# Nanangenines: drimane sesquiterpenoids as the dominant metabolite cohort of a novel Australian fungus, *Aspergillus nanangensis*

**DOI:** 10.3762/bjoc.15.256

**Published:** 2019-11-05

**Authors:** Heather J Lacey, Cameron L M Gilchrist, Andrew Crombie, John A Kalaitzis, Daniel Vuong, Peter J Rutledge, Peter Turner, John I Pitt, Ernest Lacey, Yit-Heng Chooi, Andrew M Piggott

**Affiliations:** 1 Microbial Screening Technologies, Smithfield, NSW 2164, Australia; 2 School of Chemistry, The University of Sydney, NSW 2006, Australia; 3 School of Molecular Sciences, The University of Western Australia, WA 6009, Australia; 4 Department of Molecular Sciences, Macquarie University, NSW 2109, Australia

**Keywords:** *Aspergillus*, biosynthesis, drimane, secondary metabolites, sesquiterpenoid, terpenes

## Abstract

Chemical investigation of an undescribed Australian fungus, *Aspergillus nanangensis*, led to the identification of the nanangenines – a family of seven new and three previously reported drimane sesquiterpenoids. The structures of the nanangenines were elucidated by detailed spectroscopic analysis supported by single crystal X-ray diffraction studies. The compounds were assayed for in vitro activity against bacteria, fungi, mammalian cells and plants. Bioinformatics analysis, including comparative analysis with other acyl drimenol-producing Aspergilli, led to the identification of a putative nanangenine biosynthetic gene cluster that corresponds to the proposed biosynthetic pathway for nanangenines.

## Introduction

The fungal genus *Aspergillus* is well recognised as a source of structurally diverse terpenoids comprising monoterpenoids [1], sesquiterpenoids [2–5], diterpenoids [6], sesterterpenoids [7–9], triterpenoids [10] and prenylated polyketide meroterpenoids [11–15] isolated from soil, endophytes and marine strains. Of this genus, *A. ustus* [16], *A. calidoustus* [17], *A. insuetus* [17], *A. insulicola* [18], *A. bridgeri* [18], *A. sclerotiorum* [19], *A. variecolor* [19], *A. parasiticus* [20], *A. oryzae* [21], *A. ochraceus* [22], *A. pseudodeflectus* [17], *A. carneus* [23] and *Aspergillus* sp. strain IBWF002-96 [4–5] are biosynthetic sources of the drimane sesquiterpenoids. Drimane sesquiterpenoids, which are derived from a parent C_15_ pentamethyl-*trans*-decalin skeleton, are known to occur in plants, sponges, molluscs and other fungi as well and possess a wide range of bioactivities [24]. The drimane sesquiterpenoids isolated from *Aspergillus* spp. have exhibited in vitro anti-inflammatory [5] and antiviral [22] activities as well as cytotoxicity against several mammalian cell lines [4,16,22].

Continuing our chemotaxonomic exploration of unusual Australian species of the genus *Aspergillus* [25–28], a soil survey was completed in the Kingaroy District of the South Burnett region of South East Queensland. One fungal strain, isolated from soil collected near the town of Nanango, Queensland, showed atypical growth patterns with distinct macro- and micro-morphological differences to other Aspergilli. This strain was considered to be a new species, *Aspergillus nanangensis*, belonging to the subgenus *Circumdati*, section *Jani*. The detailed morphological, genomic and chemotaxonomic characterisation of *A. nanangensis* will be reported elsewhere in due course. Herein, we report the isolation, structure elucidation and bioassay of a family of drimane sesquiterpenoids from *A. nanangensis*, which we named the nanangenines. Notably, *A. nanangensis* distinguishes itself within the genus by the production of terpenoids as the dominant biosynthetic class of secondary metabolites.

## Results and Discussion

### Purification and identification

The metabolite profile of *A. nanangensis* was examined on a limited range of solid and liquid media suitable for fungal metabolite production. The metabolite profile remained consistent despite variations to the carbon and nitrogen sources across agars and liquid media, but the productivity was superior on grains, notably rice and barley. A search of the UV–vis profiles against our in-house library of type species (>25,000 spectra from 2,000 species, including 205 *Aspergillus* type species) and unidentified but metabolically talented fungi (>60,000 spectra from 3,000 species) returned no similar metabolite cohorts, suggesting an unknown species. Individual retention time/UV–vis searches of the dominant 15 secondary metabolites against our in-house pure metabolite library (>7,100 standards) also failed to provide a single known secondary metabolite, further suggesting the strain was a hitherto unaccounted species of *Aspergillus*.

**Figure 1:**
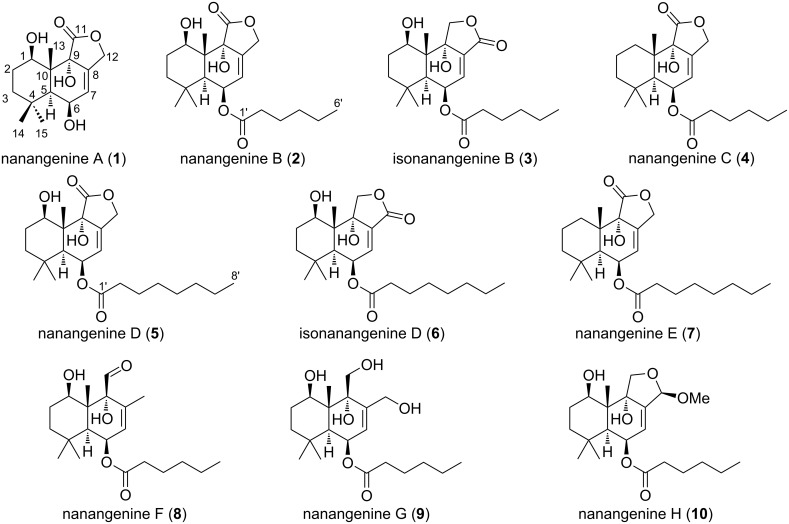
Structures of nanangenines **1**–**10** isolated from *A. nanangensis*.

*A. nanangensis* was cultivated separately on jasmine rice and pearl barley for 21 days, which resulted in confluent and thick mycelial coverage of the grains. Extraction of the grains with acetone, followed by partitioning of the aqueous residue with EtOAc and defatting with hexane, provided an enriched extract of non-polar secondary metabolites. Fractionation by reversed-phase preparative HPLC (Figures S1 and S2 in Supporting Information File 1) yielded ten drimane metabolites shown in Figure 1: one trihydroxylated drimane sesquiterpenoid lactone, nanangenine A (**1**), four drimane lactones bearing C_6_/C_8_ acyl chains, nanangenines B, C, D and E (**2**, **4**, **5** and **6**), two acylated drimanes bearing isomeric lactones, isonanangenines B and D (**3** and **7**), and three acylated drimanes, nanangenines F–H (**8**–**10**), which are putative biosynthetic intermediates. The structures of **1**–**10** were elucidated by detailed spectroscopic analysis, while absolute configurations were determined by single crystal X-ray diffraction analysis of selected analogues.

High-resolution positive electrospray ionisation mass spectrometry (HRESI(+)MS) analysis of nanangenine A (**1**) revealed an adduct ion ([M + Na]^+^ *m*/*z* 305.1363) indicative of a molecular formula C_15_H_22_O_5_ requiring five double bond equivalents (DBE). No distinguishing absorption maxima were observed in the UV–vis spectrum, while absorptions at 3354 and 1738 cm^−1^ in the IR spectrum were indicative of hydroxy and carbonyl groups, respectively. The ^13^C NMR data for **1** (Table 1) indicated the presence of one carbonyl carbon (δ_C_ 179.3, C-11) and two olefinic carbons (δ_C_ 128.2, C-7 and δ_C_ 131.1, C-8), accounting for two DBE and thus requiring **1** to be tricyclic. The ^1^H and ^13^C NMR data also revealed the presence of one hydroxylated quaternary carbon (C-9), two aliphatic quaternary carbons (C-4, C-10), two oxymethines (C-1, C-6), one aliphatic methine (C-5), one oxymethylene (C-12), two aliphatic methylenes (C-2, C-3), three methyl groups (C-13, C-14, C-15) and three hydroxy groups (1-OH, 6-OH, 9-OH). Detailed analysis of the 2D NMR data for **1** (Table S3 in Supporting Information File 1) confirmed the presence of a drimane sesquiterpenoid lactone scaffold. A search of the literature revealed **1** to be almost identical to strobilactone B, previously reported from *A. ustus* [29], with the only difference being hydroxylation at C-1 in **1**, instead of at C-2. Therefore, the structure of **1** was assigned as shown in Figure 1. The absolute configuration of **1** was confirmed to be 1*R,*5*S,*6*R,*9*R,*13*R* by single crystal X-ray diffraction analysis (Table S2 and Figure S3 in Supporting Information File 1).

**Table 1:** NMR data for nanangenine A (**1**) in DMSO-*d*_6_.

Position	δ_C_^a^	δ_H_, mult. (*J* in Hz)^b^

1	69.0	4.12, dd (8.4, 7.8)
2	26.2	1.54, m
3	41.5	1.22, m
4	33.6	
5	45.0	1.52, d (5.0)
6	63.3	4.30, m
7	128.2	5.92, m
8	131.1	
9	76.1	
10	42.4	
11	179.3	
12a	70.6	5.00, ddd (12.2, 2.5, 2.4)
12b		4.91, ddd (12.2, 1.3, 1.2)
13	12.6	0.92, s
14	24.3	1.23, s
15	31.9	1.02, s
1-OH		4.76, s
6-OH		4.81, br d (5.8)
9-OH		6.50, s

^a^Acquired at 125 MHz; ^b^acquired at 500 MHz.

HRESI(+)MS analysis of nanangenine B (**2**) revealed an adduct ion ([M + Na]^+^ *m*/*z* 403.2096) indicative of a molecular formula C_21_H_32_O_6_. The NMR data for **2** (Table S4 in Supporting Information File 1) were very similar to those for **1**, with the only significant differences being the absence of the 6-OH proton, the presence of additional signals for a C_6_ acyl chain, and significant deshielding of H-6 from δ_H_ 4.30 to 5.47 ppm. Therefore, the structure of **2** was assigned as the 6-*O*-hexanoyl analogue of **1**, as shown in Figure 1. Compound **2** was previously reported in 2014 as an unnamed metabolite from *Aspergillus* sp. IBWF002-96 [5], and we have assigned the trivial name nanangenine B for consistency. The absolute configuration of **2** was assigned based on single crystal X-ray diffraction analysis of a 9-*O*-(4-bromobenzoyl) derivative (**2b**; Table S2 and Figure S4 in Supporting Information File 1).

HRESI(+)MS analysis of isonanangenine B (**3**) revealed an adduct ion ([M + Na]^+^ *m*/*z* 403.2094) indicative of a molecular formula C_21_H_32_O_6_, which is isomeric with **2**. Comparison of the NMR data for **3** (Table S5 in Supporting Information File 1) with those for **2** revealed the only difference to be the position of the lactone carbonyl group, which was determined to be at C-12 instead of C-11 based on key HBMC correlations from H-7 to C-12 and 9-OH to C-11. Therefore, the structure of **3** was assigned as shown in Figure 1. Compound **3** was previously reported in 2013 as an unnamed metabolite (code number SF002-96-1) from *Aspergillus* sp. IBWF002-96 [4], and we have assigned the trivial name isonanangenine B for consistency. The absolute configuration of **3** was assigned based on single crystal X-ray diffraction analysis of a 1-*O*-(4-bromobenzoyl) derivative (**3b**; Table S2 and Figure S5 in Supporting Information File 1).

HRESI(+)MS analysis of nanangenine C (**4**) revealed an adduct ion ([M + Na]^+^ *m*/*z* 387.2147) indicative of a molecular formula C_21_H_32_O_5_, which has one fewer oxygen atom than **2** and **3**. Comparison of the NMR data for **4** (Table S6 in Supporting Information File 1) with those for **2** revealed the only significant differences to be the absence of the H-1 and 1-OH protons and the presence of an additional methylene group (H_2_-1). Therefore, the structure of **4** was assigned to be the 1-deoxy analogue of **2**, as shown in Figure 1. Compound **4** was previously reported in 2014 as an unnamed metabolite from *Aspergillus* sp. IBWF002-96 [5], and we have assigned the trivial name nanangenine C for consistency. The absolute configuration of **4** was assigned based on single crystal X-ray diffraction analysis (Table S2 and Figure S6 in Supporting Information File 1).

Nanangenine D (**5**), isonanangenine D (**6**) and nanangenine E (**7**) had virtually identical spectroscopic data to **2**, **3** and **4**, respectively, with the only significant differences being the presence of two additional resonances in the aliphatic region of the ^13^C NMR spectra (Table 2) and an increase in area of four protons in the methylene envelopes of the ^1^H NMR spectra. Therefore, the structures of **5**–**7** were assigned to be the C_8_ homologs of **2**–**4**, respectively, as shown in Figure 1. The absolute configurations of **5**–**7** were assigned to be the same as **2**–**4** based on their similar NMR data and optical rotations.

**Table 2:** NMR data for nanangenine D (**5**), isonanangenine D (**6**) and nanangenine E (**7**) in DMSO-*d*_6_.

Position	Nanangenine D (**5**)		Isonanangenine D (**6**)		Nanangenine E (**7**)	
	δ_C_^a^	δ_H_, mult. (*J* in Hz)^b^	δ_C_^a^	δ_H_, mult. (*J* in Hz)^b^	δ_C_^a^	δ_H_, mult. (*J* in Hz)^b^

1a	68.7	4.17, ddd (10.9, 5.9, 0.9)	68.6	3.95, ddd (12.0, 5.9, 3.9)	29.4	1.95, ddd (15.0, 13.5, 4.3)
1b						1.81, dm (13.5)
2a	25.9	1.57, m	27.2	1.53, m	17.4	1.59, m
2b						1.47, m
3a	41.3	1.28, m	41.7	1.31, ddd (13.3, 3.5, 3.5)	44.3	1.33, dm (13.0)
3b				1.23, m		1.19, m
4	33.1		32.9		33.3	
5	43.8	1.87, d (4.9)	44.1	1.99, d (5.2)	44.0	1.97, d (4.9)
6	66.0	5.46, m	66.0	5.59, dd (5.1, 3.9)	66.0	5.47, m
7	121.9	5.88, m	131.6	6.45, d (3.9)	121.3	5.77, m
8	135.5		133.2		136.5	
9	75.6		75.0		73.1	
10	42.4		43.2		37.2	
11a	178.4		76.4	4.41, dd (10.2, 0.5)	174.3	
11b				4.19, br d (10.2)		
12a	70.3	5.03, ddd (12.8, 4.8, 2.5)	168.6		68.2	4.87, ddd (12.8, 2.5, 2.5)
12b		4.94, ddd (12.8, 1.7, 1.2)				4.74, ddd (12.8, 1.2, 1.2)
13	12.2	0.94, s	12.3	0.94, s	18.1	0.99, s
14	24.0	1.06, s	24.2	1.08, s	24.2	1.08, s
15	31.5	0.90, s	31.9	0.91, s	32.1	0.91, s
1'	172.2		172.2		172.2	
2'a	34.0	2.33, m	33.9	2.36, m	34.0	2.32, m
2'b		2.27, m		2.27, m		2.25, m
3'	24.2	1.53, m	24.2	1.53, m	24.2	1.52, m
4'	28.2	1.24, m	28.2	1.23, m	28.3	1.24, m
5'	28.3	1.24, m	28.2	1.23, m	28.2	1.24, m
6'	31.0	1.22, m	31.0	1.21, m	31.0	1.21, m
7'	21.9	1.24, m	21.9	1.23, m	21.9	1.24, m
8'	13.9	0.84, t (7.9)	13.9	0.83, t (7.2)	13.9	0.84, t (7.2)
1-OH		4.71, d (0.9)		4.62, d (5.1)		–
9-OH		6.77 s		5.61 br s		6.24 s

^a^Acquired at 150 MHz; ^b^acquired at 600 MHz.

HRESI(+)MS analysis of nanangenine F (**8**) revealed a protonated molecule ([M + H]^+^ *m*/*z* 367.2483) indicative of a molecular formula C_21_H_34_O_5_, requiring one fewer DBE than **2**. Indeed, the NMR data for **8** (Table 3) were very similar to those for **2**, with the main differences being the absence of signals for the lactone carbonyl and oxymethylene group, and the presence of signals for putative aldehyde (δ_C_ 203.3; δ_H_ 9.49, s) and olefinic methyl (δ_C_ 19.5; δ_H_ 1.51, dd) groups. Key HMBC correlations (Table S10 in Supporting Information File 1) from 9-OH to C-11 and from H-11 to C-9 positioned the aldehyde on C-9, while HMBC correlations from H-7 to C-12 and from H_3_-12 to C-9 positioned the methyl on C-8. Therefore, the structure of **8** was assigned as the seco analogue of **2**, as shown in Figure 1. The absolute configuration of **8** was assigned to be the same as **2** based on their similar NMR data and optical rotations, supported by key ROESY correlations between H_3_-13 and H-11, and between H-5 and 9-OH (Table S10 in Supporting Information File 1).

**Table 3:** NMR data for nanangenines F-H (**8**–**10**) in DMSO-*d*_6_.

Position	Nanangenine F (**8**)		Nanangenine G (**9**)		Nanangenine H (**10**)	
	δ_C_^a^	δ_H_, mult. (*J* in Hz)^b^	δ_C_^c^	δ_H_, mult. (*J* in Hz)^d^	δ_C_^a^	δ_H_, mult. (*J* in Hz)^b^

1	68.5	3.88, ddd (11.9, 5.8, 5.8)	69.1	3.93 dd (11.2, 4.3)	68.4	3.93, ddd (11.8, 5.4, 5.0)
2a	27.3	1.51, m	28.3	1.56, m	27.5	1.53, m
2b		1.47, m				1.48, m
3a	41.6	1.26, m	41.6	1.23, m	41.9	1.27, m
3b		1.21, m				1.22, m
4	32.8		33.5		32.8	
5	43.5	1.84, d (4.7)	44.9	1.83, d (4.4)	44.3	1.95, d (4.0)
6	66.5	5.37, m	67.0	5.41, m	67.1	5.46, ddd (5.0, 3.9, 1.4)
7	124.0	5.53, dq (5.1, 1.5)	121.4	5.78, br d (5.3)	120.3	5.57, dd (3.9,1.4)
8	137.2		144.1		145.0	
9	80.1		75.6		78.3	
10	47.0		45.9		43.5	
11a	203.3	9.49, br s	62.2	3.73, m	74.5	3.84, d (9.7)
11b				3.67, m		3.76, d (9.7)
12a	19.5	1.51, dd, (1.3, 1.3)	61.0	4.05, m	101.9	5.31, t (1.4)
12b				4.02, m		
13	11.5	1.06, s	12.5	1.06, s	11.7	0.92, s
14	24.3	1.05, s	24.4	1.03, s	24.3	1.08, s
15	31.8	0.89, s	32.4	0.87, br s	32.1	0.89, s
1'	172.3		172.7		172.3	
2'a	34.1	2.30, m	34.5	2.24, m	34.1	2.32, m
2'b		2.23, m		2.21, m		2.23, m
3'	23.9	1.53, m	24.2	1.53, m	24.0	1.52, m
4'	30.6	1.25, m	30.9	1.25, m	30.5	1.24, m
5'	21.7	1.26, m	22.0	1.26, m	21.7	1.25, m
6'	13.7	0.84, t (7.0)	13.9	0.83, t (7.1)	13.9	0.84, t (7.1)
12-OMe	–	–	–	–	54.1	3.26, s
1-OH		4.74, d, (5.7)		5.07, br s		4.37, d (5.4)
9-OH		5.46, s		4.41, br s		4.93, s
11-OH		–		5.27, br s		–
12-OH		–		4.88, br s		–

^a^Acquired at 150 MHz; ^b^acquired at 600 MHz; ^c^acquired at 125 MHz; ^d^acquired at 500 MHz.

HRESI(+)MS analysis of nanangenine G (**9**) revealed an adduct ion ([M + Na]^+^ *m/z* 407.2406) indicative of a molecular formula C_21_H_36_O_6_. The NMR data for **9** (Table 3) were very similar to those for **2**, with the main differences being the absence of a signal for the lactone carbonyl group and the presence of additional signals for an additional oxymethylene (δ_C_ 62.2; δ_H_ 3.73/3.67, m) and two additional hydroxy groups (δ_H_ 5.27, br s and 4.88, br s). The ^1^H NMR signals for oxymethylene H_2_-12 were also shifted upfield by ≈1 ppm compared to those in **2**, suggesting the pendant oxygen atom was no longer attached to a carbonyl carbon. A detailed analysis of the 2D NMR data for **9** (Table S11 in Supporting Information File 1) confirmed the structure to be the seco analogue of **2**, as shown in Figure 1. The absolute configuration of **9** was then confirmed to be the same as **2** by single crystal X-ray diffraction analysis (Table S2 and Figure S7 in Supporting Information File 1).

HRESI(+)MS analysis of nanangenine H (**10**) revealed a protonated dehydration product ([M – H_2_O + H]^+^ *m*/*z* 379.2473) indicative of a molecular formula C_22_H_36_O_6_. The NMR data for **10** (Table 3) were very similar to those for **3**, with the main differences being the absence of a signal for the lactone carbonyl group and the presence of additional signals for acetal (δ_C_ 101.9; δ_H_ 5.31, t) and methoxy (δ_C_ 54.1; δ_H_ 3.26, s) groups. Key HMBC correlations (Table S12 in Supporting Information File 1) from H-7 to C-12 and from 12-OMe to C-12 positioned the methoxy group and acetal proton on C-12. Thus, the structure of **10** was assigned as shown in Figure 1. The configuration of **10** at C-12 was determined to be 12*R* based on a key ROESY correlation between H-12 and 9-OH (Table S12, Supporting Information File 1), with the absolute configuration of the remainder of the molecule assumed to be the same as **3** based on their similar NMR data and optical rotations.

### Bioassays

The nanangenines were assayed for in vitro activity against four mammalian cell lines, two bacteria, one fungus and one plant (Table 4). Compound **1** was inactive up to 100 μg mL^−1^ in all of the assays performed, suggesting acylation at 6-OH is important for biological activity. Compounds **4**, **5** and **7** showed moderate antibacterial activity against *B. subtilis*, with weaker activity observed for **2**, **3**, **6** and **8**. Compounds **2**, **5**, **8**, **9** and **10** exhibited low levels of cytotoxicity against the four mammalian cell lines tested, while **3**, **6** and **7** were considerably more cytotoxic. Notably, **4** showed strong activity against the mouse myeloma NS-1 cell line, but no activity against the three human cell lines. None of the compounds tested showed any activity up to 100 μg mL^−1^ against the Gram-negative bacterium *Escherichia coli* (ATCC 25922), the fungus *Candida albicans* (ATCC 10231) or the plant *Eragrostis tef* (teff).

**Table 4:** In vitro bioassay results for nanangenines **1**–**10**.

Compound	IC_50_ (μg mL^−1^)^a^				
	Bs^b^	NS-1^c^	DU-145^d^	MCF-7^e^	Nff^f^

**1**	>100	>100	>100	>100	>100
**2**	62 ± 2	38 ± 1.5	>100	>100	>100
**3**	56 ± 6	0.16 ± 0.01	0.9 ± 0.2	0.19 ± 0.05	0.9 ± 0.1
**4**	5.7 ± 0.1	4.1 ± 0.1	>100	>100	>100
**5**	9.4 ± 0.4	19 ± 2	37 ± 2	22 ± 1	37 ± 2
**6**	4%^g^	1.0 ± 0.1	32 ± 3	3.6 ± 0.4	2.1 ± 0.3
**7**	2.3 ± 0.3	2.4 ± 0.1	15 ± 1	9.4 ± 0.7	6.8 ± 0.6
**8**	78 ± 2	49 ± 2	95 ± 3	49 ± 1	84 ± 2
**9**	>100	47 ± 2	>100	>100	>100
**10**	>100	10 ± 2	7%^g^	62 ± 2	60 ± 1
control	0.4^h^	0.02^i^	18^i^	2^i^	0.2^i^

^a^Experiments were conducted in triplicate. IC_50_ values are mean ± standard error; ^b^*Bacillus subtilis* (ATCC 6633); ^c^mouse myeloma NS-1 cell line (ATCC TIB-18); ^d^human prostate cancer DU-145 cell line (ATCC HTB-81); ^e^Human breast adenocarcinoma MCF-7 cell line (ATCC HTB-22); ^f^Human fibroblast NFF cell line (ATCC PCS-201); ^g^incomplete dose response, % inhibition reached at 100 μg mL^−1^; ^h^tetracycline; ^i^staurosporine.

### Proposed biosynthesis and gene cluster

The biosynthesis of drimane-type sesquiterpenoids from farnesyl diphosphate is proposed to proceed via the protonation-initiated mechanism (class II terpene synthases) [24], which is distinct from the ionisation-initiated mechanism (class I) terpene synthases, where a carbocation is generated by the release of a diphosphate group [30]. Therefore, the drimane synthase is likely to be different from the commonly observed sesquiterpene synthase, which belongs to the class I terpene synthases. Recently, the drimane synthase AstC involved in biosynthesis of the astellolides was identified and shown to be a novel member of the terpene synthase family, showing similarity to haloacid dehalogenase (HAD)-like hydrolases [21]. Thus, we suspected that a related enzyme may be involved in the biosynthesis of the nanangenines, and used the amino acid sequence of AstC to probe the *A. nanangensis* genome. We also hypothesised that the acyl side chains present in the (iso)nanangenines could be derived either from a fatty acid synthase (FAS) or polyketide synthase (PKS). For example, in aflatoxin biosynthesis, the hexanoyl started unit is supplied by a FAS [31], while in the meroterpenoid fumagillin biosynthesis, the unsaturated acyl chain is synthesised by a PKS [32].

The *A. nanangensis* genome was sequenced and a draft assembly of the genome was generated. A local BLASTp search of the *A. nanangensis* genome using the drimane synthase AstC as query returned a hit on scaffold 3, FE257_006542, which was immediately flanked by a highly-reducing PKS (FE257_006541) and a FAD-binding oxidoreductase (FE257_006543). AstC, though annotated as a HAD-like hydrolase and having low sequence homology to other characterised terpene synthases, contains sequence motifs conserved across both class I (DDxxD/E) and class II (DxDD, QW) terpene synthases [33–35]. Specifically, AstC contains a DxDTT motif [21], which is a variant of the DxDD motif known to be involved in class II-type protonation-initiated terpene cyclisation [36]. Importantly, the DDxxD motif in AstC contains a substitution of the second Asp for Asn, leading to a loss of ionisation-activated cyclisation activity. Two other HAD-like hydrolases, AstI and AstK, with the class I (DDxxD/E) motif, are therefore required to catalyse other dephosphorylation steps. Despite overall low sequence homology, alignment with the amino acid sequences of AstC, AstI, AstK and related homologs confirmed the presence of both conserved class I and class II motifs in FE257_006542 (Figure S50 and Table S15 in Supporting Information File 1).

Drimane sesquiterpenoids are produced by several species across the *Aspergillus* genus, with compounds possessing acyl side chains observed specifically in section *Usti*, such as *A. ustus* [37–38], *A. insuetus* [39], *A. pseudodeflectus* [40] and other unnamed species [17]. *A. calidoustus*, another member of section *Usti*, also produces drimane sesquiterpenoids, though no compounds with acyl side chains have yet been reported [41]. Notably, the acyl chains that these compounds possess are unsaturated polyenes, while the acyl chains of the nanangenines are fully saturated. As noted earlier, **2**–**4** were previously reported as unnamed metabolites from *Aspergillus* sp. IBWF002-96 [4–5], which has an internal transcribed spacer (ITS) sequence that shares 100% identity with an uncultured soil sample (NCBI accession GQ921753.1) and 97% identity with *A. janus* (NCBI accession EU021598). The ITS sequence of *A. nanangensis* (deposited on NCBI, accession MK979278) also shares 100% sequence identity with the uncultured soil sample, and 97% identity with *A. janus* (Figure S51 in Supporting Information File 1). No public sequence could be found for IBWF002-96. Interestingly, the uncultured soil sample was also isolated from soil near the town of Nanango in Queensland, Australia, as part of a survey of soil fungal communities in monoculture *Araucaria cunninghamii* plantations, though it remained unidentified [42]. While we cannot conclusively determine each strain to be *A. nanangensis*, the ITS sequences of all three strains are identical and they clearly clade in section *Jani* (Figure S48 in Supporting Information File 1).

**Figure 2:**
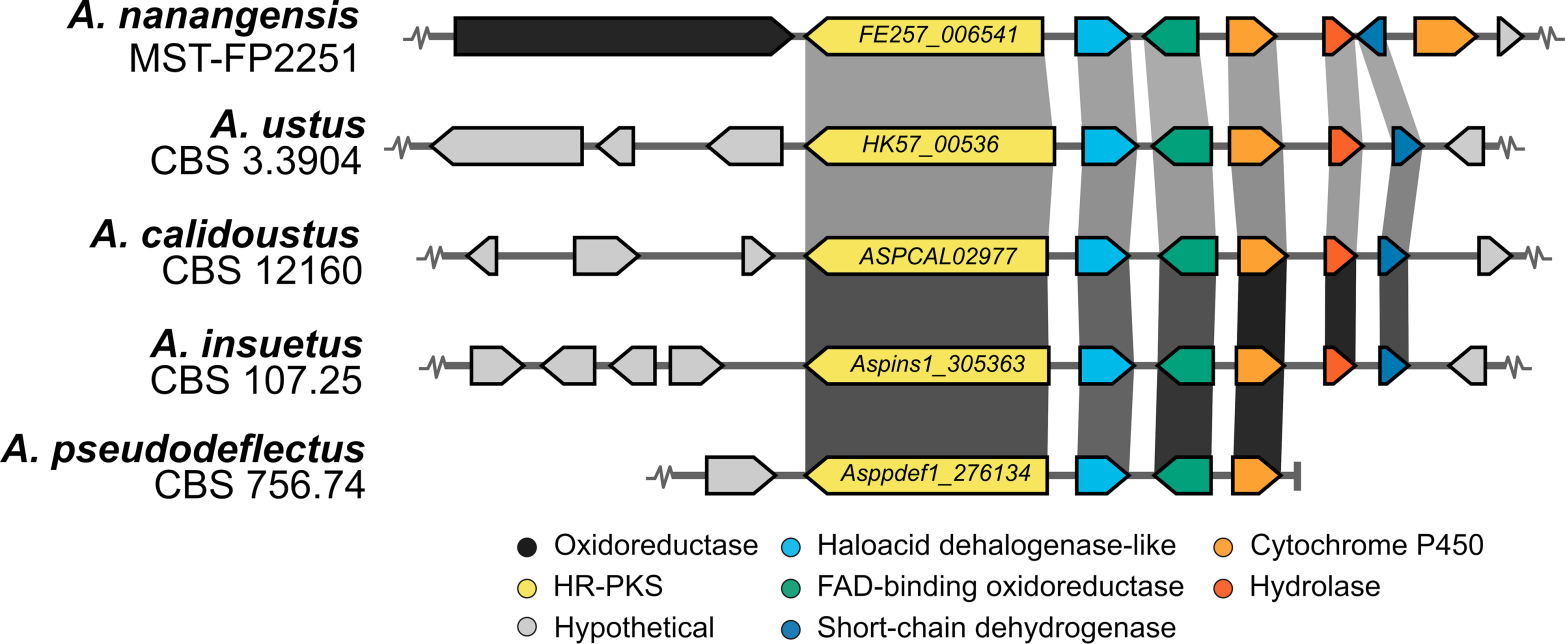
Putative nanangenine biosynthetic gene cluster in *A. nanangensis* MST-FP2251 and homologs identified in other drimane sesquiterpenoid-producing species of *Aspergillus* section *Usti*. Gene models are drawn to scale; shaded boxes that link gene models represent amino acid identity (0% transparent; 100% black).

A comparative genomics survey of all publicly available genomes on NCBI and the Joint Genome Institute (JGI) genome portal of known drimane sesquiterpenoid producers was undertaken (Figure S49 in Supporting Information File 1). Homologous gene clusters were observed in all drimane sesquiterpenoid-producing members of section *Usti*: *A. ustus* CBS 3.3904, *A. calidoustus* CBS 12160 and *A. insuetus* CBS 107.25 (Figure 2). A homologous cluster was also found in *A. pseudodeflectus* CBS 756.74, though it is located on a truncated assembly scaffold. In total, six genes are conserved across these species: the HR-PKS (FE257_006541), HAD-like terpene synthase (FE257_006542) and FAD-binding oxidoreductase (FE257_006543), as well as a cytochrome P450 (FE257_006544), an alpha/beta hydrolase (FE257_006545) and a short-chain dehydrogenase (FE257_006546). Notably, no homologous clusters were identified in other members of section *Jani* (*A. brevijanus* and *A. janus*) that have not been reported to produce such drimane sesquiterpenoids. A phylogenetic tree of all AstC homologs identified in the genomes of all known drimane sesquiterpenoid producers was constructed, in which the putative HAD-like terpene synthases (drimane synthases) from the section *Usti* form a distinct clade, separate from the AstC type (Figure S52 in Supporting Information File 1). The analysis above, and the discovery of other homologous gene clusters in *Aspergillus* spp. that are known to produce acyl drimane sesquiterpenoids, support the identification of this as the putative biosynthetic gene cluster for nanangenines.

**Figure 3:**
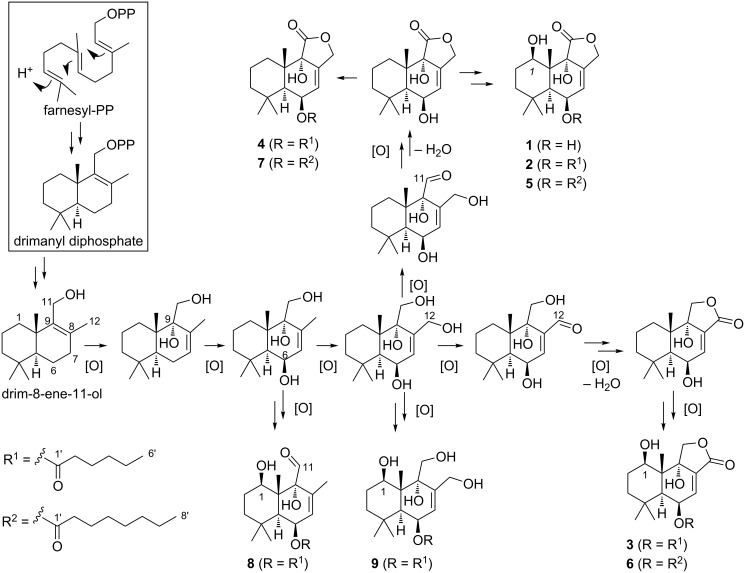
Putative biosynthetic pathway to the nanangenines.

Based on the analyses above, a biosynthetic pathway to the nanangenines was proposed (Figure 3). Unlike the *ast* cluster, where there are multiple HAD-like enzymes encoded (one terpene synthase and two phosphatases), the putative nanangenine cluster only encodes one such enzyme, FE257_006542. However, given that FE257_006542 contains both class I (DDxxD/E) and class II (DxDD, QW) terpene synthases, we propose that FE257_006542 is responsible for both the cyclisation into drimanyl diphosphate and the hydrolysis of the diphosphate into drim-8-ene-11-ol. We propose the next step in the pathway is hydroxylation at C-6 or C-9, as hydroxylation at both sites is common to all of the (iso)nanangenines. The 9-hydroxylation also results in migration of the double bond on the decalin to Δ^7,8^. The two hydroxylations could be catalysed by the FAD-dependent oxidoreductase or one of the cytochrome P450 oxygenases. From this point, the pathway branches out to the different (iso)nananagenines via combinations of hydroxylation at C-1 (or not) and one or multiple oxidations at C-11 and C-12. The oxidations of methyl to alcohol and alcohol to aldehyde could be catalysed by one of the two P450 oxygenases, or the short-chain dehydrogenase could oxidise the alcohol (at C-11 or C-12) to an aldehyde. Depending on whether C-11 or C-12 formed an aldehyde, further oxidation of the aldehyde to a carboxylic acid and condensation with the γ-OH group would afford the butyrolactone ring in nanagenines A–E and isonanagenines B/D, respectively. The C-6 and C-8 lipid chain is likely produced by the HR-PKS encoded by gene FE257_006541, while the acylation could be attributed to the enzyme encoded by FE257_006545, which contains the conserved domain of the alpha/beta-hydrolase fold superfamily (which includes thioesterases and acyltransferases, e.g., LovD [43]). Therefore, the identified putative gene cluster encodes all the genes predicted to be required for biosynthesis of the (iso)nanangenines. We are currently in the process of verifying the putative biosynthetic gene cluster by genetic deletion and heterologous expression.

## Conclusion

Our investigations into unique Australian microbial biodiversity have led to discovery of a family of drimane sesquiterpenoids, the nanangenines, which serve as key chemotaxonomic markers for the novel fungal species, *A. nanangensis*. Although related drimane sesquiterpenoid lactones have been found in several Aspergilli, drimane sesquiterpenoids with saturated fatty acyl chains have only been reported in one *Aspergillus* strain (IBWF002-96), which appears to share an identical ITS sequence with *A. nanangensis* [4–5]. Interestingly, drimane sesquiterpenoids are not produced uniformly within the *Aspergillus* genus, instead clustering within the sections *Usti*, *Circumdati* and *Flavi* (Figure S49 in Supporting Information File 1). The drimane sesquiterpenoids are not among the dominant metabolite classes commonly used for *Aspergillus* chemotaxonomy, but have been used for chemotyping of Aspergilli in the section *Usti* [17]. Here, we showed for the first time that an *Aspergillus* species from the section *Jani* produces such drimane sesquiterpenoids and has a secondary metabolite repertoire that is distinct from close members in the section *Jani*. Comparative analysis identified a group of putative homologous acyl drimane sesquiterpenoid biosynthetic gene clusters that are shared among *A. nanangensis* and Aspergilli in the section *Usti*. Interestingly, the putative nanangenine biosynthetic gene cluster, and those homologs in *Aspergillus* section *Usti*, only shared low homology to the astellolide biosynthetic gene cluster identified in *A. oryzae*. Further investigation by genetic knockout or heterologous expression is required to confirm the identity of the putative nanangenine biosynthetic gene cluster. Over the range of biological assays performed, isonanangenines **3** and **6**, in particular, exhibited strong activity against human and murine tumour cell lines. This suggests that the regioisomeric lactone moiety could play an important role in the observed cytotoxicity. Interestingly, **3** (SF002-96-1 [4]) was previously shown to inhibit survivin, which is a member of the inhibitor of apoptosis (IAP) family and a potential cancer target. Further structure–activity relationship studies are required to fully characterise this potential anticancer pharmacophore. This study again demonstrates how taxonomy-guided exploration of fungi is a highly effective strategy for identifying novel bioactive metabolites.

## Experimental

### General experimental details

Optical rotations were acquired in MeOH on a Perkin-Elmer Model 341 polarimeter in a 50 × 5 mm cell or on a Jasco P-1010 polarimeter in a 100 × 3.5 mm cell. UV–vis spectra were acquired in MeCN on a Varian Cary 4000 spectrophotometer or a Jasco V-760 spectrophotometer in a 10 × 10 mm quartz cuvette. Analytical HPLC was performed on a gradient Shimadzu VP HPLC system equipped with a Shimadzu SPD-M10A VP diode array detector and an LC-10AT VP gradient chromatograph. Preparative HPLC was performed on a gradient Shimadzu HPLC system comprising two LC-8A preparative liquid pumps with static mixer, SPD-M10AVP diode array detector and SCL-10AVP system controller with standard Rheodyne injection port. The columns used in the purification of the metabolites were selected from either a Hypersil C_18_ column (50 × 100 mm, 5 μm; Grace Discovery), a Vydac C_18_ column (50 × 100 mm, 5 μm; Grace Discovery), a Zorbax SB-C_18_ column (50 × 150 mm, 5 μm; Agilent) or an Alltima C_18_ column (22 × 250 mm, 5 μm, Grace Discovery), eluted isocratically with acetonitrile/water mixtures with or without 0.01% TFA modifier, as described for each separation. LCMS was performed on an Agilent 1260 Infinity series HPLC equipped with an Agilent 6130 Infinity series single quadrupole mass detector in both positive and negative ion modes. High-resolution electrospray ionisation mass spectra (HRESIMS) were obtained on a Bruker Apex Qe 7T Fourier Transform Ion Cyclotron Resonance mass spectrometer equipped with an Apollo II ESI/MALDI Dual source or a Q Exactive Plus hybrid quadrupole-Orbitrap mass spectrometer (Thermo Fisher Scientific, Bremen, Germany) by direct infusion. NMR data were recorded in DMSO-*d*_6_ on either a Bruker Avance III 500 or a Bruker Avance II DRX-600K spectrometer. All NMR spectra were recorded at 25 °C, processed using Bruker Topspin 3.5 software and referenced to residual non-deuterated solvent signals (DMSO-*d*_6_: δ_H_ 2.49, δ_C_ 39.5 ppm).

### Collection and cultivation

*A. nanangensis* MST-FP2251 was isolated by serial dilution of an aqueous suspension of soil collected in Nanango, Queensland, Australia in 2004. Cultures were freeze-dried and accessioned into the CSIRO Agriculture and Food (FRR) culture collection, North Ryde, NSW, Australia, with the code FRR6048. The growth of *A. nanangensis* was optimised on four different agar and liquid media and four types of grains. Agar and liquid versions of glycerol casein (AC), Czapek-Dox (CZ), malt extract (MA) and yeast extract (YS) media, and the grains, pearl barley (PB), cracked wheat (BL), jasmine rice (JR) and basmati rice (BS) were prepared in accordance with recipes provided in Table S1 in Supporting Information File 1. The culture was recovered from storage at −80 °C onto three malt agar plates. At day 7 the recovery plates showed good confluent growth and no signs of contamination. Agar plates were sliced and deposited into three Erlenmeyer flasks containing sterilised Milli-Q water (100 mL). A spore suspension was created by shaking for 1 h prior to inoculation. The agar plates were inoculated with spore suspension (500 μL), liquid media (50 mL) were inoculated with spore suspension (2 mL), and grains were inoculated with spore suspension (10 mL). The grain, agar and liquid media were placed in a dark room at 24 °C, with the liquid media flasks on a shaker pad rotating at 90 rpm. The grains and agar cultivars were sub-sampled on days 7, 14 and 21, while the liquids were sub-sampled on day 7. The sub-samples were extracted in MeOH for 2 h prior to being analysed by analytical HPLC (C_18_) to determine the secondary metabolite profile.

### Preparative cultivation, extraction and isolation

Two preparative-scale cultivations were carried out to produce sufficient quantities of the nanangenine family for characterisation and bioassay. Cultivation conditions were identical with the exception of growth medium. Culture A was prepared with jasmine rice (1.5 kg) and Culture B with pearl barley (1.5 kg). Each grain was hydrated during sterilisation (1 L water; 120 °C for 40 min). A spore suspension of a 7-day-old Petri plate of *A. nanangensis* was used to inoculate 42 × 250 mL Erlenmeyer flasks each containing 75 g of sterile medium. The flasks were incubated at 24 °C for 21 days, by which time the cultures had grown extensively throughout the grain and reached maximal metabolite productivity. Cultures were extracted separately using the same process (see Figures S1 and S2 in Supporting Information File 1). Grains were pooled from individual flasks, and extracted with acetone (2 × 3 L). The combined extracts were evaporated under vacuum to produce an aqueous slurry (500 mL). The slurry was partitioned against ethyl acetate (2 × 2 L) and the ethyl acetate was reduced in vacuo to an oily residue. The residue was dissolved in methanol (300 mL) and defatted using hexanes (2 × 500 mL) to give the crude extract.

Initial fractionation of culture A (13.5 g) was achieved using Sephadex LH-20 size-exclusion chromatography in methanol (25 × 600 mm). The crude extract was loaded onto the column and eluted with methanol at a flow rate of 4 mL min^−1^. The fractions (100 mL) were sub-sampled and analysed by C_18_ analytical HPLC. Fractions 3 and 4 were pooled and evaporated to give a solid residue (3.7 g), as were fractions 5–7 (7.8 g). Fraction 3–4 was dissolved in MeOH (8 mL) and insoluble material was pelleted by centrifugation (4000 rpm for 7 min). The supernatant was fractionated by preparative HPLC (Hypersil C_18_, isocratic 70% MeCN/H_2_O, 60 mL min^−1^) to give an enriched fraction (302.4 mg), which was further purified by preparative HPLC (Hypersil C_18_, isocratic 55% MeCN/H_2_O, 60 mL min^−1^) to yield nanangenine B (**2**; *t*_R_ = 41.0 min; 92.1 mg). Fraction 5–7 was dissolved in MeOH (9 mL) and insoluble material was pelleted by centrifugation (4000 rpm for 7 min). The supernatant was fractionated by preparative HPLC (Hypersil C_18_, isocratic 45% MeCN/H_2_O containing 0.01% TFA, 60 mL min^−1^) which yielded four subfractions (1, 3, 4 and 7) regarded as candidates for further purification. Subfraction 1 (337.8 mg) was purified by preparative HPLC (Hypersil C_18_, isocratic 27.5% MeCN/H_2_O containing 0.01% TFA, 60 mL min^−1^) to give an enriched fraction (53.2 mg), which was further fractionated by preparative HPLC (Alltima C_18_, isocratic 25% MeCN/H_2_O containing 0.01% TFA, 60 mL min^−1^) to yield nanangenine A (**1**; *t*_R_ = 12.6 min; 25.9 mg). Subfraction 3 (942.1 mg) was purified by preparative HPLC (Hypersil C_18_, isocratic 55% MeCN/H_2_O, 60 mL min^−1^) to give an enriched fraction, which was further fractionated by preparative HPLC (Alltima C_18_, isocratic 50% MeCN/H_2_O, 60 mL min^−1^) to yield nanangenine G (**9**; *t*_R_ = 16.5 min; 211.3 mg). Subfraction 4 (632.0 mg) was purified by preparative HPLC (Hypersil C_18_, isocratic 60% MeCN/H_2_O, 60 mL min^−1^) to yield isonanangenine B (**3**; *t*_R_ = 19.1 min; 106.3 mg). Subfraction 7 (490.1 mg) was purified by preparative HPLC (Hypersil C_18_, isocratic 80% MeCN/H_2_O containing 0.01% TFA, 60 mL min^–1^) to yield nanangenine C (**4**; *t*_R_ = 11.5 min; 117.4 mg).

Initial fractionation of culture B (13.5 g) was achieved using 100 g silica gel (Davisil, Grace Discovery 50 μm) with 20 g of silica used to dry load the sample atop the column bed (55 × 250 mm). The column was eluted with CHCl_3_ (500 mL) containing increasing concentrations of MeOH. The fractions containing terpenoids (0-2% MeOH, 3.0 g) were pooled and fractionated using isocratic preparative HPLC (Zorbax C_18_, 65% MeCN/H_2_O, 60 mL min^–1^) to give five fractions containing terpenoids. Fraction 1 (785.3 mg) was purified by isocratic preparative HPLC (Zorbax C_18_, isocratic 45% MeCN/H_2_O, 20 mL min^–1^) to yield nanangenine H (**12**; *t*_R_ = 15.6 min; 41.7 mg). Fraction 2 (93.7 mg) was purified by isocratic preparative HPLC (Zorbax C_18_, isocratic 70% MeCN/H_2_O, 20 mL min^–1^) to yield nanangenine F (**8**; *t*_R_ = 12.9 min; 7.2 mg). Fraction 3 (30.6 mg) was purified by isocratic preparative HPLC (Zorbax C_18_, isocratic 70% MeCN/H_2_O, 20 mL min^−1^) to yield isonanangenine D (**6**; *t*_R_ = 15.8 min; 10.7 mg). Fraction 4 (48.8 mg) was purified by isocratic preparative HPLC (Zorbax C_18_, isocratic 70% MeCN/H_2_O, 20 mL min^−1^) to yield nanangenine D (**5**; *t*_R_ = 21.8 min; 29.9 mg). Fraction 5 (24.4 mg) was purified by isocratic preparative HPLC (Zorbax C_18_, isocratic 75% MeCN/H_2_O, 20 mL min^−1^) to yield nanangenine E (**7**; *t*_R_ = 12.8 min; 8.7 mg).

### Characterisation of compounds

**Nanangenine A (1)**: white powder; [α]_D_^20^ −205 (*c* 0.03, MeOH); UV (MeCN) λ_max_ (log ε) 200 (4.19); 212 (3.71) nm; HRMS–ESI (+, *m*/*z*): [M + Na]^+^ calcd. for C_15_H_22_NaO_5_^+^, 305.1359; found, 305.1363.

**Nanangenine B (2)**: white powder; [α]_D_^20^ −226 (*c* 0.06, MeOH); UV (MeCN) λ_max_ (log ε) 200 (4.08); 208 (3.76) nm; HRMS–ESI (+, *m*/*z*): [M + Na]^+^ calcd. for C_21_H_32_NaO_6_^+^, 403.2091; found, 403.2096.

**Isonanangenine B (3)**: white powder; [α]_D_^20^ −180 (*c* 0.04, MeOH); UV (MeCN) λ_max_ (log ε) 200 (4.42); 207 (4.21) nm; HRMS–ESI (+, *m*/*z*): [M + Na]^+^ calcd. for C_21_H_32_NaO_6_^+^, 403.2091; found, 403.2094.

**Nanangenine C (4)**: white powder; [α]_D_^20^ −289 (*c* 0.2, MeOH); UV (MeCN) λ_max_ (log ε) 200 (4.09); 208 (3.91) nm. HRMS–ESI (+, *m*/*z*): [M + Na]^+^ calcd. for C_21_H_32_NaO_5_^+^, 387.2142; found, 387.2147.

**Nanangenine D (5)**: white powder; [α]_D_^24^ −246 (*c* 0.28, MeOH); UV (MeCN) λ_max_ (log ε) 200 (3.90); 208 (3.81) nm; HRMS–ESI (+, *m*/*z*): [M + H]^+^ calcd. for C_23_H_37_O_6_^+^, 409.2585; found, 409.2579.

**Isonanangenine D (6)**: white powder; [α]_D_^24^ −234 (*c* 0.13, MeOH); UV (MeCN) λ_max_ (log ε) 200 (4.47); 206.5 (4.42) nm; HRMS–ESI (+, *m*/*z*): [M + H]^+^ calcd. for C_23_H_37_O_6_^+^, 409.2585; found, 409.2581.

**Nanangenine E (7)**: white powder; [α]_D_^24^ −285 (*c* 0.18, MeOH); UV (MeCN) λ_max_ (log ε) 200 (4.17); 206.5 (4.08) nm; HRMS–ESI (+, *m*/*z*): [M + H]^+^ calcd. for C_23_H_37_O_5_^+^, 393.2636; found, 393.2628.

**Nanangenine F (8)**: white powder; [α]_D_^24^ −268 (*c* 0.24, MeOH); UV (MeCN) λ_max_ (log ε) 200 (3.96); 203 (3.82) nm; HRMS–ESI (+, *m*/*z*): [M + H]^+^ calcd. for C_21_H_35_O_5_^+^ [M + H]^+^, 367.2479; found, 367.2483.

**Nanangenine G (9)**: white powder; [α]_D_^20^ −231 (*c* 0.05, MeOH); UV (MeCN) λ_max_ (log ε) 200 (4.17) nm; HRMS–ESI (+, *m*/*z*): [M + Na]^+^ calcd. for C_21_H_36_NaO_6_^+^ [M + Na]^+^, 407.2404; found, 407.2406.

**Nanangenine H (10)**: white powder; [α]_D_^24^ −270 (*c* 0.13, MeOH); UV (MeCN) λ_max_ (log ε) 200 (3.98) nm; HRMS–ESI (+, *m*/*z*): [M – H_2_O + H]^+^ calcd. for C_22_H_35_O_5_^+^ [M – H_2_O + H]^+^, 379.2479; found, 379.2473.

### Crystallography

Crystals suitable for single crystal X-ray diffraction analysis were obtained for **1**, **4** and **9**, providing confirmation of the spectroscopic structural elucidation. Indirect confirmation of the structures of **2** and **3** was obtained following their conversion to 4-bromobenzoyl ester derivatives, with substitution at the C-9 and C-1 hydroxy groups respectively, and the growth of crystals of these derivatives (**2b** and **3b**). The X-ray diffraction data obtained from single crystals of **4** and **9** did not allow a definitive determination of their absolute structures, however their anomalous dispersion statistics indicate that the assignments are very likely to be correct. The crystal structure of **1** has four crystallographically-independent molecules, while the structures of **2b** and **3b** have two crystallographically-independent molecules, and the structures of **4** and **9** have one. The cyclohexane rings of all molecules have chair conformations, the cyclohexene rings of all molecules have half-chair conformations, and the furan rings of all molecules have flattened envelope conformations. See Supporting Information File 1 for detailed methods.

### Genomics and bioinformatic analysis

Genomic DNA was extracted from *A. nanangensis* (see Supporting Information File 1) and sent to the Australian Genome Research Facilities for de novo genome sequencing using Illumina HiSeq 2000. A draft genome assembly of *A. nanangensis* was obtained using SPAdes v3.13.0 via the AAFTF pipeline (https://github.com/stajichlab/AAFTF). To facilitate the identification of a putative biosynthetic gene cluster, a local BLAST database was created from the resulting assembly [44]. Though Shinohara et al. list the revised annotation of AstC as AORIB40_05408, its amino acid sequence of was retrieved from AspGD [45] under the locus tag AO090026000582. AstC homologs were identified in *A. nanangensis* via the tBLASTn [44] functionality in Geneious 10.2.6 [46]. The genome sequences for drimane sesquiterpenoid producing Aspergilli were obtained from NCBI where available. This included *A. calidoustus* SF006504 (GCA_001511075.1), *A. ochraceus* fc-1 (GCA_004849945.1), *A. parasiticus* SU-1 (GCA_000956085.1), *A. sclerotiorum* HBR18 (GCA_000530345.1) and *A. ustus* 3.3904 (GCA_000812125.1). Homologous gene clusters were identified in these genomes using a custom Python script, named clusterblaster (https://github.com/gamcil/clusterblaster). A DIAMOND [47] database was built from protein sequences extracted from these genomes, and clusterblaster was used to rapidly identify biosynthetic gene cluster homologs in *A. ustus* and *A. calidoustus*. Genomes for *A. insuetus* CBS 107.25 and *A. pseudodeflectus* CBS 756.74 were obtained from the Joint Genome Institute (JGI) under genome portals Aspins1 and Asppdef1, respectively. Manual tBLASTn searches using the putative nanangenine biosynthetic gene cluster sequences were performed to identify homologs in these genomes. Homology between these clusters was visualised using another custom Python script named crosslinker (https://github.com/gamcil/crosslinker). Further details regarding clusterblaster and crosslinker are given in Supporting Information File 1.

## Supporting Information

File 1 Details of cultivation media, fractionation schemes, NMR spectra and tabulated 2D NMR data for all compounds, detailed X-ray crystallographic details and CCDC deposition numbers, bioassay procedures and genomic data.

File 2 Crystal structure information files for nanangenines A–C and G.

## References

[1] Heddergott C, Calvo A M, Latgé J P (2014). Eukaryotic Cell.

[2] Huang J-H, Lv J-M, Wang Q-Z, Zou J, Lu Y-J, Wang Q-L, Chen D-N, Yao X-S, Gao H, Hu D (2019). Org Biomol Chem.

[3] Liu X-H, Miao F-P, Qiao M-F, Cichewicz R H, Ji N-Y (2013). RSC Adv.

[4] Felix S, Sandjo L P, Opatz T, Erkel G (2013). Beilstein J Org Chem.

[5] Felix S, Sandjo L P, Opatz T, Erkel G (2014). Bioorg Med Chem.

[6] de Souza J J, Vieira I J C, Rodrigues-Filho E, Braz-Filho R (2011). Molecules.

[7] Cueto M, Jensen P R, Fenical W (2002). Org Lett.

[8] Hensens O D, Zink D, Williamson J M, Lotti V J, Chang R S L, Goetz M A (1991). J Org Chem.

[9] Zhang D, Fukuzawa S, Satake M, Li X, Kuranaga T, Niitsu A, Yoshizawa K, Tachibana K (2012). Nat Prod Commun.

[10] Afiyatullov S, Zhuravleva O I, Antonov A S, Kalinovsky A I, Pivkin M V, Menchinskaya E S, Aminin D L (2012). Nat Prod Commun.

[11] Bai Z-Q, Lin X, Wang J, Zhou X, Liu J, Yang B, Yang X, Liao S, Wang L, Liu Y (2015). Mar Drugs.

[12] Liaw C-C, Yang Y-L, Lin C-K, Lee J-C, Liao W-Y, Shen C-N, Sheu J-H, Wu S-H (2015). Org Lett.

[13] Liu Z, Liu H, Chen Y, She Z (2018). Nat Prod Res.

[14] Qi C, Bao J, Wang J, Zhu H, Xue Y, Wang X, Li H, Sun W, Gao W, Lai Y (2016). Chem Sci.

[15] Shaaban M, El-Metwally M M, Abdel-Razek A A, Laatsch H (2018). Nat Prod Res.

[16] Liu H, Edrada-Ebel R, Ebel R, Wang Y, Schulz B, Draeger S, Müller W E G, Wray V, Lin W, Proksch P (2009). J Nat Prod.

[17] Kozlovskii A G, Antipova T V, Zhelifonova V P, Baskunov B P, Ivanushkina N E, Kochkina G A, Ozerskaya S M (2017). Microbiology (Moscow, Russ Fed).

[18] Rahbæk L, Christophersen C, Frisvad J, Bengaard H S, Larsen S, Rassing B R (1997). J Nat Prod.

[19] Gould R O, Simpson T J, Walkinshaw M D (1981). Tetrahedron Lett.

[20] Hamasaki T, Kuwano H, Isono K, Hatsuda Y, Fukuyama K, Tsukihara T, Katsube Y (1975). Agric Biol Chem.

[21] Shinohara Y, Takahashi S, Osada H, Koyama Y (2016). Sci Rep.

[22] Fang W, Lin X, Zhou X, Wan J, Lu X, Yang B, Ai W, Lin J, Zhang T, Tu Z (2014). Med Chem Commun.

[23] Zhuravleva O I, Afiyatullov S S, Denisenko V A, Ermakova S P, Slinkina N N, Dmitrenok P S, Kim N Y (2012). Phytochemistry.

[24] Jansen B J M, de Groot A (2004). Nat Prod Rep.

[25] Chaudhary N K, Pitt J I, Lacey E, Crombie A, Vuong D, Piggott A M, Karuso P (2018). J Nat Prod.

[26] Lacey H J, Vuong D, Pitt J I, Lacey E, Piggott A M (2016). Aust J Chem.

[27] Li H, Gilchrist C L M, Lacey H J, Crombie A, Vuong D, Pitt J I, Lacey E, Chooi Y-H, Piggott A M (2019). Org Lett.

[28] Pitt J I, Lange L, Lacey A E, Vuong D, Midgley D J, Greenfield P, Bradbury M I, Lacey E, Busk P K, Pilgaard B (2017). PLoS One.

[29] Shiono Y, Hiramatsu F, Murayama T, Koseki T, Funakoshi T, Ueda K, Yasuda H Z (2007). Z Naturforsch, B: J Chem Sci.

[30] Gao Y, Honzatko R B, Peters R J (2012). Nat Prod Rep.

[31] Hitchman T S, Schmidt E W, Trail F, Rarick M D, Linz J E, Townsend C A (2001). Bioorg Chem.

[32] Lin H-C, Chooi Y-H, Dhingra S, Xu W, Calvo A M, Tang Y (2013). J Am Chem Soc.

[33] Christianson D W (2006). Chem Rev.

[34] Nakano C, Okamura T, Sato T, Dairi T, Hoshino T (2005). Chem Commun.

[35] Cao R, Zhang Y, Mann F M, Huang C, Mukkamala D, Hudock M P, Mead M E, Prisic S, Wang K, Lin F-Y (2010). Proteins: Struct, Funct, Bioinf.

[36] Nakano C, Hoshino T (2009). ChemBioChem.

[37] Lu Z, Wang Y, Miao C, Liu P, Hong K, Zhu W (2009). J Nat Prod.

[38] Zhou H, Zhu T, Cai S, Gu Q, Li D (2011). Chem Pharm Bull.

[39] Cohen E, Koch L, Thu K M, Rahamim Y, Aluma Y, Ilan M, Yarden O, Carmeli S (2011). Bioorg Med Chem.

[40] Hayes M A, Wrigley S K, Chetland I A N, Reynolds E E, Ainsworth A M, Renno D V, Latif M A, Cheng X-M, Hupe D J, Charlton P (1996). J Antibiot.

[41] Slack G J, Puniani E, Frisvad J C, Samson R A, Miller J D (2009). Mycol Res.

[42] Curlevski N J A, Xu Z, Anderson I C, Cairney J W G (2010). J Soils Sediments.

[43] Gao X, Xie X, Pashkov I, Sawaya M R, Laidman J, Zhang W, Cacho R, Yeates T O, Tang Y (2009). Chem Biol.

[44] Camacho C, Coulouris G, Avagyan V, Ma N, Papadopoulos J, Bealer K, Madden T L (2009). BMC Bioinf.

[45] Cerqueira G C, Arnaud M B, Inglis D O, Skrzypek M S, Binkley G, Simison M, Miyasato S R, Binkley J, Orvis J, Shah P (2014). Nucleic Acids Res.

[46] Kearse M, Moir R, Wilson A, Stones-Havas S, Cheung M, Sturrock S, Buxton S, Cooper A, Markowitz S, Duran C (2012). Bioinformatics.

[47] Buchfink B, Xie C, Huson D H (2015). Nat Methods.

