# Supplementary material for: Nanangenines: drimane sesquiterpenoids as the dominant metabolite cohort of a novel Australian fungus, Aspergillus nanangensis
Source: Beilstein J Org Chem. 2019 Nov 5;15:2631–43. doi: 10.3762/bjoc.15.256 (PMC6880815; doi:10.3762/bjoc.15.256)
Supplement: File 1 — Details of cultivation media, fractionation schemes, NMR spectra and tabulated 2D NMR data for all compounds, detailed X-ray crystallographic details and CCDC deposition numbers, bioassay procedures and genomic data. [file Beilstein_J_Org_Chem-15-2631-s001.pdf]

BEILSTEIN JOURNAL OF ORGANIC CHEMISTRY

Supporting Information

for

Nanangenines: drimane sesquiterpenoids as the dominant metabolite cohort of a novel Australian fungus, *Aspergillus nanangensis*

Heather J. Lacey, Cameron L. M. Gilchrist, Andrew Crombie, John A. Kalaitzis, Daniel Vuong, Peter J. Rutledge, Peter Turner, John I. Pitt, Ernest Lacey, Yit-Heng Chooi and Andrew M. Piggott

Beilstein J. Org. Chem. **2019,** *15,* 2631–2643. doi:10.3762/bjoc.15.256

Details of cultivation media, fractionation schemes, NMR spectra and tabulated 2D NMR data for all compounds, detailed X-ray crystallographic details and CCDC deposition numbers, bioassay procedures and genomic data

License and Terms: This is a supporting information file under the terms of the Creative Commons Attribution License (http://creativecommons.org/licenses/by/4.0). Please note that the reuse, redistribution and reproduction in particular requires that the authors and source are credited.
The license is subject to the *Beilstein Journal of Organic Chemistry* terms and conditions: (https://www.beilstein-journals.org/bjoc)

Contents

Supplementary methods..S4

Single crystal X-ray diffraction crystal structure determinations S4
Bioassay of compounds..S6
Genomic DNA extraction..S7
Bioinformatics analyses S7

Supplementary tables..S8

Supplementary figures S23

Supplementary references..S65

List of supplementary tables

Table S1. Recipes for microbiological media. S8
Table S2. Crystallographic data for **1**, **2b**, **3b**, **4** and **9** S10
Table S3. ^{1}H (500 MHz) and ^{13}C (125 MHz) NMR data for nanangenine A (**1**) in DMSO-d_6........S11
Table S4. ^{1}H (500 MHz) and ^{13}C (125 MHz) NMR data for nanangenine B (**2**) in DMSO-d_6........S12
Table S5. ^{1}H (500 MHz) and ^{13}C (125 MHz) NMR data for isonanangenine B (**3**) in DMSO-d_6 ...S13
Table S6. ^{1}H (500 MHz) and ^{13}C (125 MHz) NMR data for nanangenine C (**4**) in DMSO-d_6........S14
Table S7. ^{1}H (600 MHz) and ^{13}C (150 MHz) NMR data for nanangenine D (**5**) in DMSO-d_6........S15
Table S8. ^{1}H (600 MHz) and ^{13}C (150 MHz) NMR data for isonanangenine D (**6**) in DMSO-d_6...S16
Table S9. ^{1}H (600 MHz) and ^{13}C (150 MHz) NMR data for nanangenine E (**7**) in DMSO-d_6 ....S17
Table S10. ^{1}H (600 MHz) and ^{13}C (150 MHz) NMR data for nanangenine F (**8**) in DMSO-d_6.........18
Table S11. ^{1}H (500 MHz) and ^{13}C (125 MHz) NMR data for nanangenine G (**9**) in DMSO-d_6......S19
Table S12. ^{1}H (600 MHz) and ^{13}C (150 MHz) NMR data for nanangenine H (**10**) in DMSO-d_6....S20
Table S13. NCBI accessions of internal transcribed spacer (ITS) region sequences used in Figure S48. ....S21
Table S14. Predicted functions and best BLAST hit loci for genes in the putative nanangenine biosynthetic gene cluster (BGC). S21
Table S15. Pairwise amino acid alignment identity (%) and similarity (%) scores between putative nanangenines BGC in *A. nanangensis* MST-FP2251 and homologs in *A. ustus*, *A. calidoustus*, *A. insuetus* and *A. pseudodeflectus*..S22

List of supplementary figures

Figure S1. Fractionation scheme for Culture A (jasmine rice) S23
Figure S2. Fractionation scheme for Culture B (pearl barley)...S24
Figure S3. Olex2 depiction of one of the four crystallographically-independent molecules of nanangenine A (**1**), with 50% displacement ellipsoids and showing the intramolecular hydrogen bond. S25
Figure S4. Olex2 depiction of one of the two crystallographically-independent molecules of 9-*O*-(4-bromobenzoyl)-nanangenine B (**2b**) with 50% displacement ellipsoids. S26
Figure S5. Olex2 depiction of one of the two crystallographically-independent molecules of 1-*O*-(4-bromobenzoyl)-isonanangenine B (**3b**) with 75% displacement ellipsoids. S27

Figure S6. Olex2 depiction of nanangenine C (**4**) and a hydrogen bond-linked water molecule, with 50% displacement ellipsoids. S28
Figure S7. Olex2 depiction of nanangenine G (**9**) with 50% displacement ellipsoids and disordered sites shown with 'faded' colour. S29
Figure S8. ^{1}H NMR spectrum (500 MHz, DMSO-d_6) of nanangenine A (**1**) S30
Figure S9. ^{13}C NMR spectrum (150 MHz, DMSO-d_6) of nanangenine A (**1**) S31
Figure S10. ^{1}H NMR spectrum (500 MHz, DMSO-d_6) of nanangenine B (**2**) S32
Figure S11. ^{13}C NMR spectrum (125 MHz, DMSO-d_6) of nanangenine B (**2**) S33
Figure S12. ^{1}H NMR spectrum (500 MHz, DMSO-d_6) of isonanangenine B (**3**) S34
Figure S13. ^{13}C NMR spectrum (125 MHz, DMSO-d_6) of isonanangenine B (**3**) S35
Figure S14. ^{1}H NMR spectrum (500 MHz, DMSO-d_6) of nanangenine C (**4**) S36
Figure S15. ^{13}C NMR spectrum (125 MHz, DMSO-d_6) of nanangenine C (**4**) S37
Figure S16. ^{1}H NMR spectrum (600 MHz, DMSO-d_6) of nanangenine D (**5**) S38
Figure S17. ^{13}C NMR spectrum (150 MHz, DMSO-d_6) of nanangenine D (**5**) S39
Figure S18. ^{1}H NMR spectrum (600 MHz, DMSO-d_6) of isonanangenine D (**6**) S40
Figure S19. ^{13}C NMR spectrum (150 MHz, DMSO-d_6) of isonanangenine D (**6**) S41
Figure S20. ^{1}H NMR spectrum (600 MHz, DMSO-d_6) of nanangenine E (**7**) S42
Figure S21. ^{13}C NMR spectrum (150 MHz, DMSO-d_6) of nanangenine E (**7**) S43
Figure S22. ^{1}H NMR spectrum (600 MHz, DMSO-d_6) of nanangenine F (**8**) S44
Figure S23. ^{13}C NMR spectrum (150 MHz, DMSO-d_6) of nanangenine F (**8**) S45
Figure S24. ^{1}H NMR spectrum (500 MHz, DMSO-d_6) of nanangenine G (**9**) S46
Figure S25. ^{13}C NMR spectrum (125 MHz, DMSO-d_6) of nanangenine G (**9**) S47
Figure S26. ^{1}H NMR spectrum (600 MHz, DMSO-d_6) of nanangenine H (**10**) S48
Figure S27. ^{13}C NMR spectrum (150 MHz, DMSO-d_6) of nanangenine H (**10**) S49
Figure S28. HRMS–ESI(+) spectrum of nanangenine A (**1**) S50
Figure S29. HRMS–ESI(+) spectrum of nanangenine B (**2**) S50
Figure S30. HRMS–ESI(−) spectrum of isonanangenine B (**3**) S51
Figure S31. HRMS–ESI(−) spectrum of nanangenine C (**4**) S51
Figure S32. HRMS–ESI(+) spectrum of nanangenine D (**5**) S52
Figure S33. HRMS–ESI(+) spectrum of isonanangenine D (**6**) S52
Figure S34. HRMS–ESI(+) spectrum of nanangenine E (**7**) S53
Figure S35. HRMS–ESI(+) spectrum of nanangenine F (**8**) S53
Figure S36. HRMS–ESI(+) spectrum of nanangenine G (**9**) S54
Figure S37. HRMS–ESI(+) spectrum of nanangenine H (**10**) S54
Figure S38. UV–vis spectrum (MeCN) of nanangenine A (**1**) S55
Figure S39. UV–vis spectrum (MeCN) of nanangenine B (**2**) S55
Figure S40. UV–vis spectrum (MeCN) of isonanangenine B (**3**) S56
Figure S41. UV–vis spectrum (MeCN) of nanangenine C (**4**) S56
Figure S42. UV–vis spectrum (MeCN) of nanangenine D (**5**) S57
Figure S43. UV–vis spectrum (MeCN) of isonangenine D (**6**) S57
Figure S44. UV–vis spectrum (MeCN) of nanangenine E (**7**) S58
Figure S45. UV–vis spectrum (MeCN) of nanangenine F (**8**) S58
Figure S46. UV–vis spectrum (MeCN) of nanangenine G (**9**) S59
Figure S47. UV–vis spectrum (MeCN) of nanangenine H (**10**) S59
Figure S48. Maximum likelihood (ML; top) and Bayesian Inference (BI; bottom) trees of the internal transcribed spacer (ITS) region of *Aspergillus nanangensis* MST-FP2251 and other Aspergilli. S60
Figure S49. Taxonomic distribution of drimane sesquiterpene production across the *Aspergillus* genus. S61
Figure S50. Multiple sequence alignment of FE257_006542, AstC and orthologous proteins S62

Figure S51. Multiple sequence alignment of internal transcribed spacer (ITS) regions from *A. nanangensis* MST-FP2251, *A. janus* NRRL 1787 and an uncultured fungus previously isolated from soil near Nanango, Queensland. Pairwise identity matrix table (bottom): columns correspond to the same sequences in the same order as the rows. S63
Figure S52. Phylogeny of AstC, the sesquiterpene synthase involved in astellolide C biosynthesis, homologs identified in available genomes of drimane sesquiterpene producing species *A. insuetus, A. oryzae, A. parasiticus, A. ustus, A. nanangensis, A. calidoustus and A. pseudodeflectus.* S64

Supplementary methods

Single crystal X-ray diffraction crystal structure determinations

In each case, a suitable specimen was selected and attached to a fibre or nylon loop with Exxon Paratone N, then quenched in a cold nitrogen gas stream from an Oxford Cryosystems Cryostream. Data were collected with a four-circle kappa goniometer and CCD detector equipped SuperNova X-ray diffractometer, using mirror monochromated Cu Kα radiation generated from a microsource. Data processing was undertaken with CrysAlisPro [1] software and included a multi-scan absorption correction. Subsequent computations were carried out with the assistance of ShelXle [1], WinGX [2,3], PLATON [4,5] and XNPP [6]. The structures were obtained using ShelXT [7] and extended and refined with ShelXL [8]. In general non-hydrogen atoms in the asymmetric unit were modelled with anisotropic displacement parameters and a riding atom model was used for the hydrogen atoms. Images with displacement ellipsoid representations were generated with Olex2 [9]. Crystallographic data are summarised in Table S2.

General procedure for synthesis of 4-bromobenzoate derivatives: A solution of nanangenine to be derivatised (0.03 mmol), 4-bromobenzoyl chloride (6.6 mg, 0.03 mmol) and triethylamine (50 μL) in MeCN (950 μL) was heated at 70 °C for 4 h. The solution was washed with sodium bicarbonate solution (3 M, 50 mL) and extracted into ethyl acetate before being concentrated in vacuo. The residue was purified by preparative HPLC (Hypersil C_{18}, isocratic 100% MeCN, 20 mL min^{-1}). Crystals suitable for X-ray diffraction were grown by slow evaporation of methanolic solutions.

Nanangenine A (1): A colourless blade-like crystal of **1** was used for the data collection and the structure was obtained and modelled in the space group $P2_1$(#4). The asymmetric unit was found to contain four crystallographically-independent molecules. The hydroxy hydrogens were located in final difference maps and in general modelled with isotropic displacement parameters. The displacement parameter for one of the model hydroxy hydrogen atoms (H3O_4) was tied to that of the associated oxygen atom. In addition to an intramolecular hydrogen bond, intermolecular hydrogen bonds link molecules in discrete layers on the (002) planes of the unit cell. A depiction of one the molecules with 50% displacement ellipsoids is provided in Figure S3. The Parsons intensity quotients based Flack parameter determined by ShelXL was 0.06(3). The PLATON calculated Bayesian probability estimate that the assigned absolute structure is correct is 1.0. The probability estimate that the assignment is incorrect is 0.0, while that for an inversion twin is 8.0×10^{-51}. Accordingly, the assigned absolute structure is confirmed. CCDC reference: 1940618.

9-*O*-(4-Bromobenzoyl)-nanangenine B (2b): A colourless columnar crystal was used for the data collection and the structure was obtained in the space group $P2_1$ (#4). The asymmetric unit contains two crystallographically-independent molecules. The diffraction data are pseudo-orthorhombic and a structure can be obtained and modelled in $P2_12_12_1$ (#19), however the pendant alkyl residue is poorly resolved. Relatively large displacement envelopes on the alkyl residue mask disorder. The hydroxy hydrogen sites were located in final difference maps and modelled with isotropic displacement parameters. An Olex2 depiction of one of the molecules, with 50% displacement ellipsoids, is provided in Figure S4. The Parsons intensity quotients based Flack parameter determined by ShelXL was −0.003(7) and accordingly the assigned absolute structure is confirmed. CCDC reference: 1940620.

1-*O*-(4-Bromobenzoyl)-isonanangenine B (3b): A pale orange prismatic fragment cut from a larger crystal was used for the data collection and the structure was obtained in the space group $P2_1$(#4). The asymmetric unit contains two crystallographically independent molecules. The asymmetric unit contains two crystallographically-independent molecules, one of which exhibits minor site disorder of

the bromobenzoate residue. Two orientations were modelled for the disordered residue, with respective occupancies refined and then fixed at 0.93 and 0.07. A rigid body was applied in modelling the minor occupancy bromobenzoate sites. Isotropic displacement parameters were used for the minor occupancy sites. The hydroxy hydrogen position was located in final difference maps and refined with isotropic displacement parameters. The two independent molecules are linked by a hydrogen bond between the hydroxy residue of one and the furanone carbonyl moiety of the second. An Olex2 depiction of one of the molecules, with 75% displacement ellipsoids, is provided in Figure S5. The Parsons intensity quotients based Flack parameter determined by ShelXL refined to –0.017(5). The PLATON calculated Bayesian probability estimate that the assigned absolute structure is correct is 1.0. The probability estimate that the assignment is incorrect or is an inversion twin is 0.0. CCDC reference: 1941156.

Nanangenine C (4): A colourless prismatic crystal of **4** was used for the data collection and the structure was obtained in the space group $P2_12_12_1$ (#19). The asymmetric unit contains **4** linked by a hydrogen bond to a water molecule; the water hydrogen positions were inferred from final difference maps. A depiction of the molecule with 50% displacement ellipsoids is provided in Figure S6. The Parsons intensity quotients based Flack parameter determined by ShelXL was 0.10(9) and that of XNPP refined to 0.12(7). The PLATON calculated Bayesian probability estimate that the assigned absolute structure is correct is 1.0. The probability estimate that the assignment is incorrect is 1.0×10^{-37}, while that for an inversion twin is 1.0×10^{-6}. Accordingly, though not definitively determined, the assigned absolute structure appears likely to be correct. CCDC reference: 1940619.

Nanangenine G (9): A colourless plate like crystal of **9** was used for the data collection and the structure was obtained in the space group $P2_12_12_1$ (#19). The pedant alkyl residue of **9** is disordered about two orientations. Site occupancies were refined and then fixed at 0.6 and 0.4; isotropic displacement parameters were used for the minor occupancy sites. The hydroxy hydrogen positions for all but that of O3 were located in final difference maps and refined with isotropic displacement parameters. Adjacent molecules are linked by hydrogen bonds. A depiction of the molecule with 50% displacement ellipsoids is provided in Figure S7. The ShelXL and XNPP calculated Flack parameter refined to 0.10(10) and 0.08(9) respectively. The PLATON calculated Bayesian probability estimate that the assigned absolute structure is correct is 1.0. The probability estimate that the assignment is incorrect is 4.0×10^{-24}, while that for an inversion twin is 4.0×10^{-5}. Accordingly, though not definitively determined, the assigned absolute structure is likely correct. CCDC reference: 1940621.

Bioassay of compounds

Purified metabolites were dissolved in DMSO to provide stock solutions of 10,000 μg/mL. An aliquot of each stock solution was transferred to the first lane of Rows B to G in a 96-well microtitre plate and two-fold serially diluted with DMSO across the 12 lanes of the plate to provide a 2,048-fold concentration gradient. Bioassay medium was added to an aliquot of each test solution to provide a 100-fold dilution into the final bioassay, thus yielding a test range of 100 to 0.05 μg/mL in 1% DMSO. Row A contained no test compound (as a reference for no inhibition) and row H was uninoculated (as a reference for complete inhibition).

CyTOX is an indicative bioassay platform for discovery of antitumour actives. NS-1 (ATCC TIB-18) mouse myeloma, DU-145 (ATCC HTB-81) prostate cancer, MCF-7 (ATCC HTB-22) breast cancer, and NFF (ATCC PCS-201) human neonatal foreskin fibroblast cells were each inoculated in 96-well microtitre plates (190 μL) at 50,000 cells/mL in DMEM (Dulbecco's Modified Eagle Medium + 10% fetal bovine serum (FBS) + 1% penicillin/streptomycin (10,000 U mL^{-1} / 10,000 μg mL^{-1}, Life Technologies Cat. No. 15140122), together with resazurin (250 μg mL^{-1}; 10 μL) and incubated in 37 °C (5% CO_2) incubator. The plates were incubated for 96 h during which time the positive control wells change colour from a blue to pink colour. The absorbance of each well was measured at 605 nm using a Spectromax plate reader (Molecular Devices).

ProTOX is a generic bioassay platform for antibiotic discovery. In the present study *Bacillus subtilis* (ATCC 6633) and *Escherichia coli* (ATCC 25922) were used as indicative species for Gram-positive and Gram-negative antibacterial activity. A bacterial suspension (50 mL in 250 mL flask) was prepared in nutrient media by cultivation for 24 h at 250 rpm, 28 °C. The suspension was diluted to an absorbance of 0.01 absorbance units per mL, and 10 μL aliquots were added to the wells of a 96-well microtitre plate, which contained the test compounds dispersed in nutrient broth (Amyl) with resazurin (12.5 μg mL^{-1}). The plates were incubated at 28 °C for 24 h during which time the positive control wells change colour from a blue to light pink colour. MIC end points were determined visually. The absorbance was measured using Spectromax plate reader (Molecular Devices) at 605 nm and the IC_{50} values determined graphically.

EuTOX is a generic bioassay platform for antifungal discovery. In the present study, the yeast *Candida albicans* (ATCC 10231) was used as indicative species for antifungal activity. A yeast suspension (50 mL in 250 mL flask) was prepared in 1% malt extract broth by cultivation for 24 h at 250 rpm, 28 °C. The suspension was diluted to an absorbance of 0.005 absorbance units per mL for *C. albicans*. Aliquots (20 μL and 30 μL) of *C. albicans* were applied to the wells of a 96-well microtitre plate, which contained the test compounds dispersed in malt extract agar containing bromocresol green (50 μg mL^{-1}). The plates were incubated at 28 °C for 24 h during which time the positive control wells change colour from a blue to yellow colour. MIC end points were determined visually. The absorbance was measured using Spectromax plate reader (Molecular Devices) at 620 nm and the IC_{50} determined graphically.

Phytox is a generic bioassay platform for herbicidal discovery. In the present study, *Eragrostis tef* (teff) seed was used as indicative species for herbicidal discovery. Teff seeds (10 to 15) were dispensed using a LabTIE seed dispenser into the wells of a 96-well microtitre plate, which contained the test compounds dispersed in 200 μL of agar (1% w/v) per well. The plates were placed in a tray wrapped with a semi-opaque bag, exposed to 1600 lux (within the tray) using Power-GLO (20 W) and Sun-GLO (20 W) tubes, and incubated for 72 h at 24 °C.

Genomic DNA extraction

A. nanangensis was grown in potato dextrose broth (BD Bioscience) at room temperature. Mycelium was collected from one week-old liquid culture and ground in liquid nitrogen. The frozen ground mycelium was extracted using a buffer with 10mM Tris, 10 mM EDTA, 1% SDS and incubated at 70 °C for 20 min. This is followed by precipitation of the SDS and cell debris with equal volume of 2.8 M potassium acetate and clean-up with chloroform/isoamyl alcohol (24:1) extraction. Finally, the genomic DNA was precipitated from the supernatant using 0.7 volume isopropanol and the DNA pellet was dissolved in TE buffer. The quality of the genomic DNA was checked by gel electrophoresis.

Bioinformatics analyses

Alignment of *A. nanangensis* MST-FP2251 ITS to an undescribed sequence: *A. nanangensis* ITS sequence was retrieved from genome sequence and is deposited on GenBank (MK979278). ITS sequences of *A. janus* and an uncultured fungus were obtained from NCBI at accessions EU021598.1 and GQ921753.1, respectively. Sequences were trimmed based on length of the shortest sequence, and the multiple sequence alignment was constructed using Clustal Omega v1.2.4 [10].

Alignment of *A. nanangensis* MST-FP2251 with other Aspergilli: ITS sequences of *Aspergillus* species were obtained from NCBI (Table S13) and were aligned using MAFFT with the L-INS-i option. ML tree was constructed using RAxML-NG v0.6.0 [11] from 1000 bootstrap replicates. BI tree was constructed using MrBayes v3.2.6 [12] from 200000 generations. Both trees were constructed under the General Time Reversible (GTR) substitution model.

Alignment of AstC and homologs listed in Shinohara et al: Though Shinohara et al. list the revised annotation of AstC as AORIB40_05408, its amino acid sequence of was retrieved from AspGD [13] under the locus tag AO090026000582. AORIB40_NS.05916 was obtained from AspGD at coordinates Chr3_A_oryzae_RIB40:3512882-3513472(-). All other sequences were retrieved from NCBI via their accession codes. A multiple sequence alignment was constructed using ClustalOmega v1.2.4 [10].

Alignment of AstC to tBLASTn hits: Local BLAST databases were constructed inside Geneious 10.2.6 and AstC homologs were identified using tBLASTn. A multiple sequence alignment of the AstC homologs was constructed using MUSCLE v3.8.31 [14] and a phylogenetic tree was constructed under the JTT model using FastTree 2.1.5 [15].

Custom Python scripts: Homologous gene clusters were identified using a custom Python script, named clusterblaster (https://github.com/gamcil/clusterblaster). Briefly, the script takes as input a set of query sequences in FASTA format (presumably clustered), searches a pre-constructed DIAMOND [16] database consisting of protein sequences obtained from NCBI, then leverages the 'identical protein group' functionality of NCBI Entrez to ascertain the original genomic context of hit proteins (i.e. start and end on an assembly scaffold). Hits are then grouped by scaffold and ordered by location; those that meet user-defined clustering thresholds (maximum intergenic distance, minimum number of proteins conserved) are reported.

Gene cluster homology was visualised using crosslinker (https://github.com/gamcil/crosslinker). Briefly, crosslinker takes as input GenBank files of gene clusters, performs pairwise alignments between all sets of protein sequences, then draws a to-scale SVG figure depicting gene cluster homology with shaded boxes linking homologous proteins, the darkness of which indicates the degree of amino acid identity.

Supplementary tables

Table S1. Recipes for microbiological media.

**Glycerol Casein Agar (AC)**	
Ingredient	*Quantity*
Glycerol (Chem-Supply)	30 g
Casein peptone (Amyl)	2 g
K_2HPO_4(Chem-Supply)	1 g
NaCl (Chem-Supply)	1 g
$MgSO_4 \cdot 7H_2O$ (AnalaR)	0.5 g
Trace element solution (mL)*	5 mL
Distilled H_2O	1000 mL
Bacteriological agar (Amyl)	20 g
Autoclave	
***Trace element solution**	
$CaCl_2 \cdot 2H_2O$	3 g
$FeC_6O_7H_5$	1 g
$MnSO_4$	0.2 g
$ZnCl_2$	0.1 g
$CuSO_4 \cdot 5H_2O$	0.025 g
$Na_2B_4O_7 \cdot 10H_2O$	0.02 g
$CoCl_2$	0.004 g
$Na_2MoO_4 \cdot 2H_2O$	0.01 g
Deionised water	1000 mL
Filter sterilise	

**Czapeks Agar (CZ)**	
Ingredient	*Quantity*
Czapeks Dox Media (Oxoid)	99.88 g
Distilled water	2200 mL

**Malt Extract Agar (MA)**	
Ingredient	*Quantity*
Bacteriological peptone (Difco)	3 g
Malt extract (Amyl)	60 g
Bacteriological glucose (Amyl)	60 g
Distilled water	1000 mL
Adjust pH to 5.5	
Bacteriological agar (Amyl)	20 g
Autoclave	

**Yeast Extract Sucrose Agar (YS)**	
Ingredient	*Quantity*
Yeast Extract (Difco)	20 g
Sucrose (Amyl)	150 g
Bacteriological Agar (Amyl)	20 g

Distilled water	1000 mL
Autoclave	

**Cracked Wheat (BL)**	
Ingredient	*Quantity*
Cracked wheat	46 g
Distilled water	30 mL
Autoclave	

**Pearl Barley (PB)**	
Ingredient	*Quantity*
Pearl barley	48 g
Distilled water	35 mL
Autoclave	

**Jasmine Rice (JR)**	
Ingredient	*Quantity*
Jasmine rice	50 g
Distilled water	25 mL
Autoclave	

**Basmati rice (BS)**	
Ingredient	*Quantity*
Basmati rice	44 g
Distilled water	30 mL
Autoclave	

Table S2. Crystallographic data for **1**, **2b**, **3b**, **4** and **9**.

	Nanangenine A (**1**)	9-*O*-(4-Bromobenzoyl)-nanangenine B (**2b**)	1-*O*-(4-Bromobenzoyl)-isonanangenine B (**3b**)	Nanangenine C (**4**)	Nanangenine G (**9**)
Formula	$C_{15}H_{22}O_5$	$C_{28}H_{35}BrO_7$	$C_{28}H_{35}BrO_7$	$C_{21}H_{34}O_6$	$C_{21}H_{36}O_6$
Molecular Weight	282.32	563.47	563.47	382.48	384.50
Crystal System	monoclinic	monoclinic	monoclinic	orthorhombic	orthorhombic
Space Group	$P2_1$(#4)	$P2_1$(#4)	$P2_1$(#4)	$P2_12_12_1$ (#19)	$P2_12_12_1$ (#19)
a (Å)	15.7957(2)	7.7558(2)	14.88851(10) Å	7.6270(4)	6.4497(2)
b (Å)	10.30070(10)	14.0642(2)	6.10835(4) Å	9.5926(3)	7.9709(4)
c (Å)	19.6228(2)	24.5340(3)	29.4427(3) Å	28.6181(8)	40.3829(19)
β	113.6310(10)º	90.460(2)º	101.3693(7)º	-	-
V (Å^3)	2925.04(6)	2676.06(9)	2625.10(4)	2093.76(13)	2076.08(16)
D_c (g cm^{-3})	1.282	1.399	1.426 g	1.213	1.230
Z	8	4	4	4	4
Crystal Size (mm)	0.295×0.080×0.039	0.168×0.040×0.033	0.134×0.120×0.044	0.382×0.229×0.059	0.188×0.187×0.016
Crystal Colour	colourless	colourless	colourless	colourless	colourless
Crystal Habit	blade	columnar	prism	prism	late
Temperature (Kelvin)	150.0(5)	100.0(5)	100.0(1)	150.0(5)	150.0(5)
λ(Cu Kα, Å)	1.5418	1.54184	1.5418	1.54184	1.5418
μ(Cu Kα, mm^{-1})	0.788	2.462	2.509	0.713	0.720
$T_{max,max}$ (multi-scan)	0.884, 1.00	0.934, 1.00	0.874, 1.00	0.696, 1.00	0.737, 1.00
$2\theta_{max}$	152.59º	153.23º	152.64º	148.88º	171.94º
hkl range	–19 18, –12 12, –24 24	–8 7, –17 17, –30 30	–18 18, –7 7, –36 34	–8 9, –11 11, –35 35	–8 8, –9 9, –50 50
N	65936	52872	74047	36004	47388
N_{ind}	11984 (R_{merge} 0.0338)	10615 (R_{merge} 0.0506)	10685 (R_{merge} 0.0448)	4173 (R_{merge} 0.1085)	4335 (R_{merge} 0.0772)
N_{iobs}	11783(I > 2σ(I))	9834(I > 2σ(I))	10457(I > 2σ(I))	3841(I > 2σ(I))	3937(I > 2σ(I))
N_{var}	781	665	672	252	281
Residuals $R1(F)$, $wR2(F^2)$	0.0403, 0.1112	0.0522, 0.1327	0.0337, 0.0844	0.0474, 0.1342	0.0555, 0.1350
GoF(all)	1.301	1.220	1.159	1.361	1.267
Residual Extrema (e$^-$Å^{-3})	–0.260, 0.203	-0.573, 1.496	–0.672, 1.215	–0.298, 0.296	–0.322, 0.335

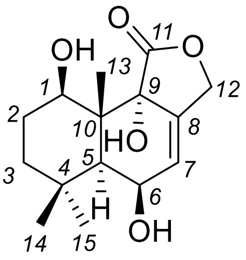

Table S3. ^{1}H (500 MHz) and ^{13}C (125 MHz) NMR data for nanangenine A (**1**) in DMSO-d_6.

Pos.	δ_C	δ_H, mult. (J in Hz)	HMBC	COSY	NOESY
1	69.0	4.12, dd (8.4, 7.8)	2, 3, 9, 13	1-OH, 2	1-OH, 2, 5, 9-OH
2	26.2	1.54, m	1, 4, 5, 14	1, 3	1, 3, 13
3	41.5	1.22, m	4, 14	2	1, 2, 6-OH, 13, 15
4	33.6				
5	45.0	1.52, d (5.0)	3, 4, 6, 9, 12, 13	6	1, 3, 6, 9-OH, 14, 15
6	63.3	4.30, m		5, 6-OH	5, 6-OH, 7, 15
7	128.2	5.92, m	5, 9	6, 12a/b	6, 6-OH, 12b
8	131.1				
9	76.1				
10	42.4				
11	179.3				
12a	70.6	5.00, ddd (12.2, 2.5, 2.4)	7, 8, 9, 11	7, 12b	6, 7
12b		4.91, ddd (12.2, 1.3, 1.2)		7, 12a	7
13	12.6	0.92, s	1, 5, 9, 10,		2, 3, 1-OH
14	24.3	1.23, s			1, 2, 15
15	31.9	1.02, s	3, 4, 5, 15		5, 6, 14
1-OH		4.76, s	1	1	1, 2, 13
6-OH		4.81, br d (5.8)	6	6	6, 7, 9-OH, 14
9-OH		6.50, s	3, 8, 9, 11		1, 5, 6-OH,

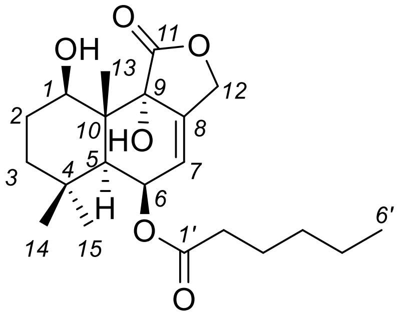

Table S4. ^{1}H (500 MHz) and ^{13}C (125 MHz) NMR data for nanangenine B (**2**) in DMSO-d_6.

Pos.	δ_C	δ_H, mult. (*J* in Hz)	HMBC
1	68.8	4.19, m	2, 9, 13
2	26.0	1.57, m[b]	
3	41.4	1.29, m[c]	1, 2, 4, 5,
4	33.1		
5	43.9	1.87, d (4.94)	1, 4, 6, 9, 10, 13, 14, 15
6	66.1	5.47, m	5, 7, 8, 1'
7	122.0	5.88, m	5, 6, 9, 12
8	135.6		
9	75.7		
10	42.5		
11	178.5		
12a	70.4	5.03, m	6, 7, 8, 9, 11
12b		4.94, m	6, 7, 8, 9, 11
13	12.3	0.95, s	1, 5, 9, 10
14	24.1	1.07, s	3, 4, 5, 15
15	30.6	0.91, s	3, 4, 5, 14
1'	172.5		
2'a	34.1	2.32, m	1', 3', 4'
2'b		2.27, m	1', 3', 4'
3'	24.0	1.55, m[b]	1', 4', 5'
4'	31.6	1.23, m[c]	3', 5'
5'	21.8	1.27, m[c]	4', 6'
6'	13.8	0.84, t (7.12)	4', 5'
1-OH		4.73, br s	1, 2, 10
9-OH		6.78, br s	8, 9, 10, 11

[b, c] indicates overlapping resonances.

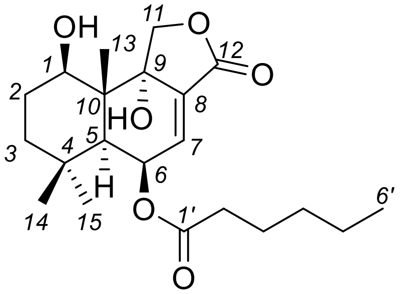

Table S5. ^{1}H (500 MHz) and ^{13}C (125 MHz) NMR data for isonanangenine B (**3**) in DMSO-d_6.

Pos.	δ_C	δ_H, mult. (J in Hz)	HMBC
1	69.2	3.96, ddd (11.4, 4.9, 4.9)	9, 10, 13
2a	27.7	1.58, m^b	
2b		1.53, m	
3a	42.2	1.31, m^c	1, 2, 4, 5, 14
3b		1.24, m	1, 4, 5, 14
4	32.4		
5	44.6	1.99, d (5.07)	4, 10, 13, 14, 15
6	66.6	5.62, dd (4.5, 4.4)	5, 7, 8, 9, 1'
7	132.1	6.46, d (3.88)	4, 5, 6, 8, 9, 12
8	133.6		
9	75.6		
10	43.7		
11a	76.9	4.43, d (10.4)	9, 10, 12
11b		4.20, d (10.4)	8, 9, 12, 13
12	169.1		
13	12.7	0.96, s	1, 5, 9, 10
14	24.6	1.09, s	3, 4, 5, 15
15	33.3	0.93, s	4, 5, 14
1'	172.7		
2'a	34.3	2.36, m	1', 3', 4'
2'b		2.29, m	1', 3', 4'
3'	24.3	1.55, m^b	4', 5'
4'	31.0	1.25, m^c	3', 5'
5'	22.2	1.26, m^c	4', 6'
6'	14.2	0.84, t (6.8)	4', 5'
1-OH		4.65, br s	1, 10
9-OH		5.64, br s	9, 11

$^{b, c}$ indicates overlapping resonances.

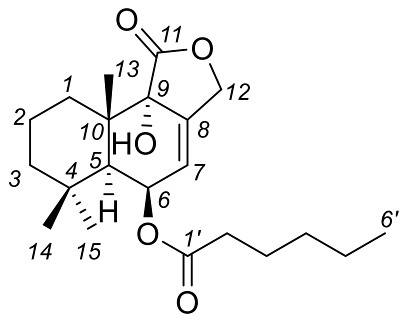

Table S6. ^{1}H (500 MHz) and ^{13}C (125 MHz) NMR data for nanangenine C (**4**) in DMSO-d_6.

**Pos.**	**δ_C**	**δ_H, mult. (*J* in Hz)**	**HMBC**
1	29.9	1.95, dd (13.9, 4.3)	2, 9, 13
2a	17.8	1.58, m	
2b		1.46, m	
3	44.8	1.33, m	1, 2, 4, 5,
4	33.7		
5	44.5	1.98, d (4.9)	4, 6, 9, 10, 13, 14, 15
6	66.5	5.48, m	5, 7, 8, 1'
7	121.8	5.76, m	5, 6, 9, 12
8	136.9		
9	73.6		
10	37.6		
11	174.8		
12	68.7	4.80, m	7, 8, 9, 11
13	18.3	1.01, s	1, 5, 9, 10
14	24.6	1.09, s	3, 4, 5, 15
15	32.5	0.92, s	3, 4, 5, 14
1'	172.7		
2'	34.5	2.28, m	1', 3', 4'
3'	24.3	1.53, m	1', 4', 5'
4'	31.0	1.26, m^b	3', 5'
5'	22.2	1.26, m^b	4', 6'
6'	14.1	0.84, t (7.1)	4', 5'
9-OH		6.30, br s	8, 9, 10, 11

^{b} indicates overlapping resonances.

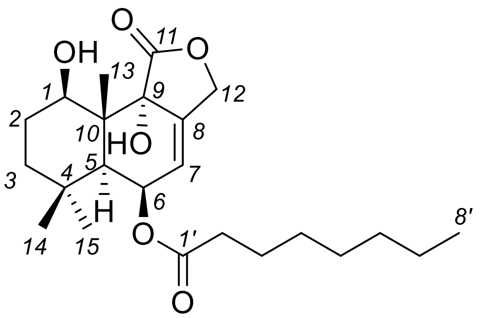

Table S7. ^{1}H (600 MHz) and ^{13}C (150 MHz) NMR data for nanangenine D (**5**) in DMSO-d_6.

**Pos.**	**δ_C**	**δ_H, mult. (*J* in Hz)**	**HMBC**	**COSY**	**ROESY**
1	68.7	4.17, ddd (10.9, 5.9, 0.9)	2, 3, 9, 10, 13	2, 1-OH	1-OH, 2, 3, 5, 9-OH
2	25.9	1.57, m	1, 3, 4	1, 3	1, 3, 13, 14
3	41.3	1.28, m	1, 5, 14	2	1, 2, 5, 14, 15
4	33.1				
5	43.8	1.87, d (4.9)	4, 6, 9, 10, 13, 14, 15	5, 6	1, 3, 6, 9-OH, 15
6	66.0	5.46, m	7, 8, 10, 1'	5, 7, 12a/b	5, 7, 15
7	121.9	5.88, m	5, 6, 8, 9, 11	6, 12a/b	6, 12b
8	135.5				
9	75.6				
10	42.4				
11	178.4				
12a	70.3	5.03, ddd (12.8, 4.8, 2.5)	6, 7, 8, 11	6, 7, 12b	12b
12b		4.94, ddd (12.8, 1.7, 1.2)	6, 7, 8, 9, 11	6, 7, 12a	7, 12a
13	12.2	0.94, s	1, 5, 9, 10		1-OH, 2
14	24.0	1.06, s	3, 5, 15		2, 3, 15
15	31.5	0.90, s	3, 4, 5, 14		3, 5, 6, 14
1'	172.2				
2'a	34.0	2.33, m	1', 3', 4'	2'b, 3'	2'b
2'b		2.27, m	1', 3', 4'	2'a, 3'	2'a
3'	24.2	1.53, m	2', 4', 5'	4'	4'
4'	28.2	1.24, m	3', 5', 6'	3'	3'
5'	28.3	1.24, m	3', 6'	6'	
6'	31.0	1.22, m	7', 8'	5', 7', 8'	
7'	21.9	1.24, m	6'	6', 8'	8'
8'	13.9	0.84, t (7.9)	6', 7'	7'	7'
1-OH		4.71, d (0.9)	1, 2, 10	1	1, 13
9-OH		6.77 s	8, 9, 10, 11		1, 5

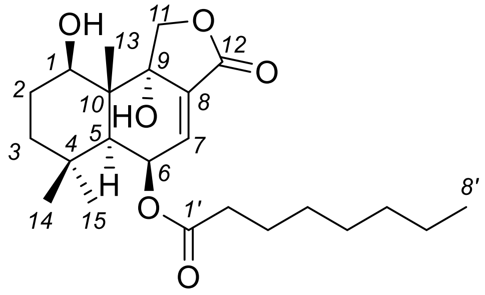

Table S8. ^{1}H (600 MHz) and ^{13}C (150 MHz) NMR data for isonanangenine D (**6**) in DMSO-d_6.

Pos.	δ_C	δ_H, mult. (*J* in Hz)	HMBC	COSY	ROESY
1	68.6	3.95, ddd (12.0, 5.9, 3.9)	3, 9, 10, 13	2, 1-OH	1-OH, 2, 3b, 5, 9-OH
2	27.2	1.53, m	1, 3	1, 3	1, 1-OH, 14
3a	41.7	1.31, ddd (13.3, 3.5, 3.5)	1	2	3b, 14
3b		1.23, m			2, 3a, 5
4	32.9				
5	44.1	1.99, d (5.2)	3, 6, 9, 10, 13, 14, 15	6, 13 (wk)	1, 3b, 6, 9-OH, 15
6	66.0	5.59, dd (5.1, 3.9)	7, 8, 10, 1'	5, 7, 11a	5, 7, 15
7	131.6	6.45, d (3.9)	5, 8, 9, 12	6	6
8	133.2				
9	75.0				
10	43.2				
11a	76.4	4.41, dd (10.2, 0.5)	9, 10	11b	11b, 13
11b		4.19, br d (10.2)	8, 9, 12	11a	11a
12	168.6				
13	12.3	0.94, s	1, 9, 10	5 (wk)	1-OH, 11a, 14
14	24.2	1.08, s	3, 4, 5, 15	14 (wk)	2, 3a, 13
15	31.9	0.91, s	3, 4, 5, 14	15 (wk)	6
1'	172.2				
2'a	33.9	2.36, m	1', 3', 4'	2'b, 3'	2'b
2'b		2.27, m	1', 3', 4'	2'a, 3'	2'a
3'	24.2	1.53, m	1', 4', 5'	2'a, 2'b, 4'	4'
4'	28.2	1.23, m	2', 3'	3'	3'
5'	28.2	1.23, m	6'	6'	
6'	31.0	1.21, m		5', 7', 8'	
7'	21.9	1.23, m	5', 6', 8'	8'	8'
8'	13.9	0.83, t (7.2)	6', 7'	7'	7'
1-OH		4.62, d (5.1)	1, 2, 10	1	1, 2, 13
9-OH		5.61 br s	8, 9, 11		1, 5

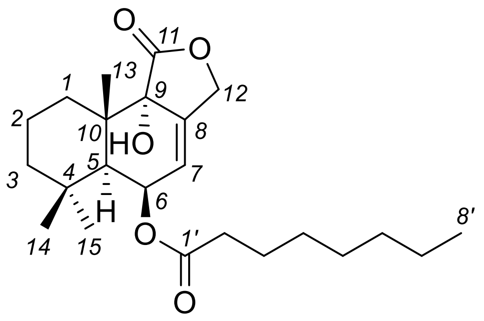

Table S9. ^{1}H (600 MHz) and ^{13}C (150 MHz) NMR data for nanangenine E (**7**) in DMSO-d_6.

Pos.	δ_C	δ_H, mult. (J in Hz)	HMBC	COSY	ROESY
1a	29.4	1.95, ddd (15.0, 13.5, 4.3)	3, 10, 13	1b, 2a/2b	1b
1b		1.81, dm (13.5)	3, 10, 13	1a, 2a/2b	1a, 13
2a	17.4	1.59, m		1a/b, 3a/b	2b, 13, 14
2b		1.47, m		1a/b, 3a/b	2a
3a	44.3	1.33, dm (13.0)		2a/b, 3b	3b, 14
3b		1.19, m		2a/b, 3a	3a, 5
4	33.3				
5	44.0	1.97, d (4.9)	4, 9, 10, 13, 14	6	3b, 6, 9-OH, 15
6	66.0	5.47, m	7, 10	5, 7, 12a,	5, 7, 15
7	121.3	5.77, m	3, 5, 9, 12	6, 12a	6, 12b
8	136.5				
9	73.1				
10	37.2				
11	174.3				
12a	68.2	4.87, ddd (12.8, 2.5, 2.5)	7, 8, 11	6, 7, 12b	12b
12b		4.74, ddd (12.8, 1.2, 1.2)	7, 8, 9, 11	12a	7, 12a, 13
13	18.1	0.99, s	1, 5, 9, 10		1b, 2a, 12b, 14
14	24.2	1.08, s	3, 4, 5, 15	14	2a, 3a, 13, 15
15	32.1	0.91, s	3, 4, 5, 14	15	5, 6, 14
1'	172.2				
2'a	34.0	2.32, m	1', 3', 4'	2'b	2'b, 4'
2'b		2.25, m	1', 3', 4'	2'a	2'a, 4'
3'	24.2	1.52, m	1', 2', 4', 5'	4'	2'a, 2'b, 4'
4'	28.3	1.24, m	5', 6'	3'	3'
5'	28.2	1.24, m	4', 6'	6'	
6'	31.0	1.21, m	4', 5'		
7'	21.9	1.24, m	5', 6'	8'	8'
8'	13.9	0.84, t (7.2)	6', 7'	7'	7'
9-OH		6.24 s	8, 9, 10, 11		5

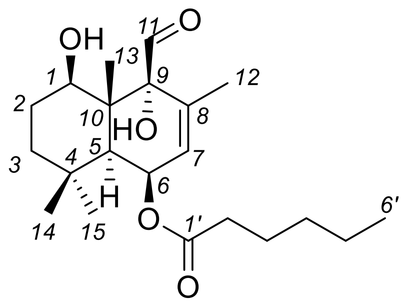

Table S10. ^{1}H (600 MHz) and ^{13}C (150 MHz) NMR data for nanangenine F (**8**) in DMSO-d_6.

Pos.	δ_C	δ_H, mult (*J* in Hz)	HMBC	COSY	ROESY
1	68.5	3.88, ddd (11.9, 5.8, 5.8)	9, 10, 13	2a, 1-OH	1-OH, 2b, 3a, 5, 9-OH
2a	27.3	1.51, m	1, 3	1	3b, 9-OH, 13, 14
2b		1.47, m	1, 3		1-OH, 3a
3a	41.6	1.26, m	1	3b	1, 2b, 14
3b		1.21, m		3a	2a, 5
4	32.8				
5	43.5	1.84, d (4.7)	4, 9, 10, 13, 14	6	1, 3b, 6, 9-OH, 15
6	66.5	5.37, m	7, 8, 10, 1'	5, 7, 12	5, 7, 15
7	124.0	5.53, dq (5.1, 1.5)	5, 6, 9, 12	6, 12	6, 12
8	137.2				
9	80.1				
10	47.0				
11	203.3	9.49, br s	7 (wk), 8 (wk), 9	9-OH	1-OH, 9-OH, 12, 13
12	19.5	1.51, dd, (1.3,1.3)	7, 8, 9	6, 7	7, 11
13	11.5	1.06, s	1, 9, 10		2a, 11
14	24.3	1.05, s	3, 4, 5, 15		15, 2'a, 2'b,
15	31.8	0.89, s	3, 4, 5, 14		3a, 5, 6, 14
1'	172.3				
2'a	34.1	2.30, m	1', 3', 4'	3'	14, 3', 4'
2'b		2.23, m	1', 3', 4'	3'	14, 3', 4'
3'	23.9	1.53, m	1', 2', 4', 5'	2'a/b, 4'	2'a, 2'b
4'	30.6	1.25, m	5'	3', 6'	2'a, 2'b
5'	21.7	1.26, m	4'	3', 6'	6'
6'	13.7	0.84, t (7.0)	4', 5'	5'	5'
1-OH		4.74, d, (5.7)	1, 2, 10	1	1, 11
9-OH		5.46, s	8, 9, 10, 11	11	1, 2a, 5, 11

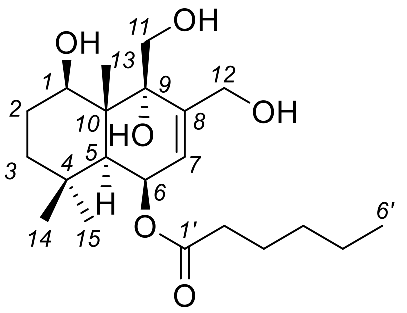

Table S11. ^{1}H (500 MHz) and ^{13}C (125 MHz) NMR data for nanangenine G (**9**) in DMSO-d_6.

**Pos.**	**δ_C**	**δ_H, mult. (*J* in Hz)**	**HMBC**	**COSY**	**NOESY**
1	69.1	3.93 dd (11.2, 4.3)		1-OH, 2	2, 3, 9-OH
2	28.3	1.56, m^b	15	1, 3	1, 3
3	41.6	1.23, m^c	1, 2, 4, 5,	2	1, 2, 5, 14
4	33.5				
5	44.9	1.83, d (4.4)	4, 10, 13, 15	6	1, 3, 6, 9-OH, 15
6	67.0	5.41, m	7, 8, 10, 1'	7	5, 7
7	121.4	5.78, br d (5.3)	5, 6, 8, 9, 11	6, 12a	6, 12a, 12-OH
8	144.1				
9	75.6				
10	45.9				
11a	62.2	3.73, m	8, 9, 10	11b, 11-OH	1-OH, 9-OH, 11b, 11-OH, 12a
11b		3.67, m	8, 9, 10	11a, 11-OH	9-OH, 11a, 11-OH, 12a
12a	61.0	4.05, m	7, 8, 12-OH	7, 12b, 12-OH	7, 11b, 12-OH
12b		4.02, m	7, 8, 12-OH	12a, 12-OH	7, 11b, 12-OH
13	12.5	1.06, s	1, 5, 9		2, 11a
14	24.4	1.03, s	3, 4, 5		2, 15, 2'a
15	32.4	0.87, br s	3, 5, 15		3, 5, 6, 14
1'	172.7				
2'a	34.5	2.24, m	1', 3', 4'	3'	3', 5'
2'b		2.21, m	1', 3', 4'	3'	3'
3'	24.2	1.53, m^b	4', 5'	2'a, 2'b, 4'	2'a
4'	30.9	1.25, m^c	3', 5', 6'	3', 5'	2'a, 6'
5'	22.0	1.26, m^c	3', 6'	4', 6'	6'
6'	13.9	0.83, t (7.1)	4', 5'	5'	3', 5'
1-OH		5.07, br s	1, 2, 10	1	9-OH, 11-OH, 12-OH
9-OH		4.41, br s	8, 9, 10, 12		1, 1-OH, 5, 11a, 11-OH, 12-OH
11-OH		5.27, br s	8, 11	11a, 11b	1-OH, 9-OH, 12-OH
12-OH		4.88, br s		12a, 12b	1-OH, 9-OH, 11-OH, 12b

$^{b, c}$ indicates overlapping resonances.

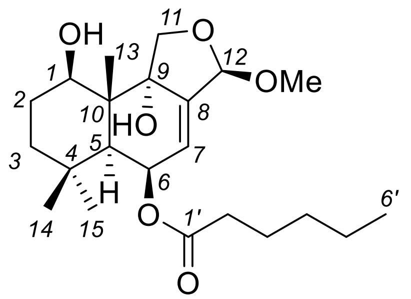

Table S12. ^{1}H (600 MHz) and ^{13}C (150 MHz) NMR data for nanangenine H (**10**) in DMSO-d_6.

Pos.	δ_C	δ_H, mult. (J in Hz)	HMBC	COSY	ROESY
1	68.4	3.93, ddd (11.8, 5.4, 5.0)	9, 10, 13	2a, 1-OH	1-OH, 2a, 2b, 3b, 5, 9-OH
2a	27.5	1.53, m	1, 3, 4	1, 2b	3b
2b		1.48, m		2a, 3a	1, 1-OH, 3a
3a	41.9	1.27, m	1	2b	2b, 14
3b		1.22, m			2a
4	32.8				
5	44.3	1.95, d (4.0)	4, 9, 10, 14, 15	6, 13	1, 3b, 6, 9-OH, 15
6	67.1	5.46, ddd (5.0, 3.9, 1.4)	7, 8, 10, 12, 1'	5, 7	5, 7, 15
7	120.3	5.57, dd (3.9,1.4)	5, 9, 12	6, 12	6
8	145.0				
9	78.3				
10	43.5				
11a	74.5	3.84, d (9.7)	7, 9	11b	11b, 13
11b		3.76, d (9.7)	7, 8, 9, 12	11a	1-OH, 11a
12	101.9	5.31, t (1.4)	7, 9, 11, 12-OMe	6, 7	9-OH, 12-OMe
13	11.7	0.92, s	1, 5, 9, 10	5	1-OH, 11a, 15, 3'
14	24.3	1.08, s	3, 4, 5, 15	14	3a, 13, 15, 3'
15	32.1	0.89, s	3, 4, 5, 14	15	5, 6, 14
1'	172.3				
2'a	34.1	2.32, m	1', 3', 4'	3'	
2'b		2.23, m	1', 3', 4'	3'	
3'	24.0	1.52, m	1', 2', 4', 5', 6'	2'a/b, 4'	13, 14
4'	30.5	1.24, m	5',	3'	
5'	21.7	1.25, m	4'	6'	6'
6'	13.9	0.84, t (7.1)	4', 5'	5'	5'
12-OMe	54.1	3.26, s	12		12
1-OH		4.37, d (5.4)	1, 2, 10	1	1, 2b, 11b, 13
9-OH		4.93, s	8, 9, 10, 11		1, 5, 12

Table S13. NCBI accessions of internal transcribed spacer (ITS) region sequences used in Figure S48.

Organism	NCBI accession
Aspergillus flavus CBS 569.65	AF027863
Aspergillus tubingensis NRRL 4875	EF661193
Aspergillus niger CBS 554.65	EF661186
Aspergillus terreus CBS 601.65	EF669586
Aspergillus carneus CBS 494.65	EF669611
Aspergillus brevijanus CBS 111.46	EF669582
Aspergillus janus CBS 118.45	EF669578
Aspergillus nanangensis MST-FP2251	MK979278

Table S14. Predicted functions and best BLAST hit loci for genes in the putative nanangenine biosynthetic gene cluster (BGC).

Scaffold_3: 476387-509910	Size (nt / aa)	Predicted function	Best BLAST loci (identity/coverage %)
FE257_006540	11080/3467	Oxidoreductase	*A. heteromorphus* BO70DRAFT_410469 (63.92/90)
FE257_006541	7788/2384	HR-PKS	*A. ustus* 00536 (66.74/99)
FE257_006542	1773/543	Haloacid dehydrogenase-like	*A. ustus* 00537 (66.30/99)
FE257_006543	1778/519	FAD-binding oxidoreductase	*A. ustus* 00538 (60.58/98)
FE257_006544	1580/526	Cytochrome P450	*A. ustus* 00539 (75.25/96)
FE257_006545	1003/312	Hydrolase	*A. calidoustus* ASPCAL02981 (72.46/97)
FE257_006546	923/307	Short-chain dehydrogenase	*A. lentulus* ALT_1008 (83.55/98)
FE257_006547	2023/460	Cytochrome P450	*T. stipitatus* TSTA_007840 (67.43/100)

Table S15. Pairwise amino acid alignment identity (%) and similarity (%) scores between putative nanangenines BGC in *A. nanangensis* MST-FP2251 and homologs in *A. ustus*, *A. calidoustus*, *A. insuetus* and *A. pseudodeflectus*.

***A. nanangensis* MST-FP2251 (Locus tag: FE257_)**									**BGC similarity (%) / BGC conservation (%)**
Locus tag	006540	006541	006542	006543	006544	006545	006546	006547	
Protein length	3467	2384	543	519	526	312	307	460	
***A. ustus* CBS 3.3904 (Locus tag: HK57_)**									
Gene no.		00536	00537	00538	00539	00540	00541		64.59/75
Identity (%)		66.26	64.53	59.2	65.48	61.43	62.58		
Similarity (%)		77.2	74.15	73.62	74.83	70.25	74.54		
***A. calidoustus* CBS 12160 (Locus tag: ASPCAL)**									
Gene no.		02977	02978	02979	02980	02981	02982		65.24/75
Identity (%)		66.4	61.66	56.17	69.55	70.83	64.87		
Similarity (%)		76.92	73.42	73.24	79.7	83.65	76.58		
***A. insuetus* CBS 107.25 (JGI Portal: Aspins1)**									
Protein ID		305363	294198	315989	315991	270963	270964		64.72/75
Identity (%)		65.89	60.77	54.68	69.29	71.15	65.19		
Similarity (%)		76.94	70.35	70.55	79.4	84.29	76.58		
***A. pseudodeflectus* CBS 756.74 (JGI Portal: Asppdef1)**									
Protein ID		276134	267293	276137	276138				55.74/50
Identity (%)		66.19	61.66	54.2	69.36				
Similarity (%)		76.63	73.24	70.23	79.51				

Total BGC similarity (%) and conservation (%) were calculated as follows: similarity = sum(sequence length × identity) / maximum score, where maximum score = sum(sequence length × 100); and conservation = number of conserved genes / number of total genes.

Supplementary figures

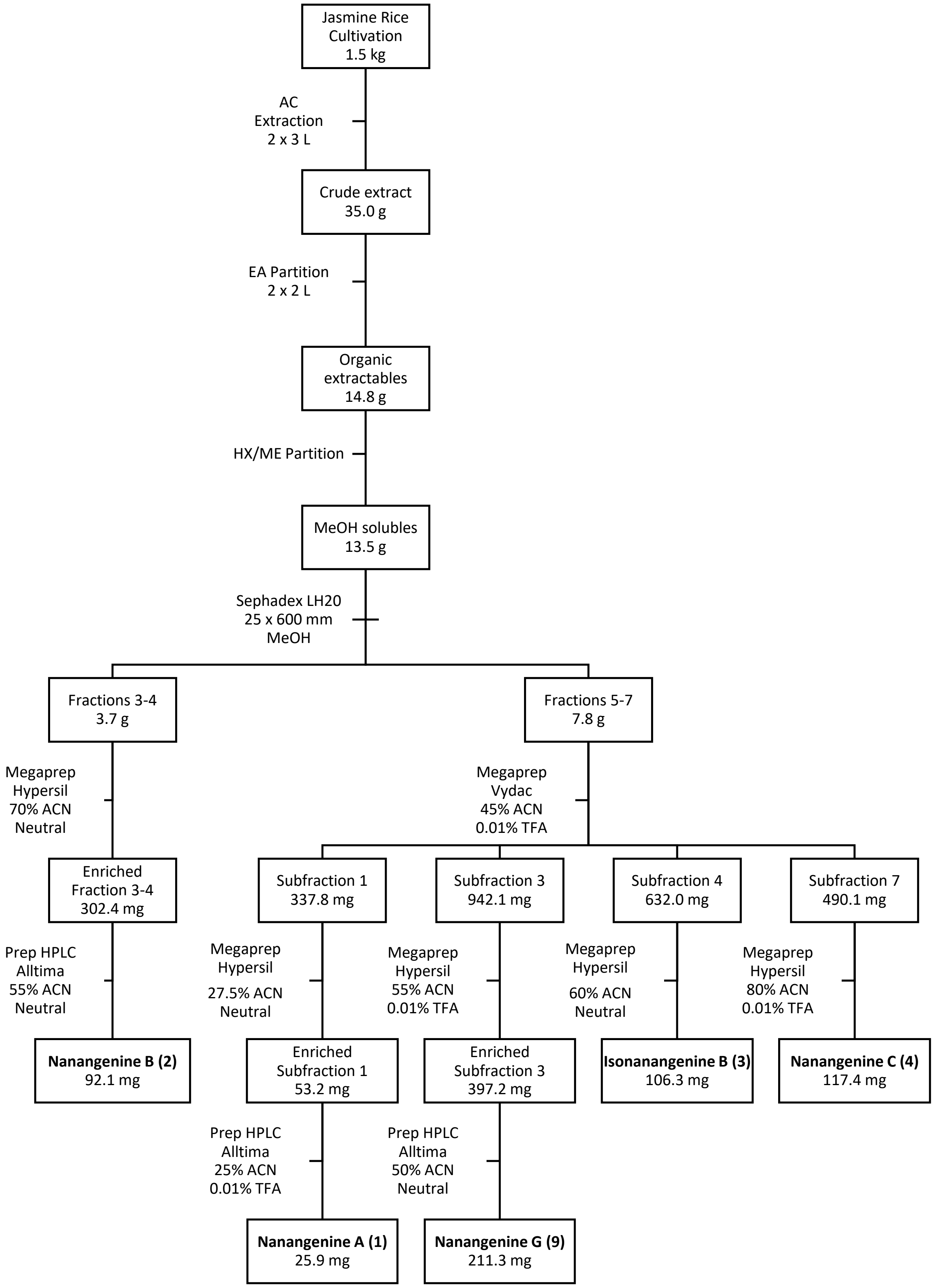

Figure S1. Fractionation scheme for culture A (jasmine rice)

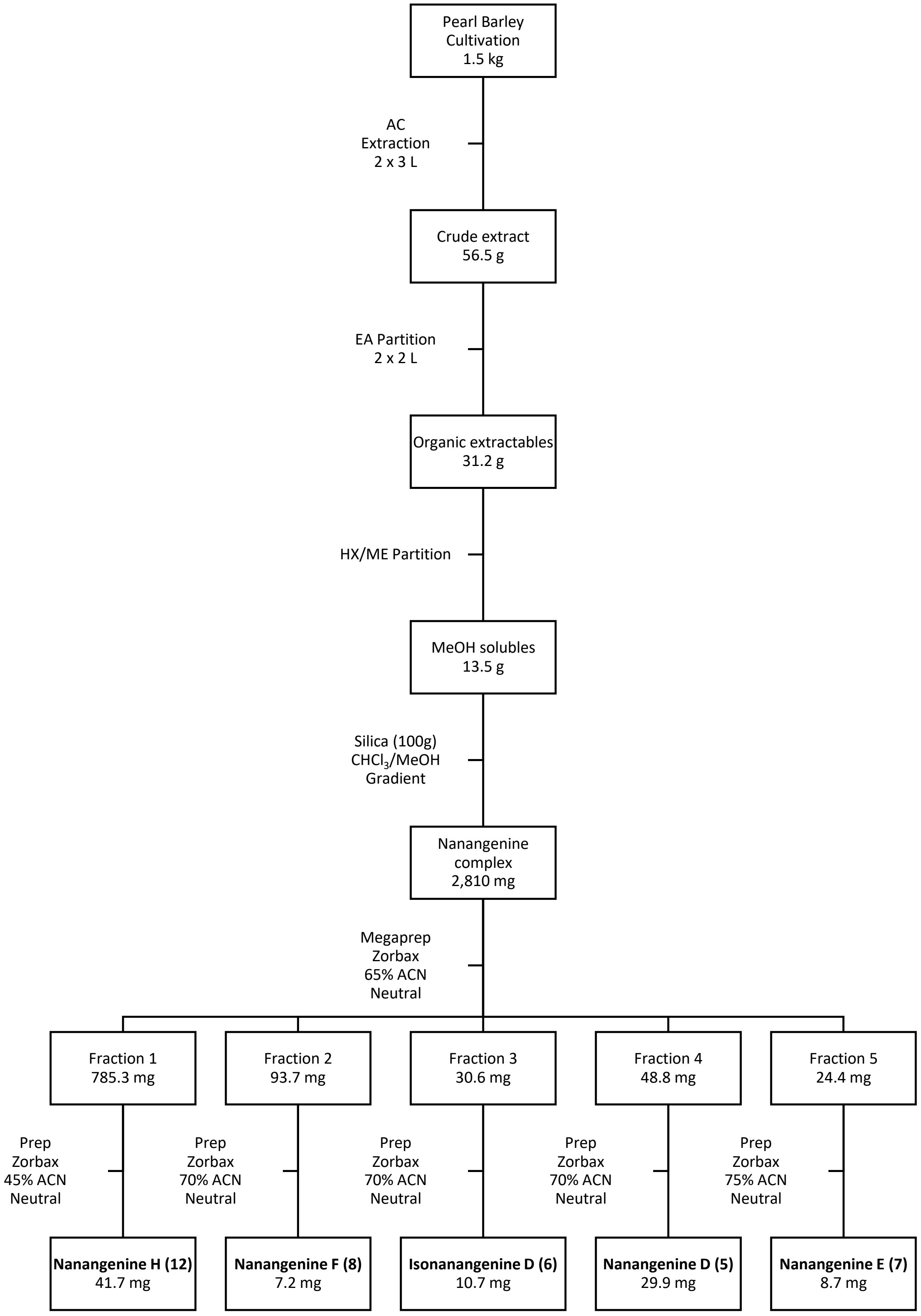

Figure S2. Fractionation scheme for culture B (pearl barley)

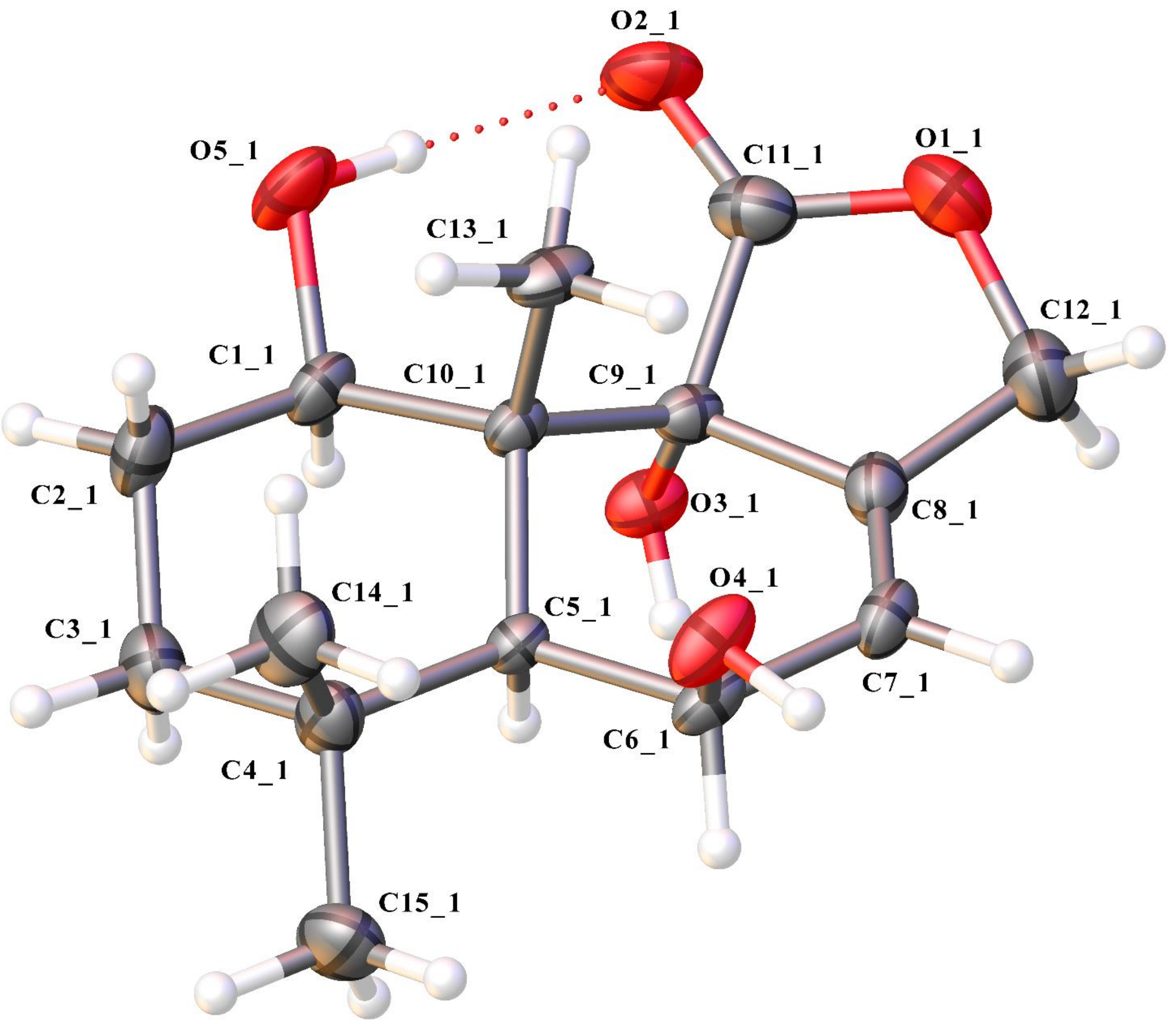

Figure S3. Olex2 depiction of one of the four crystallographically-independent molecules of nanangenine A (**1**), with 50% displacement ellipsoids and showing the intramolecular hydrogen bond.

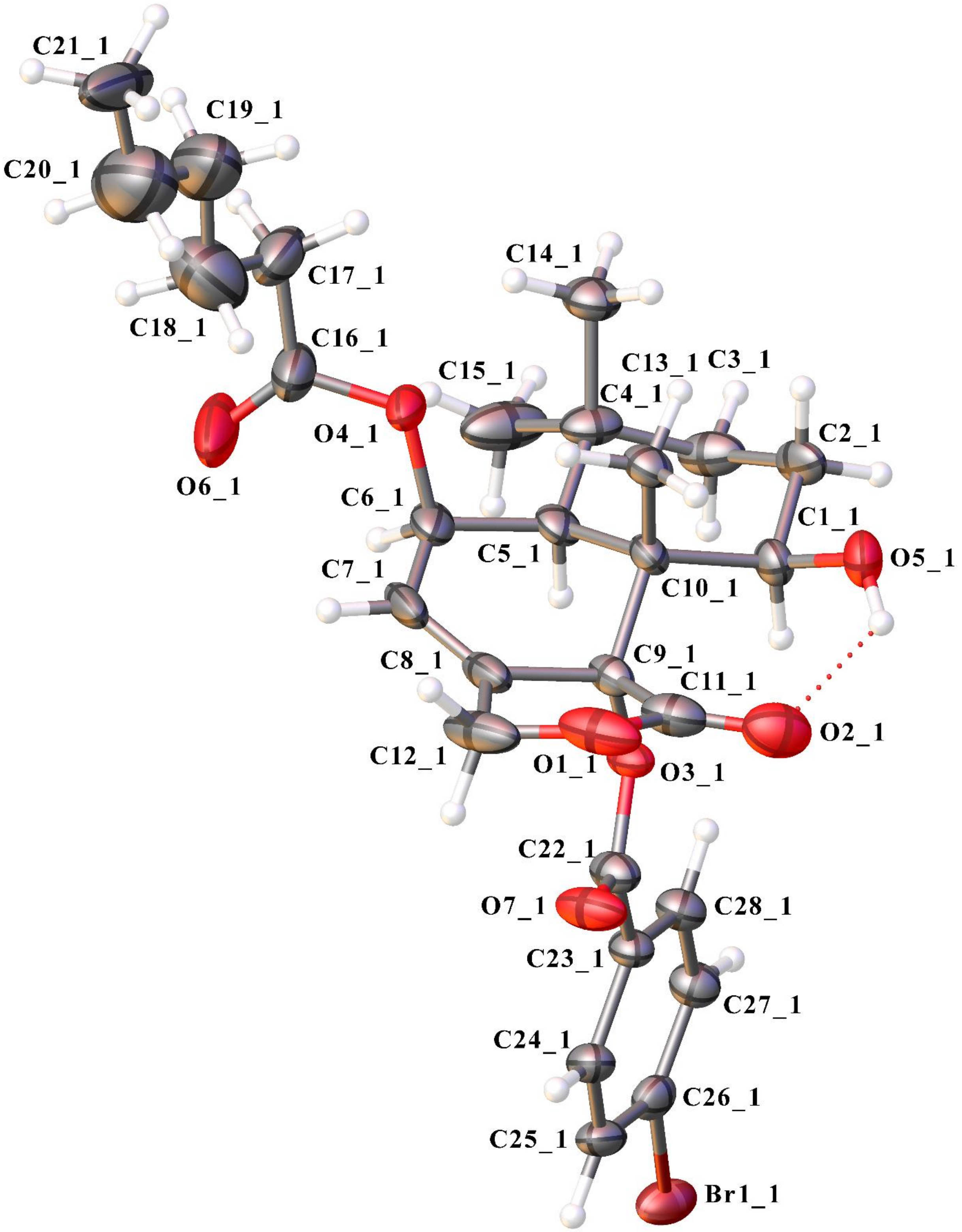

Figure S4. Olex2 depiction of one of the two crystallographically-independent molecules of 9-*O*-(4-bromobenzoyl)-nanangenine B (**2b**) with 50% displacement ellipsoids.

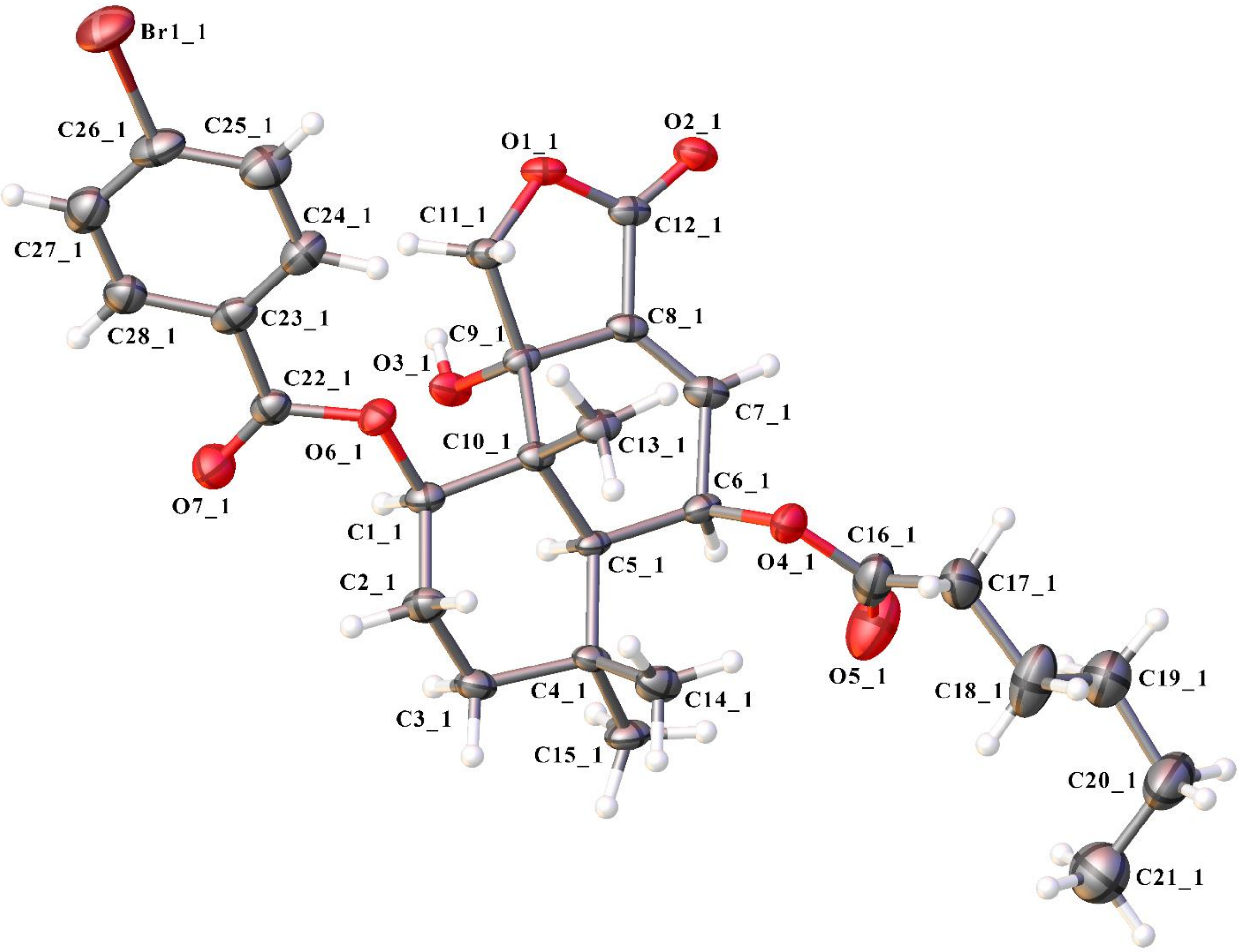

Figure S5. Olex2 depiction of one of the two crystallographically-independent molecules of 1-*O*-(4-bromobenzoyl)-isonanangenine B (**3b**) with 75% displacement ellipsoids.

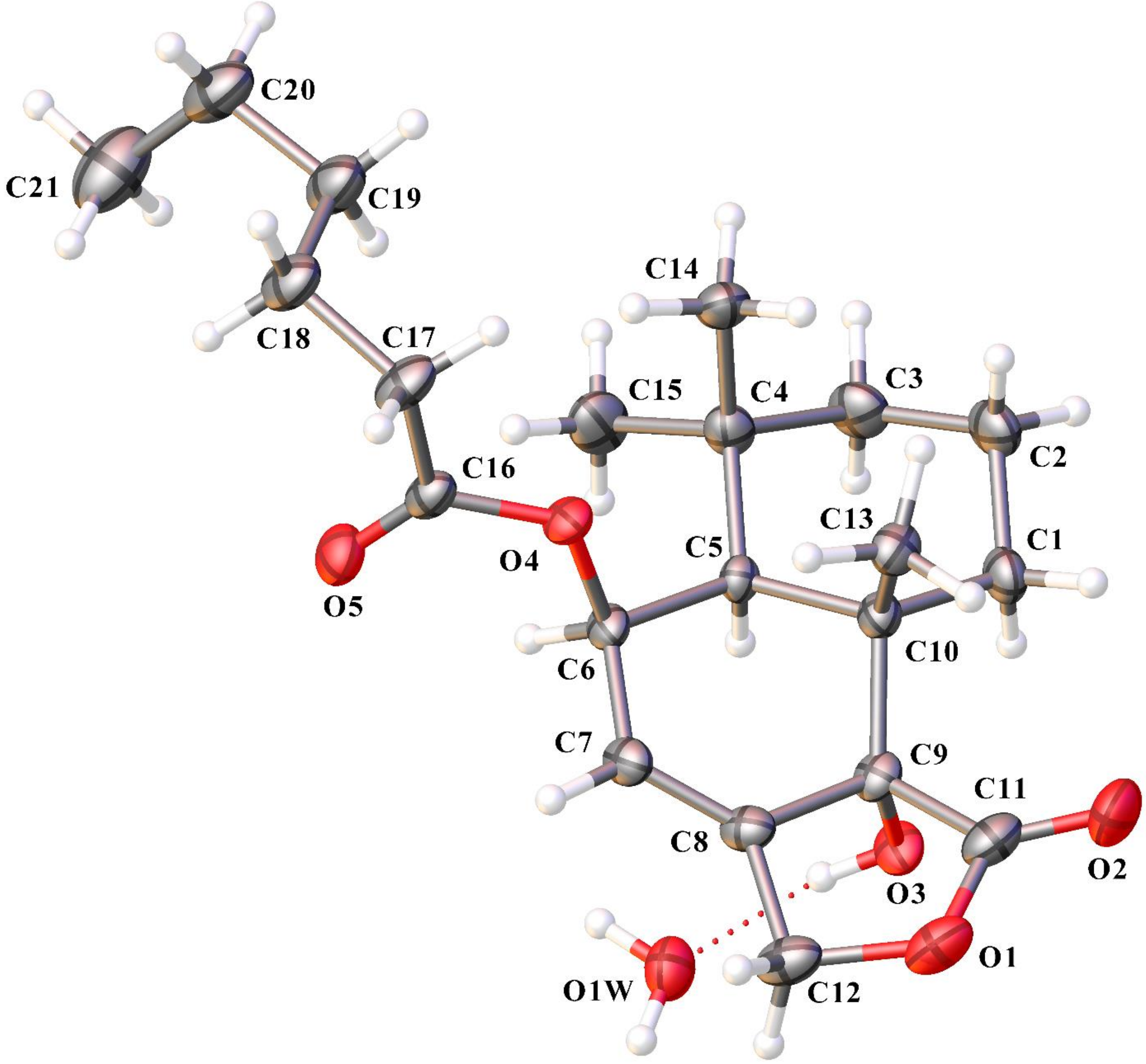

Figure S6. Olex2 depiction of nanangenine C (**4**) and a hydrogen bond-linked water molecule, with 50% displacement ellipsoids.

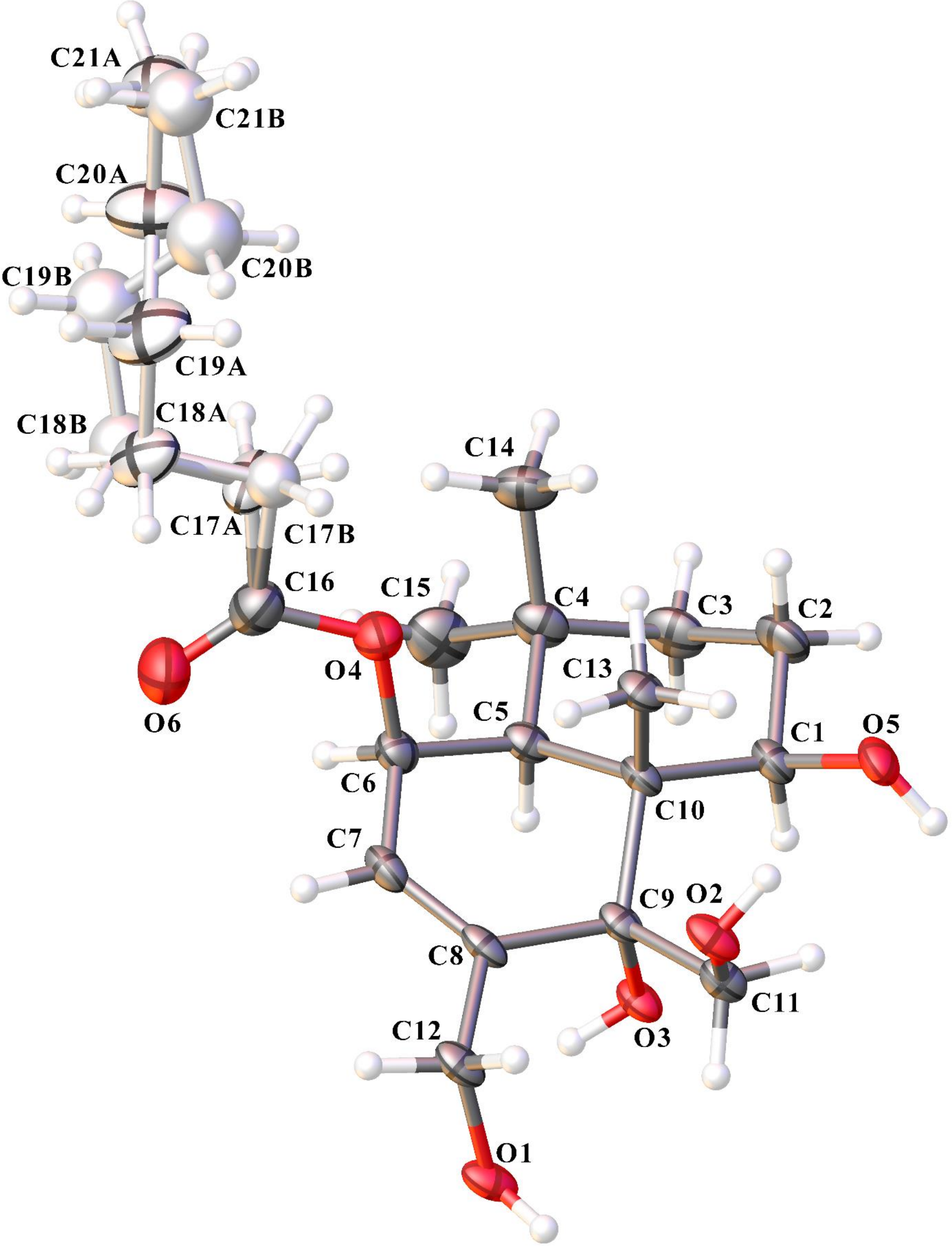

Figure S7. Olex2 depiction of nanangenine G (**9**) with 50% displacement ellipsoids and disordered sites shown with 'faded' colour.

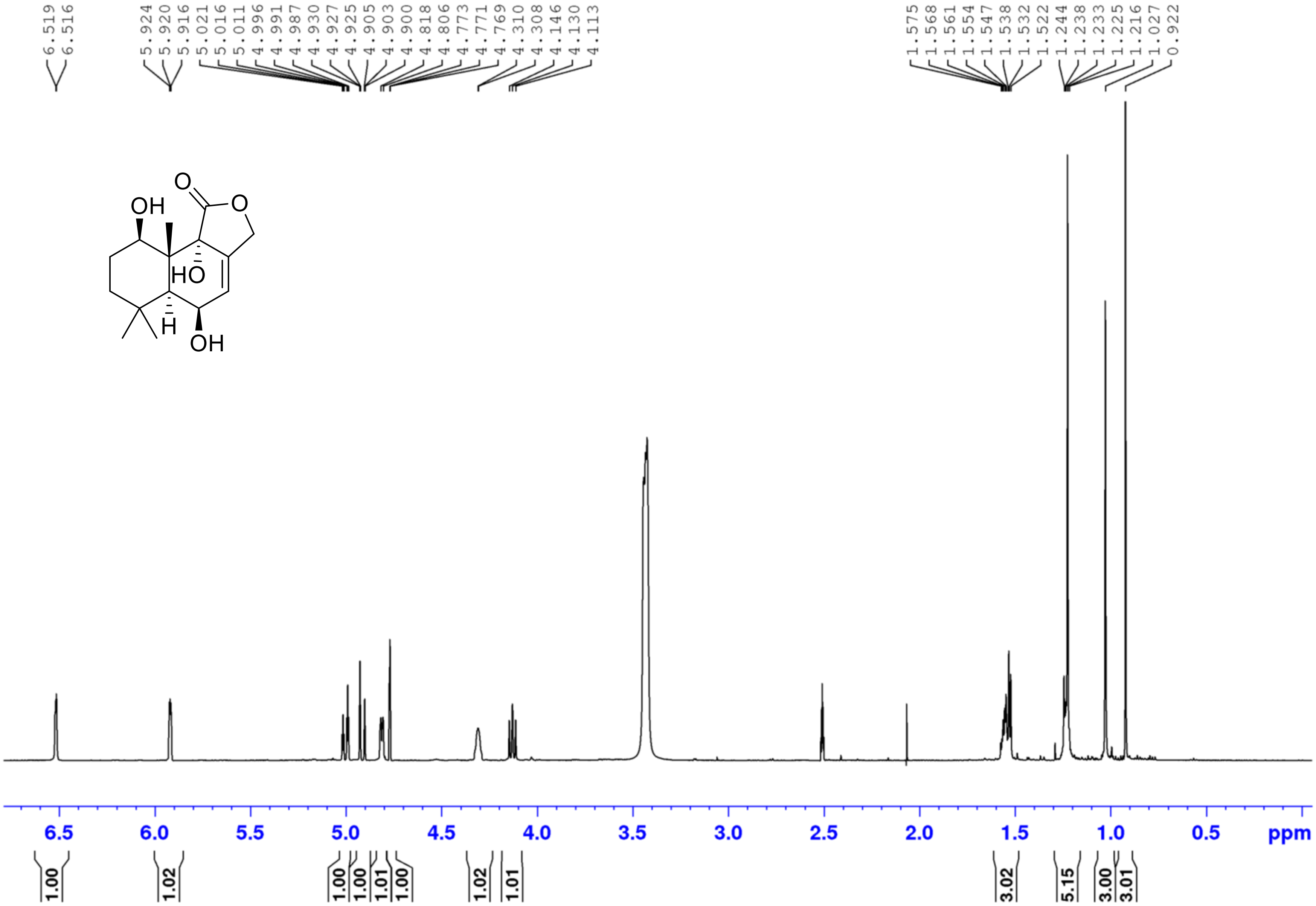

Figure S8. ^{1}H NMR spectrum (500 MHz, DMSO-d_6) of nanangenine A (**1**).

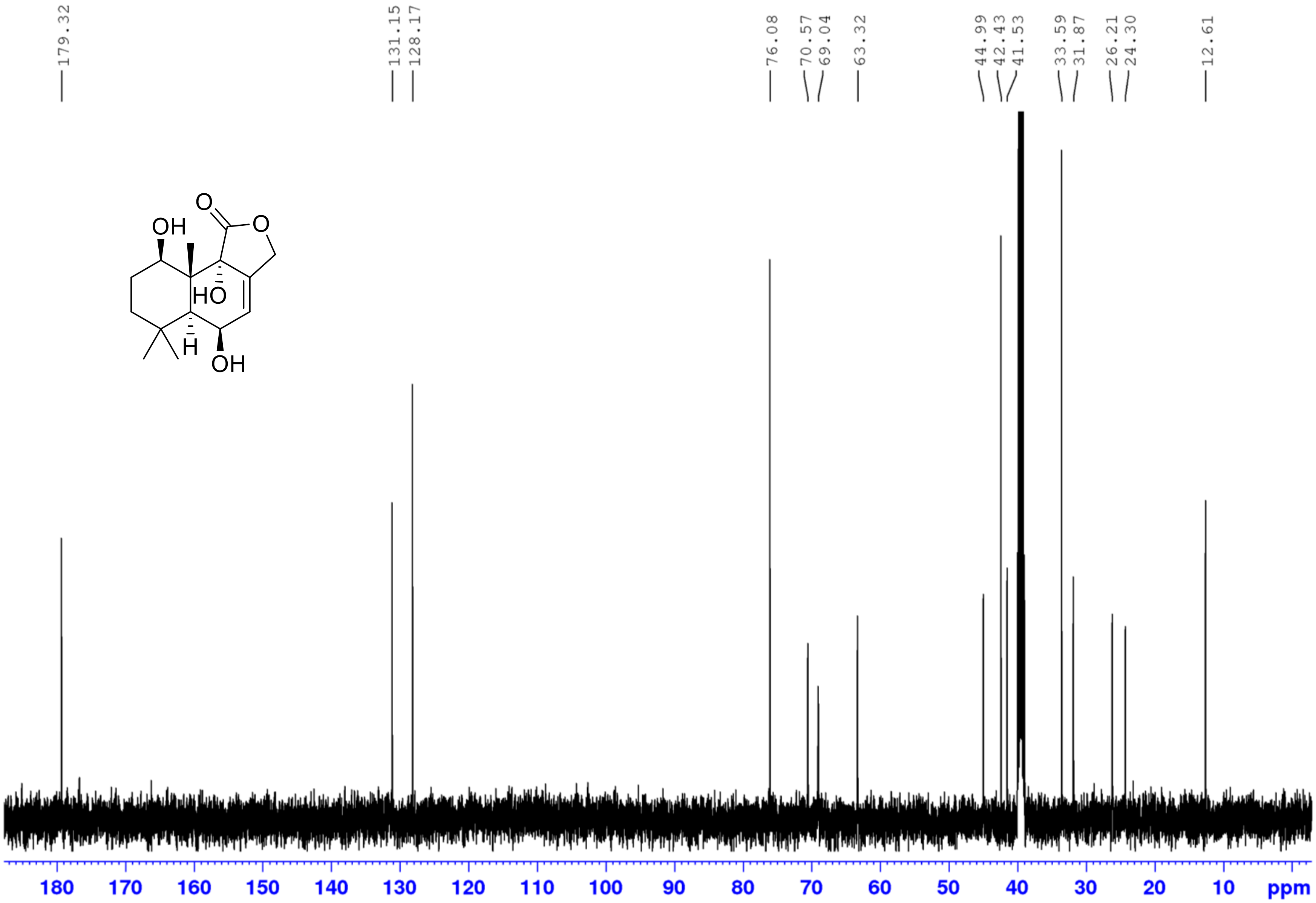

Figure S9. ^{13}C NMR spectrum (150 MHz, DMSO-d_6) of nanangenine A (**1**).

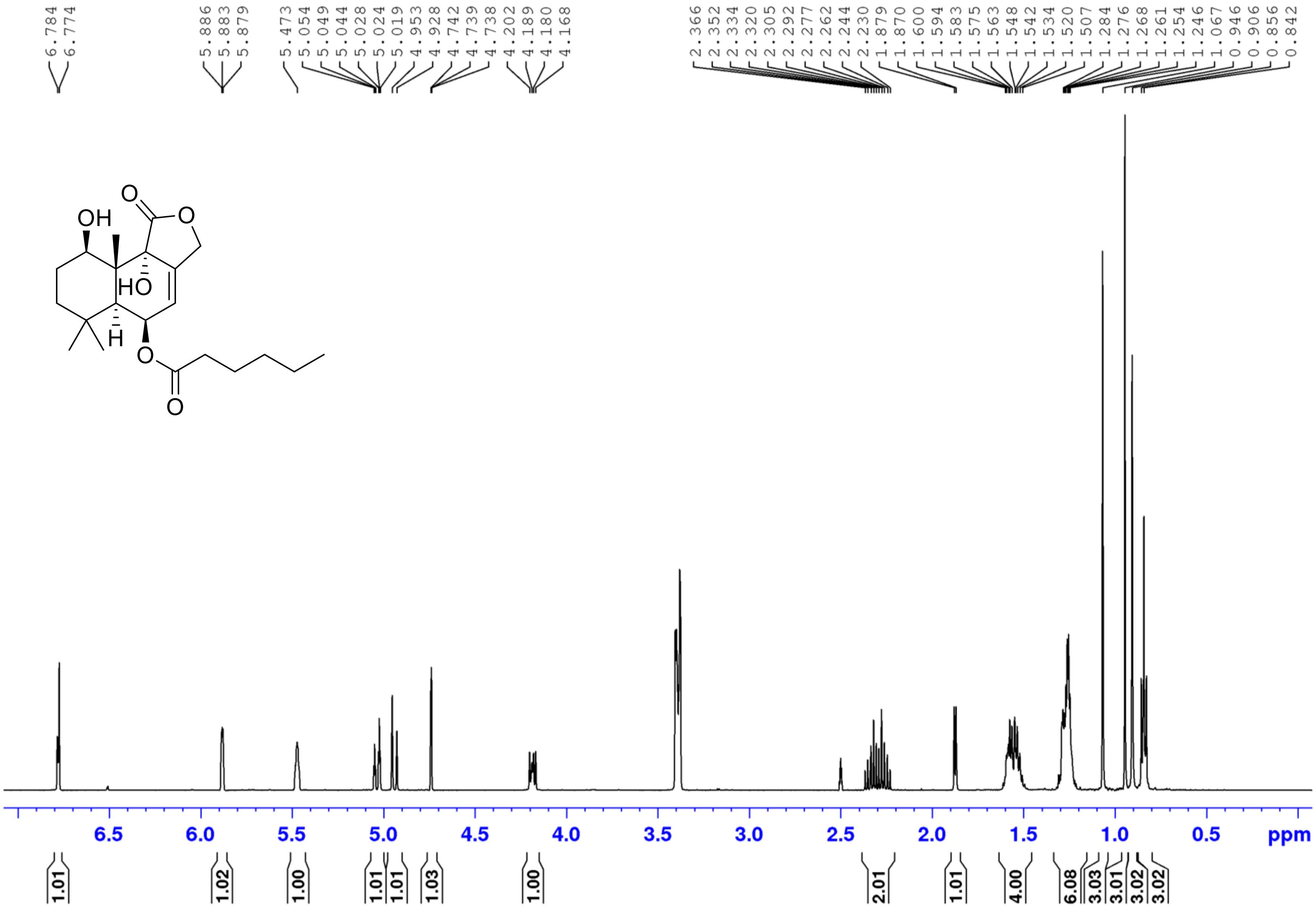

Figure S10. ^{1}H NMR spectrum (500 MHz, DMSO-d_6) of nanangenine B (**2**).

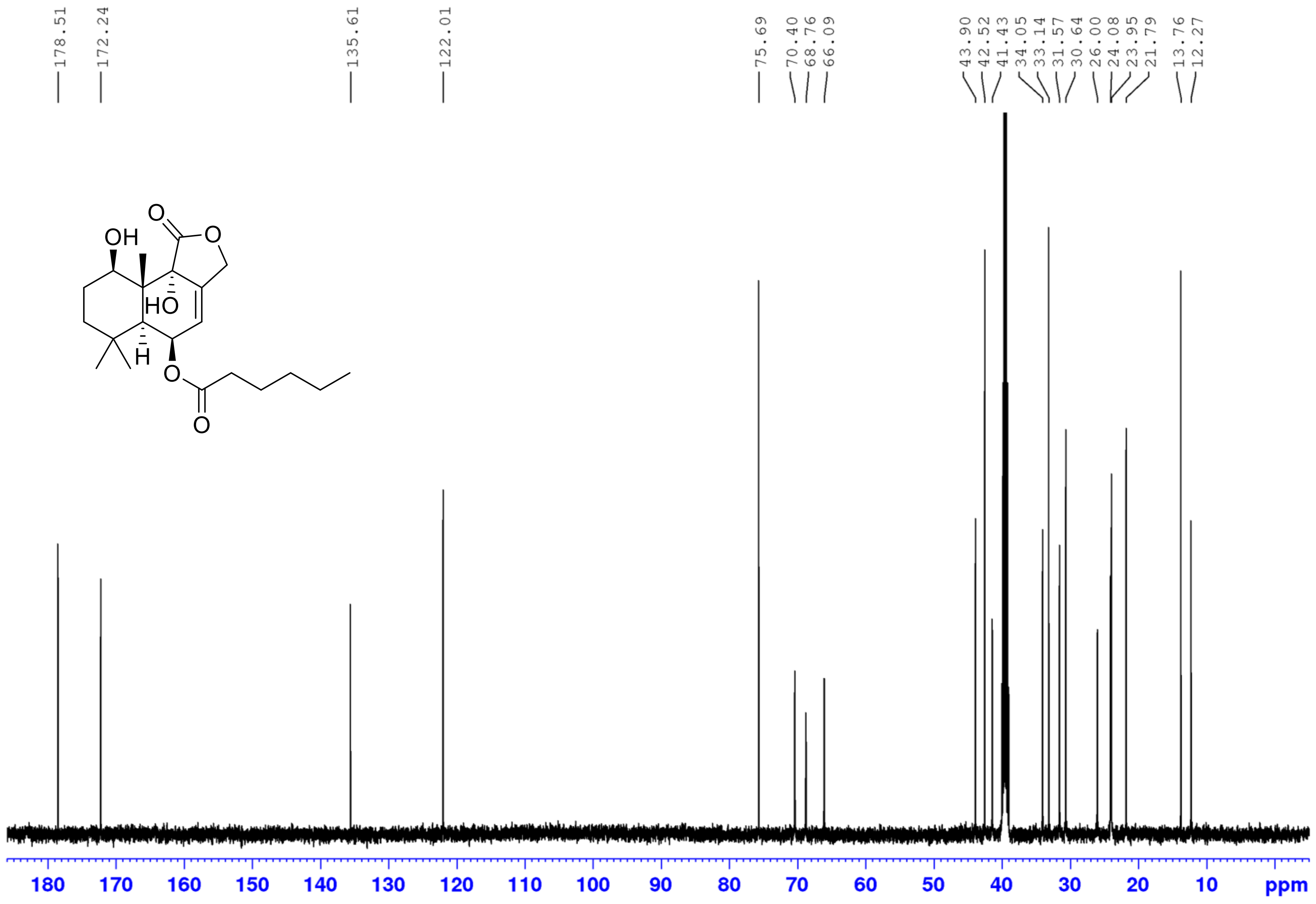

Figure S11. ^{13}C NMR spectrum (125 MHz, DMSO-d_6) of nanangenine B (**2**).

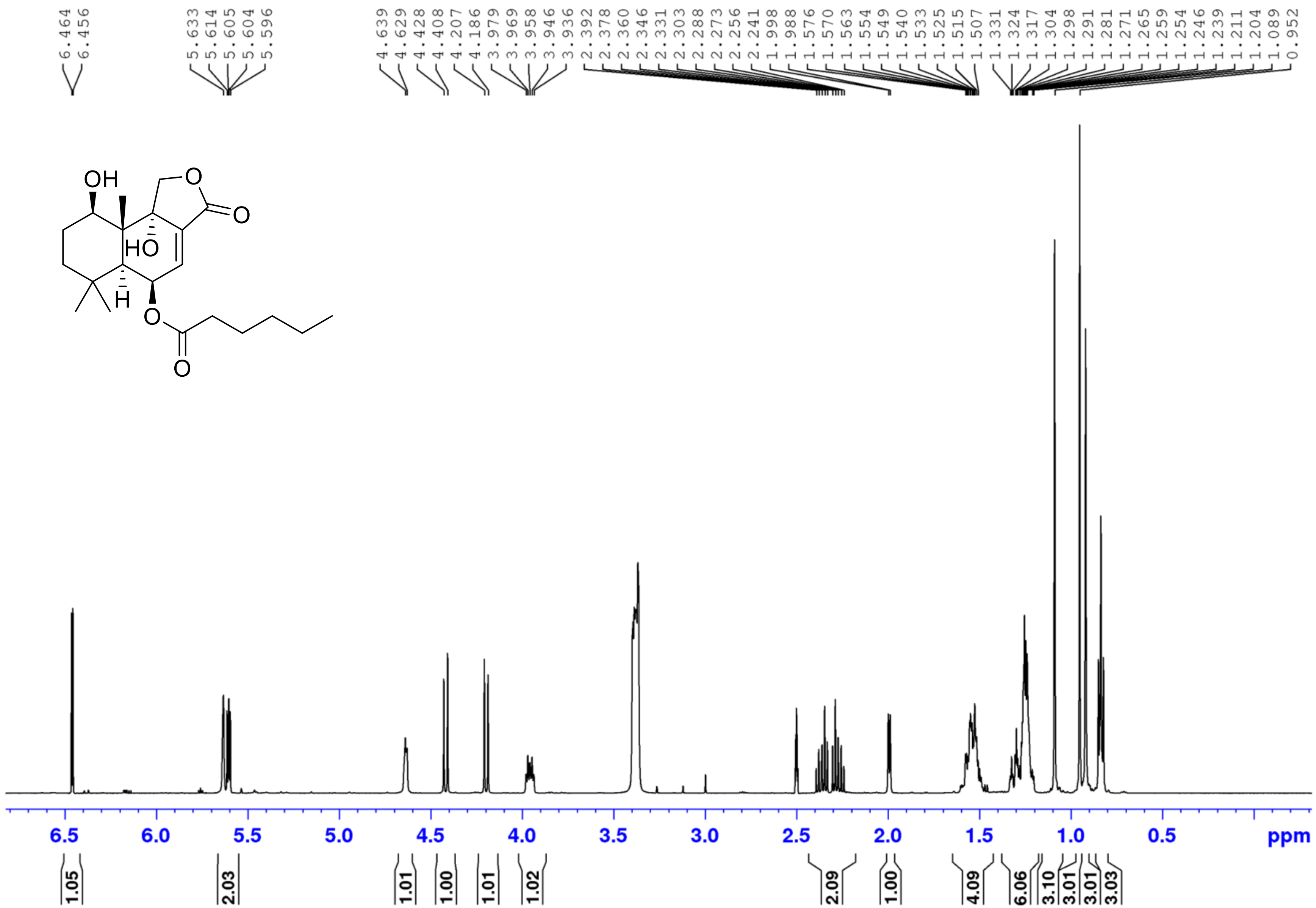

Figure S12. ^{1}H NMR spectrum (500 MHz, DMSO-d_6) of isonanangenine B (**3**).

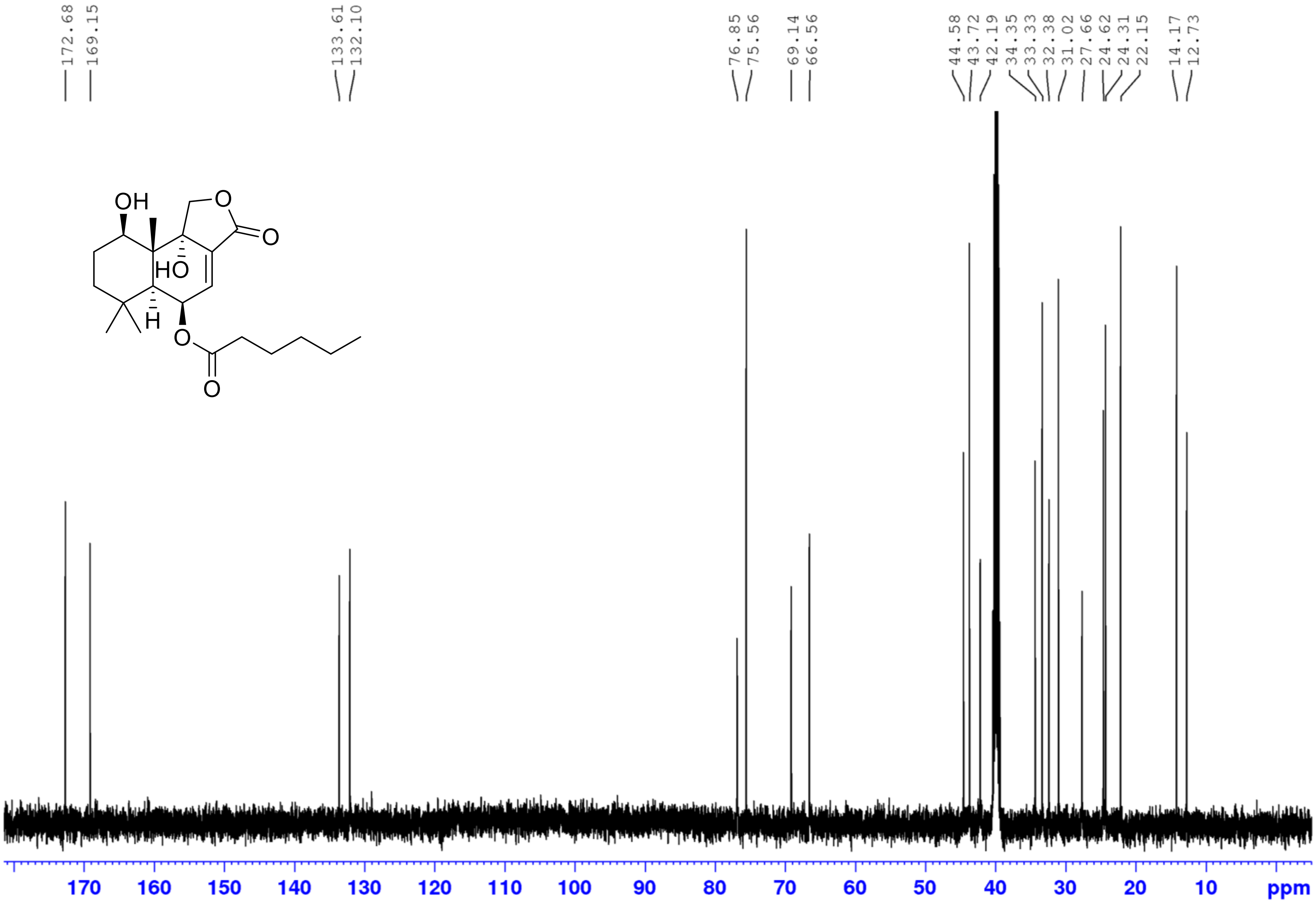

Figure S13. ^{13}C NMR spectrum (125 MHz, DMSO-d_6) of isonanangenine B (**3**).

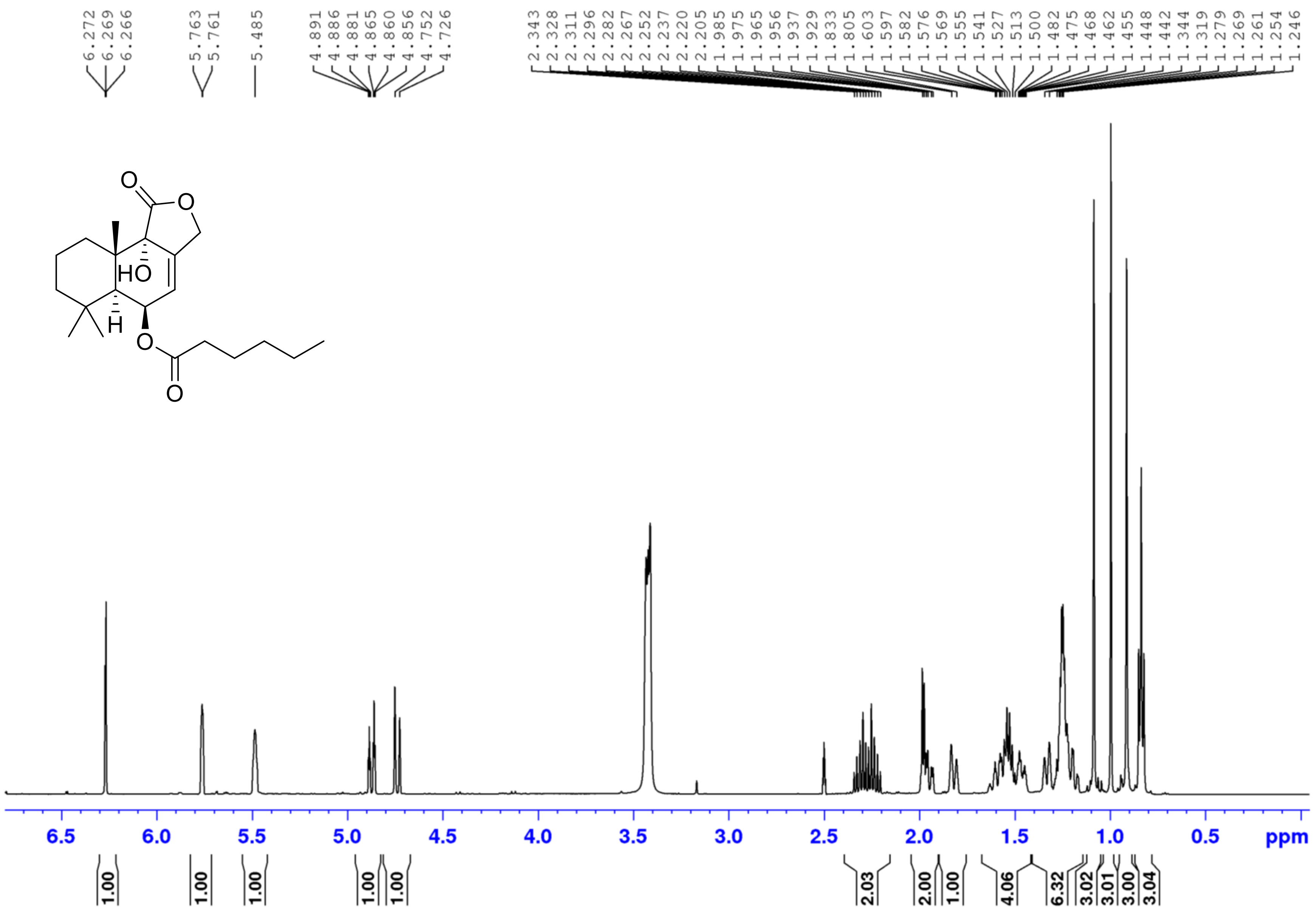

Figure S14. ^{1}H NMR spectrum (500 MHz, DMSO-d_6) of nanangenine C (**4**).

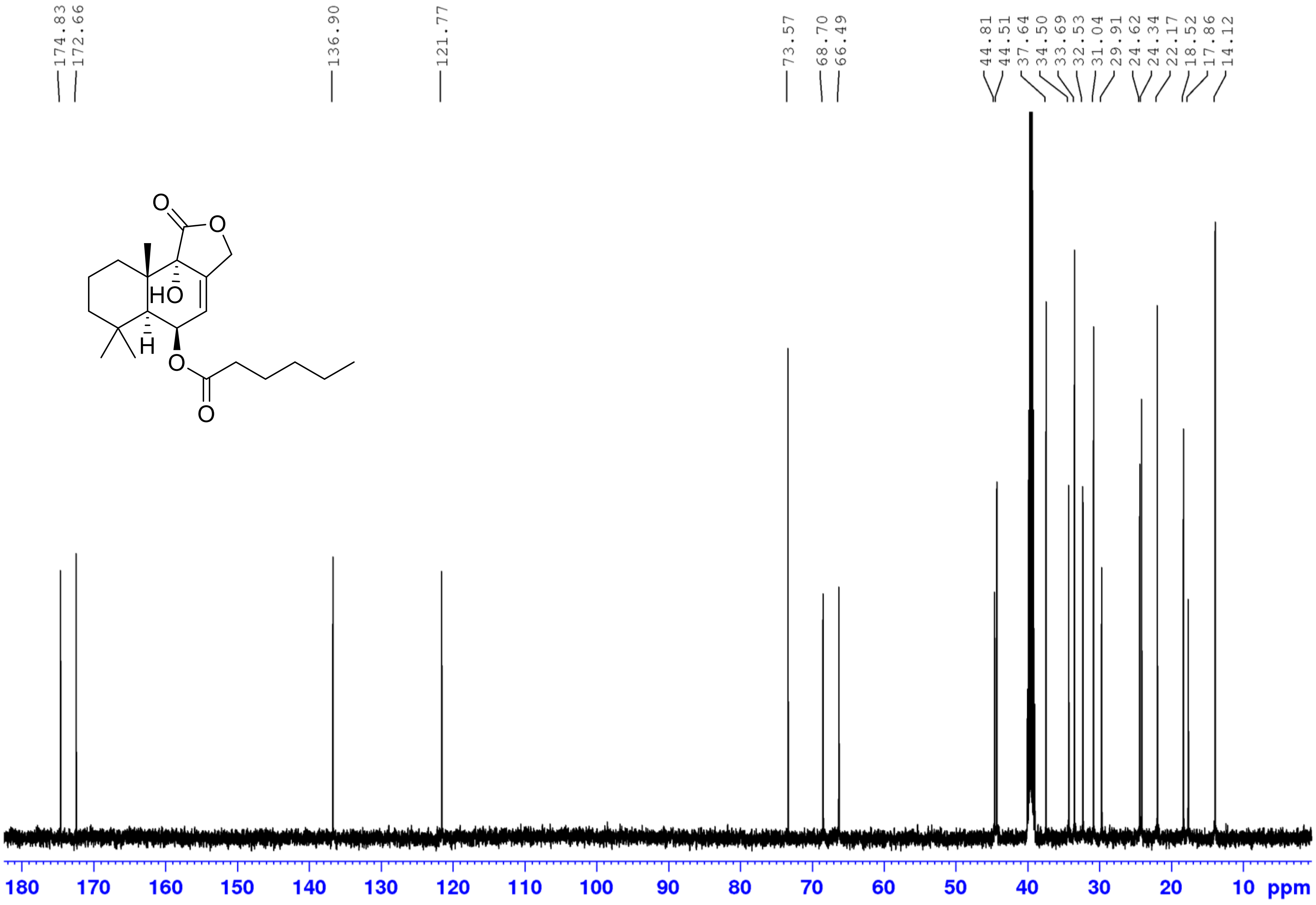

Figure S15. ^{13}C NMR spectrum (125 MHz, DMSO-d_6) of nanangenine C (**4**).

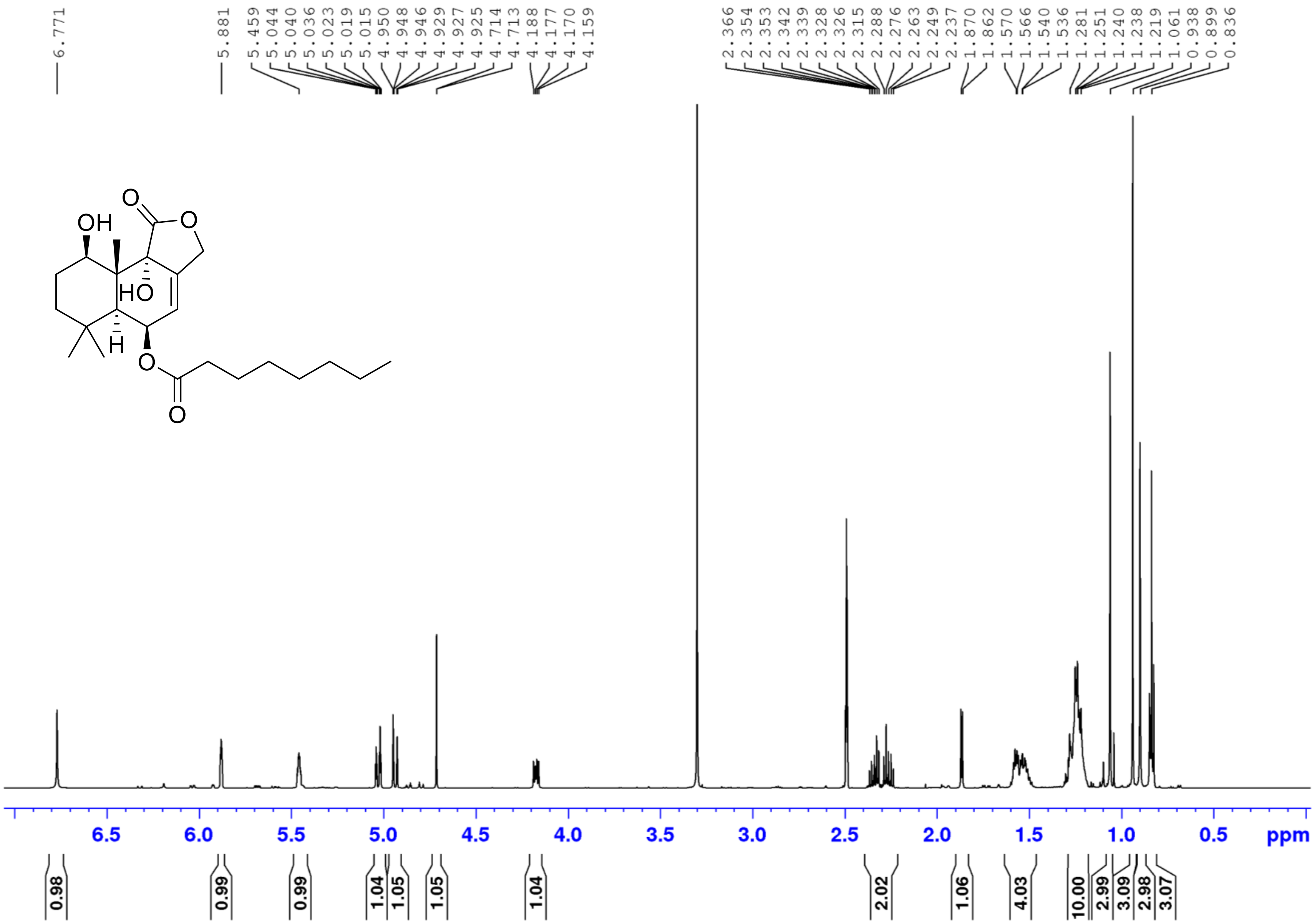

Figure S16. ^{1}H NMR spectrum (600 MHz, DMSO-d_6) of nanangenine D (**5**).

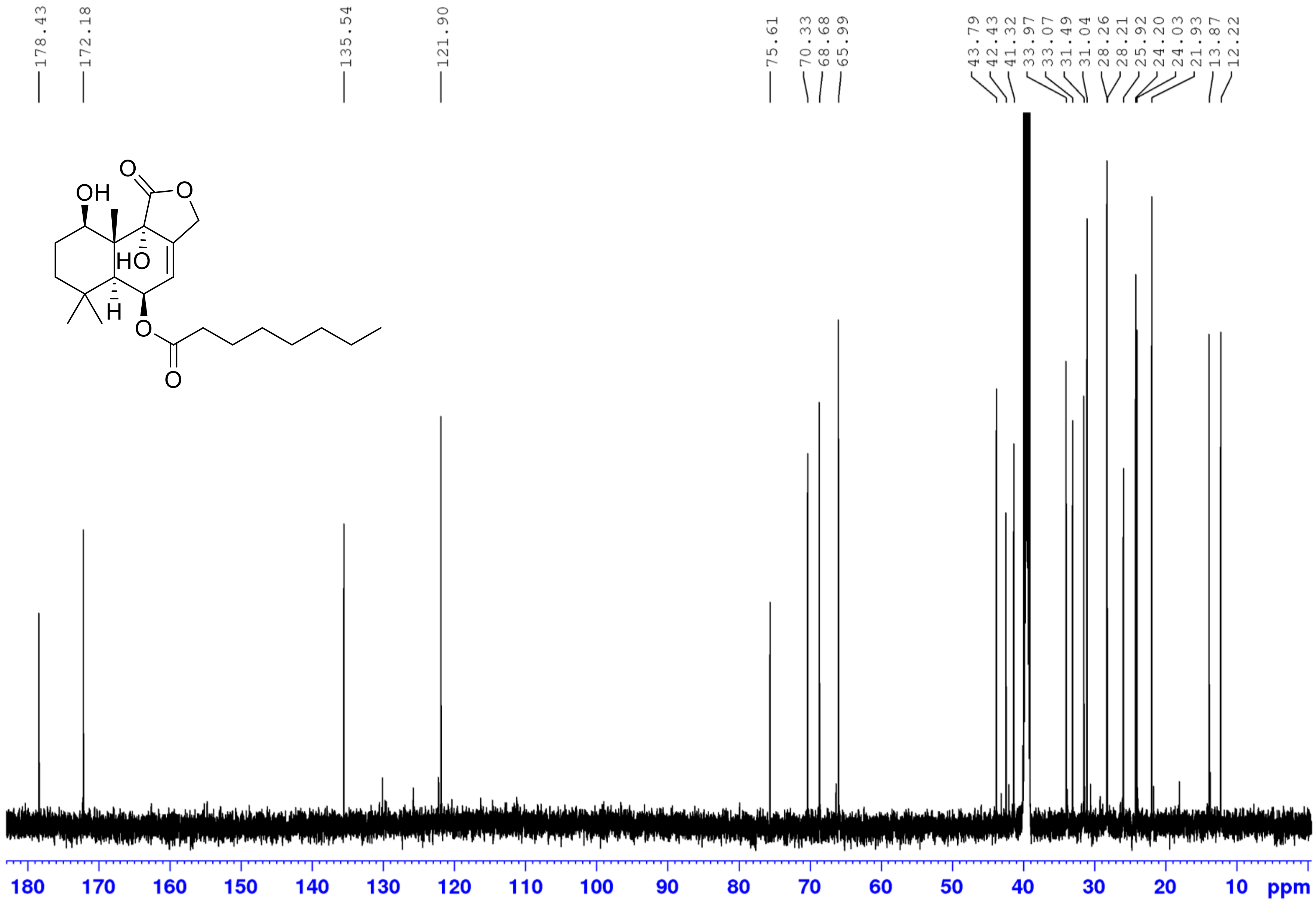

Figure S17. ^{13}C NMR spectrum (150 MHz, DMSO-d_6) of nanangenine D (**5**).

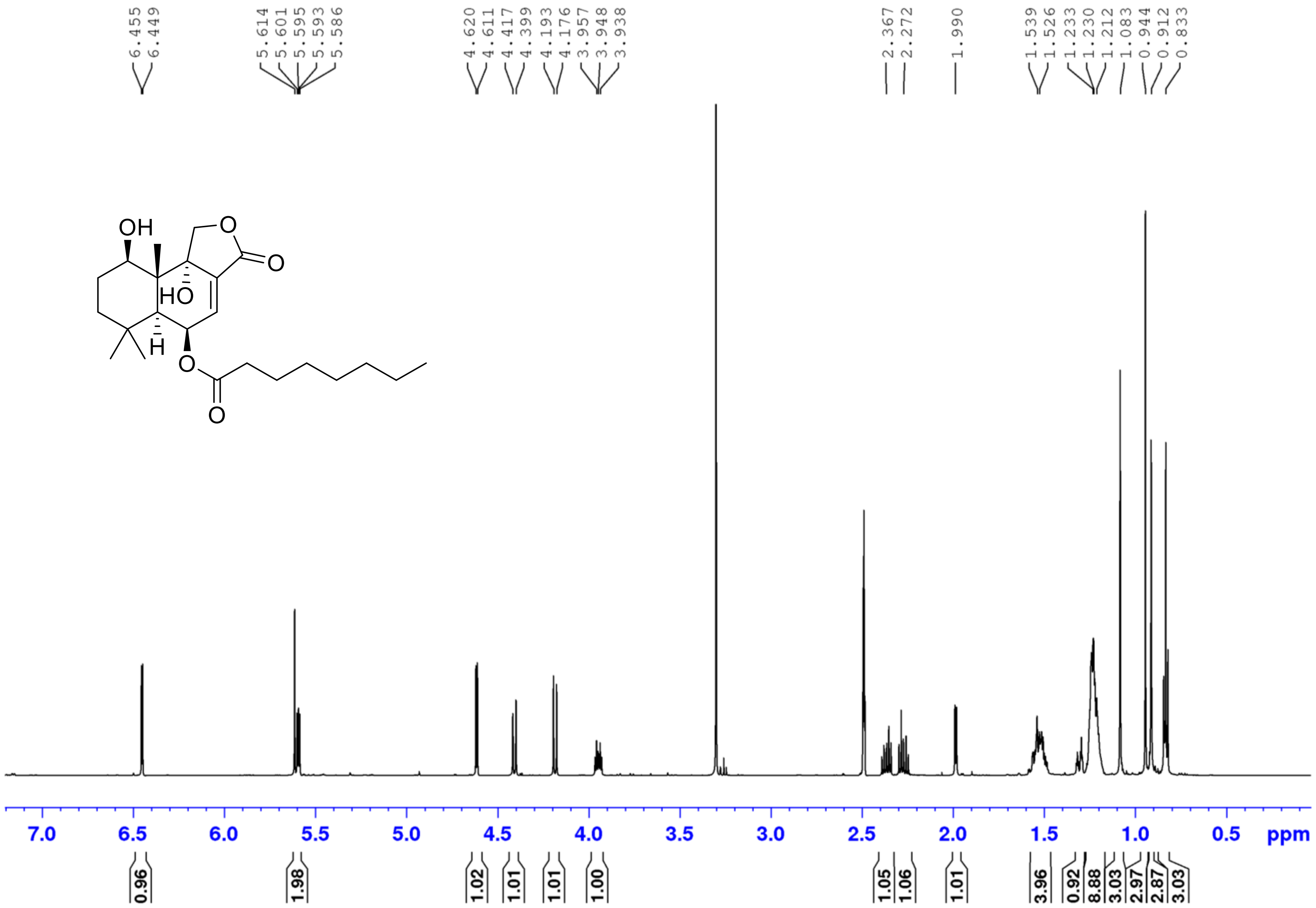

Figure S18. ^{1}H NMR spectrum (600 MHz, DMSO-d_6) of isonanangenine D (**6**).

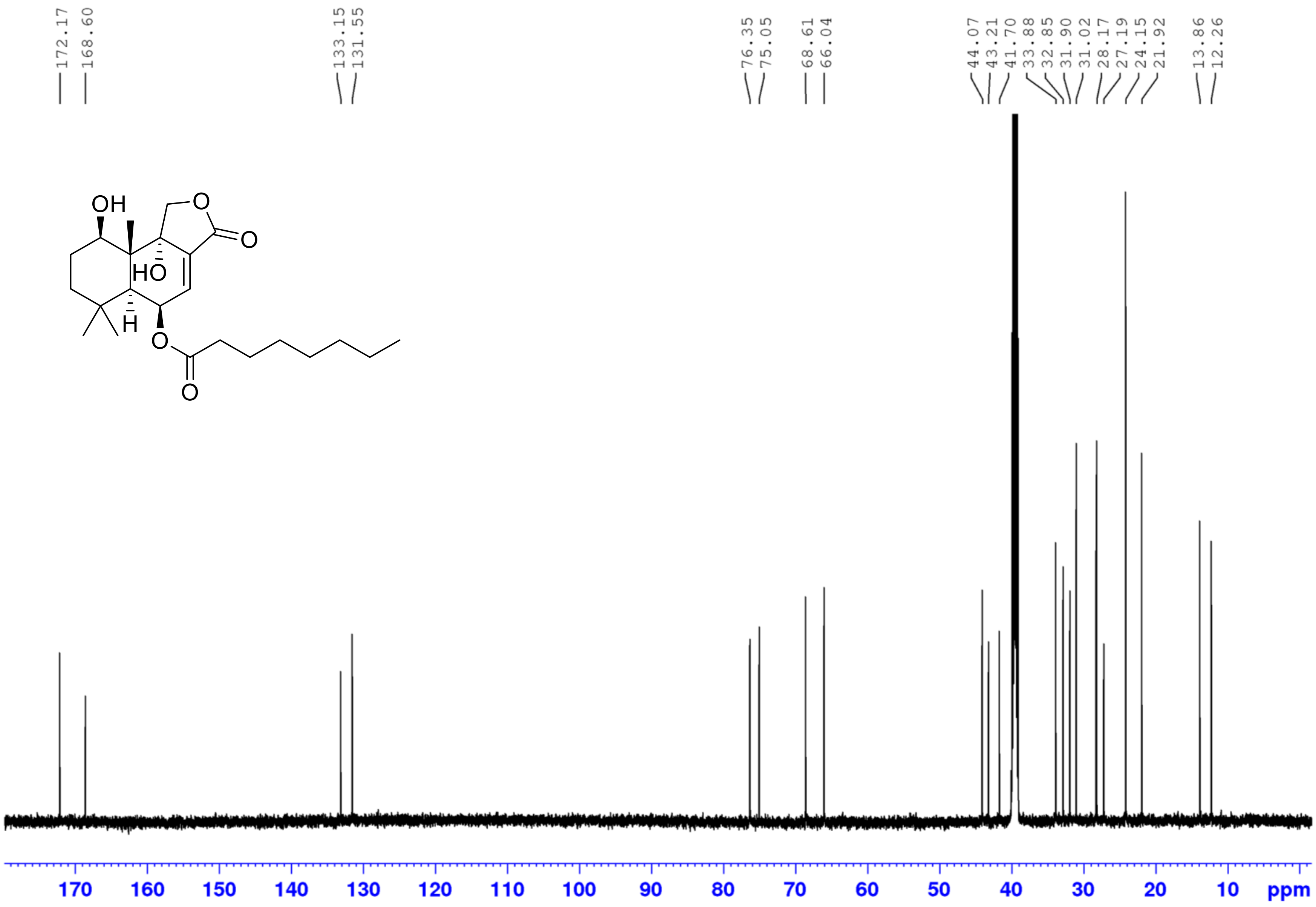

Figure S19. ^{13}C NMR spectrum (150 MHz, DMSO-d_6) of isonanangenine D (**6**).

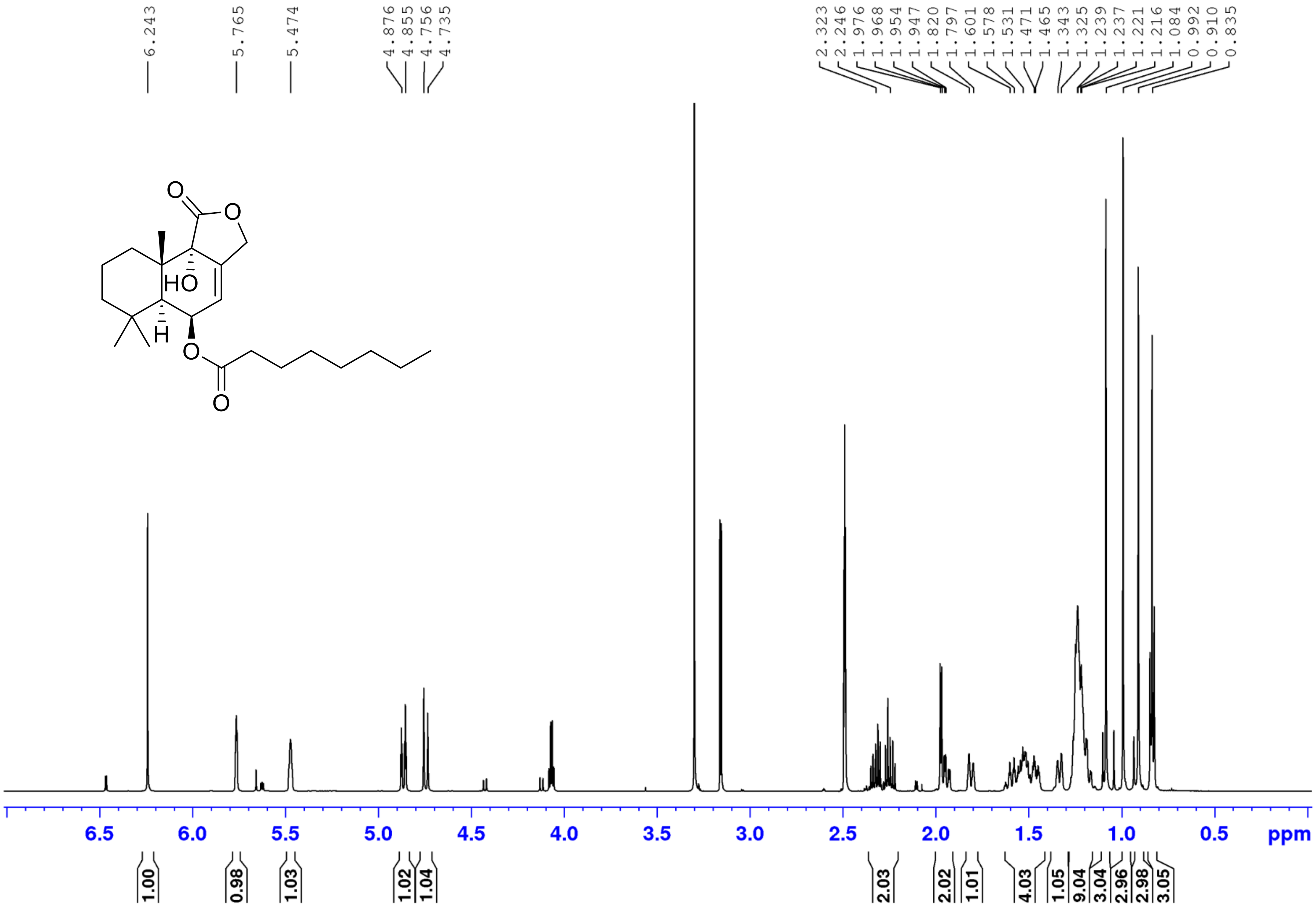

Figure S20. ^{1}H NMR spectrum (600 MHz, DMSO-d_6) of nanangenine E (**7**).

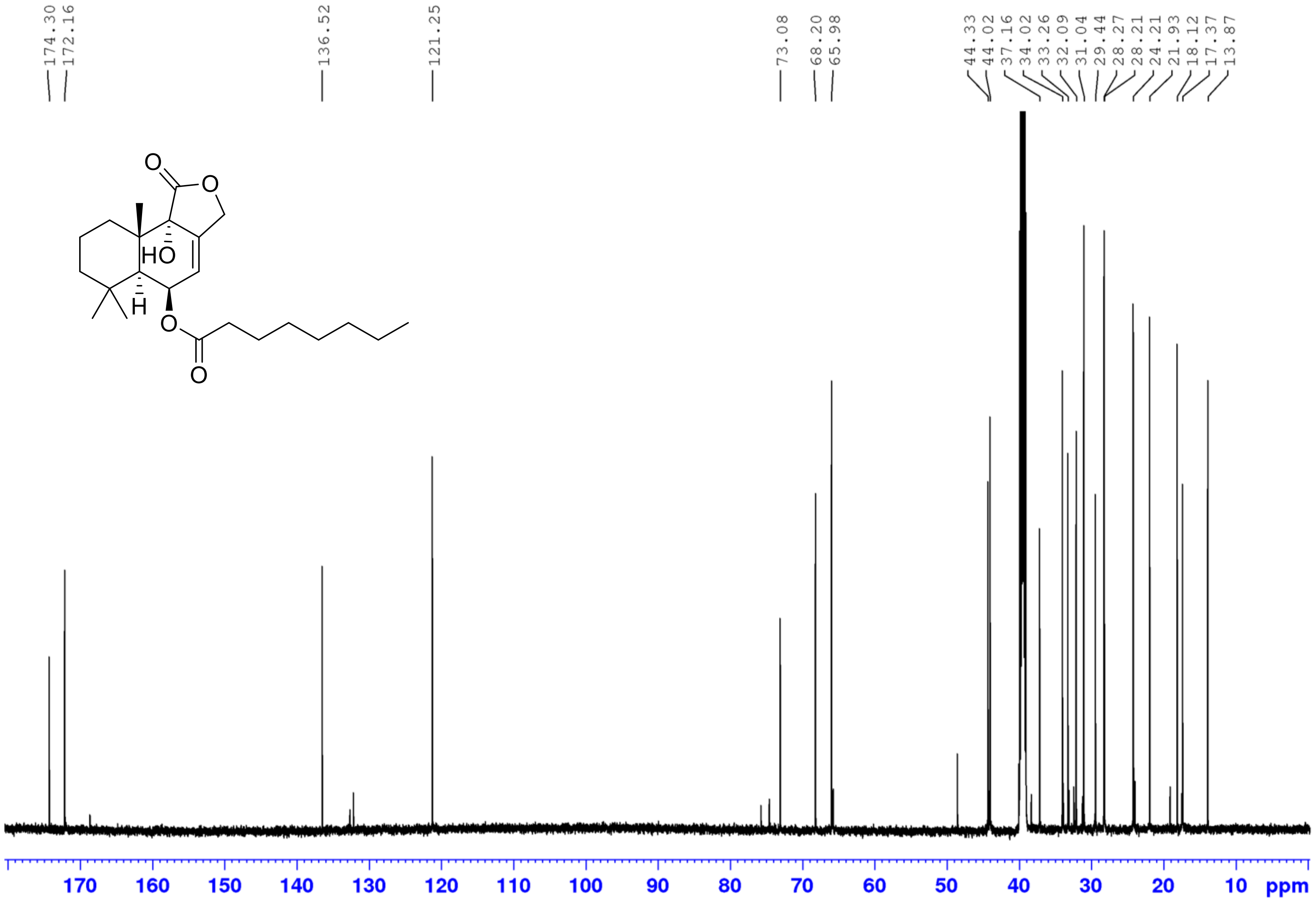

Figure S21. ^{13}C NMR spectrum (150 MHz, DMSO-d_6) of nanangenine E (**7**).

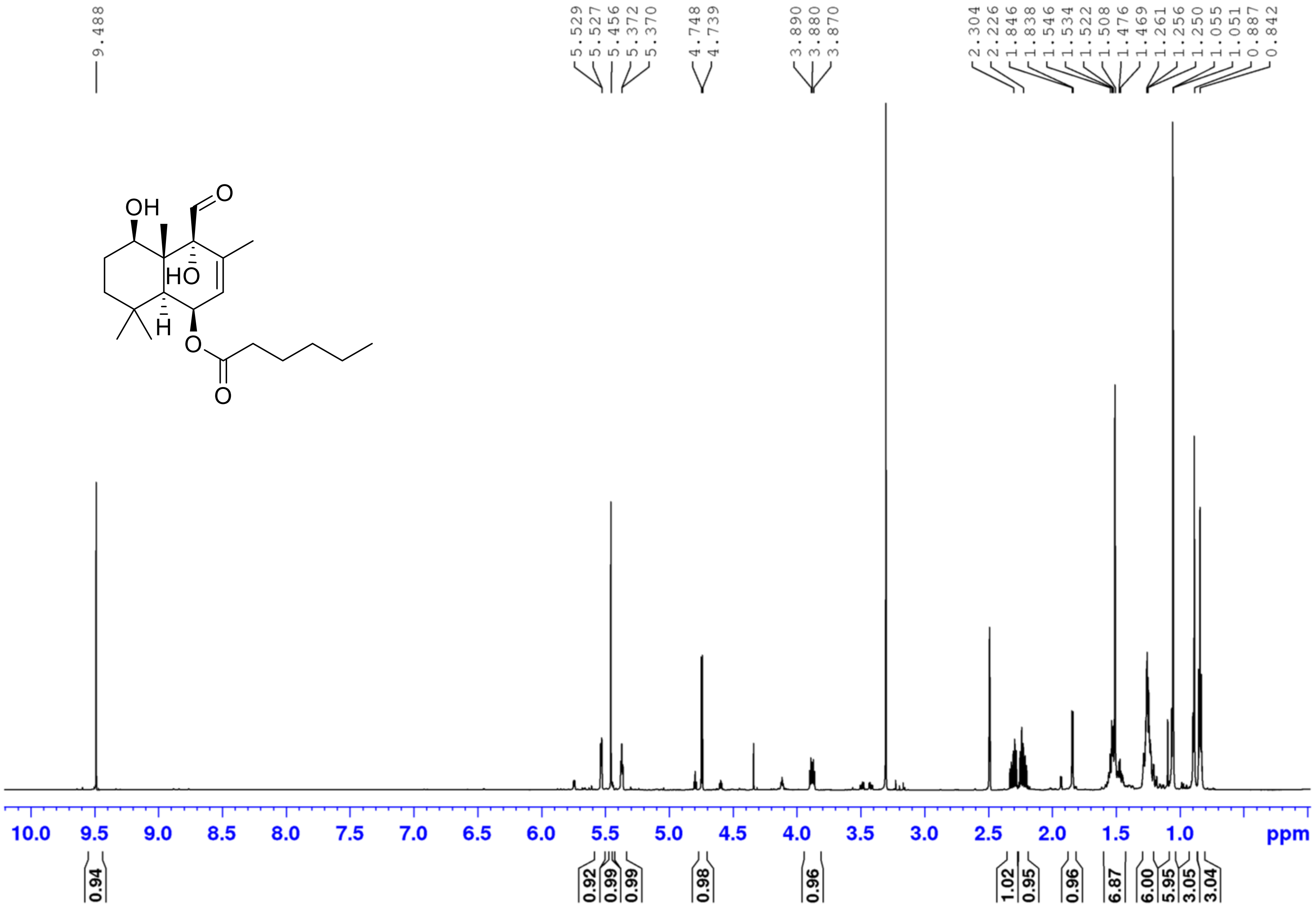

Figure S22. ^{1}H NMR spectrum (600 MHz, DMSO-d_6) of nanangenine F (**8**).

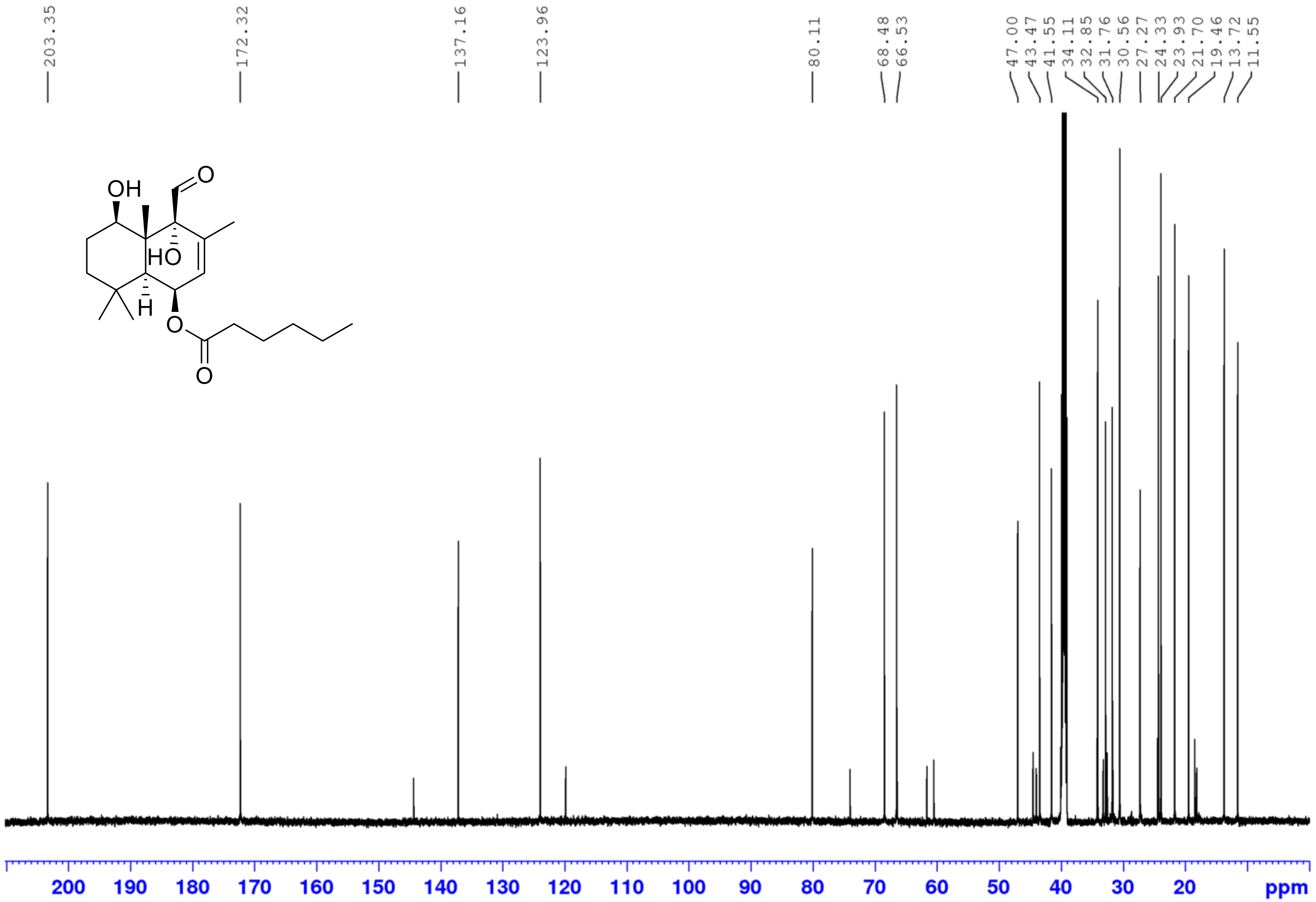

Figure S23. ^{13}C NMR spectrum (150 MHz, DMSO-d_6) of nanangenine F (**8**).

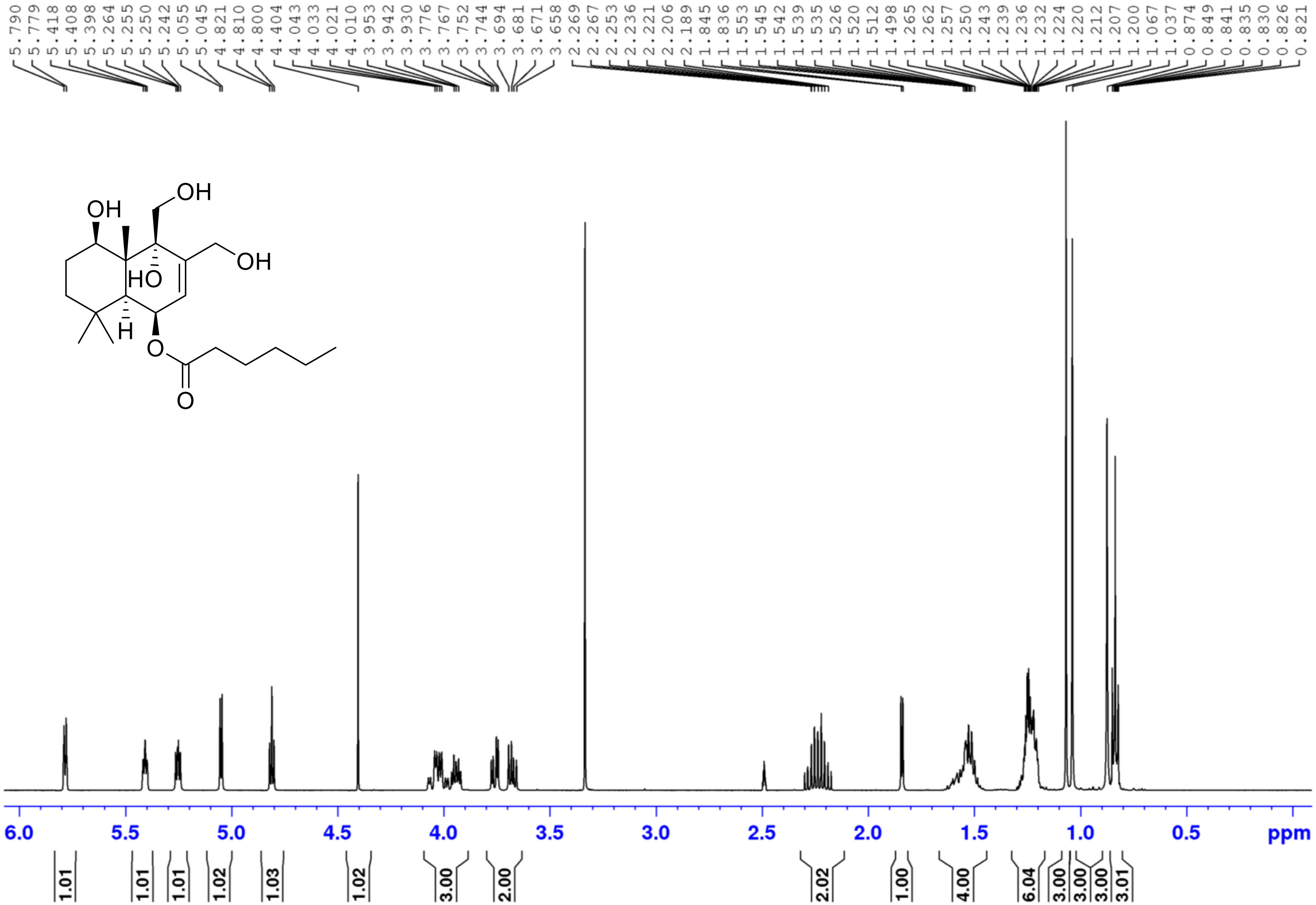

Figure S24. ^{1}H NMR spectrum (500 MHz, DMSO-d_6) of nanangenine G (**9**).

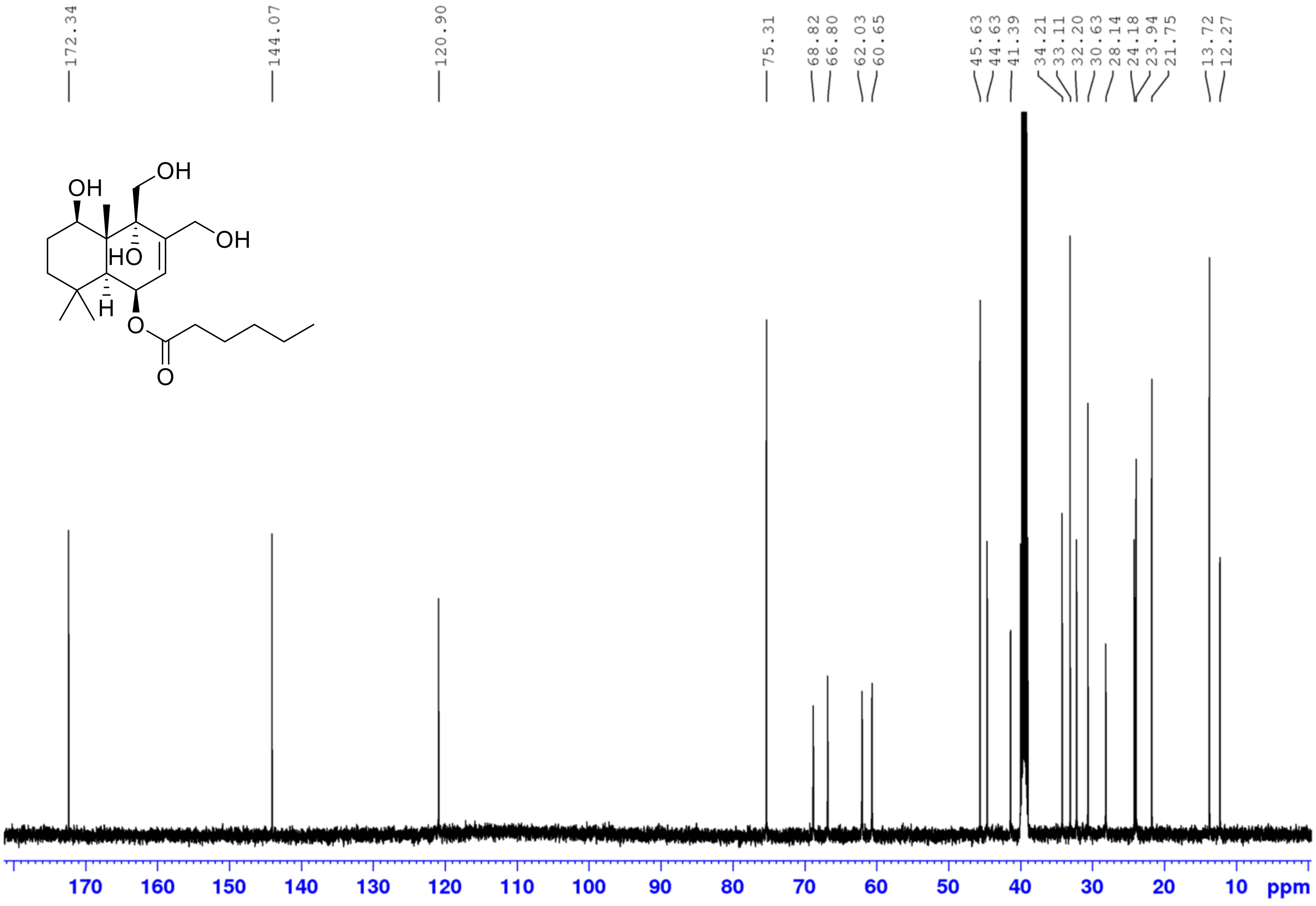

Figure S25. ^{13}C NMR spectrum (125 MHz, DMSO-d_6) of nanangenine G (**9**).

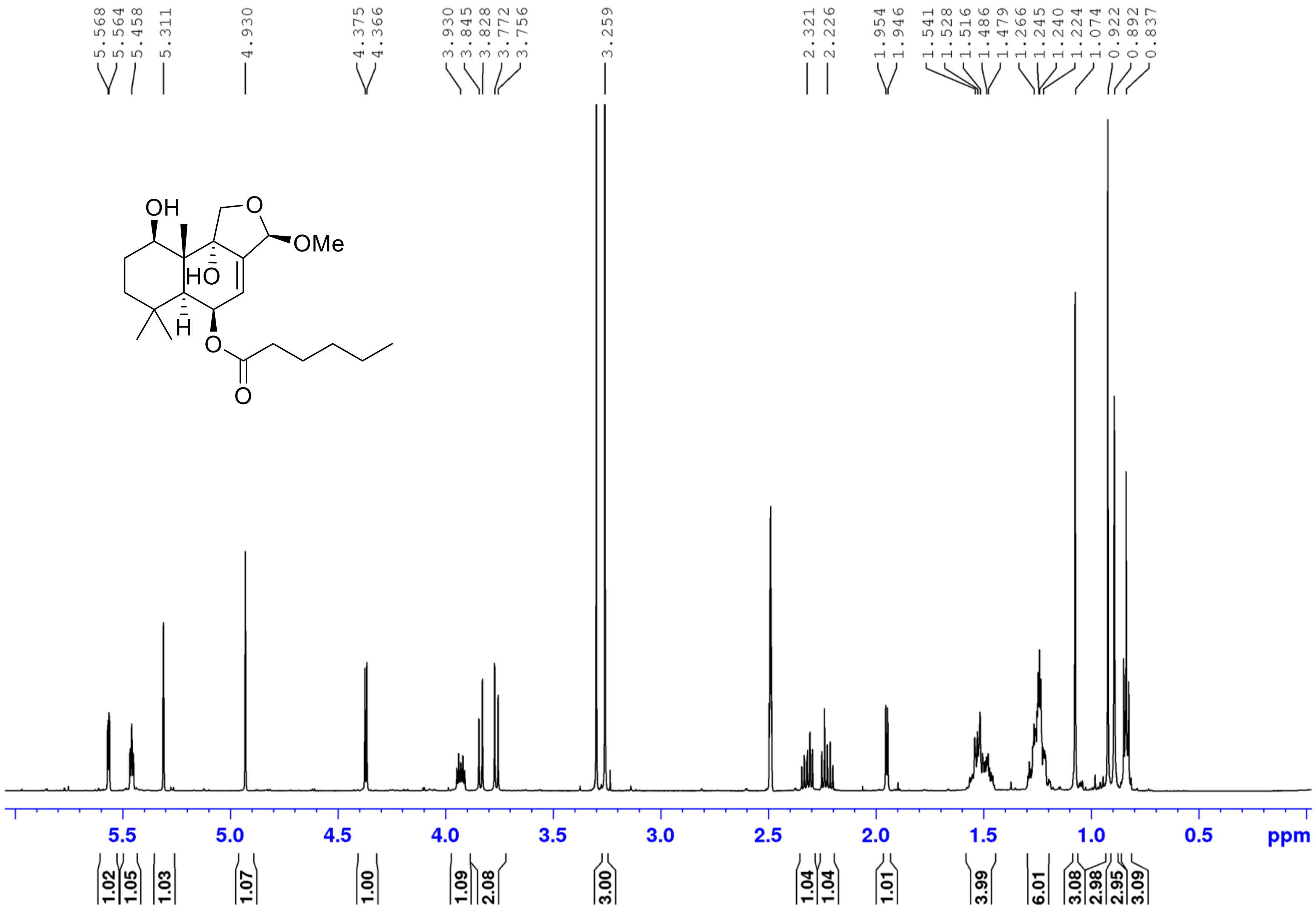

Figure S26. ^{1}H NMR spectrum (600 MHz, DMSO-d_6) of nanangenine H (**10**).

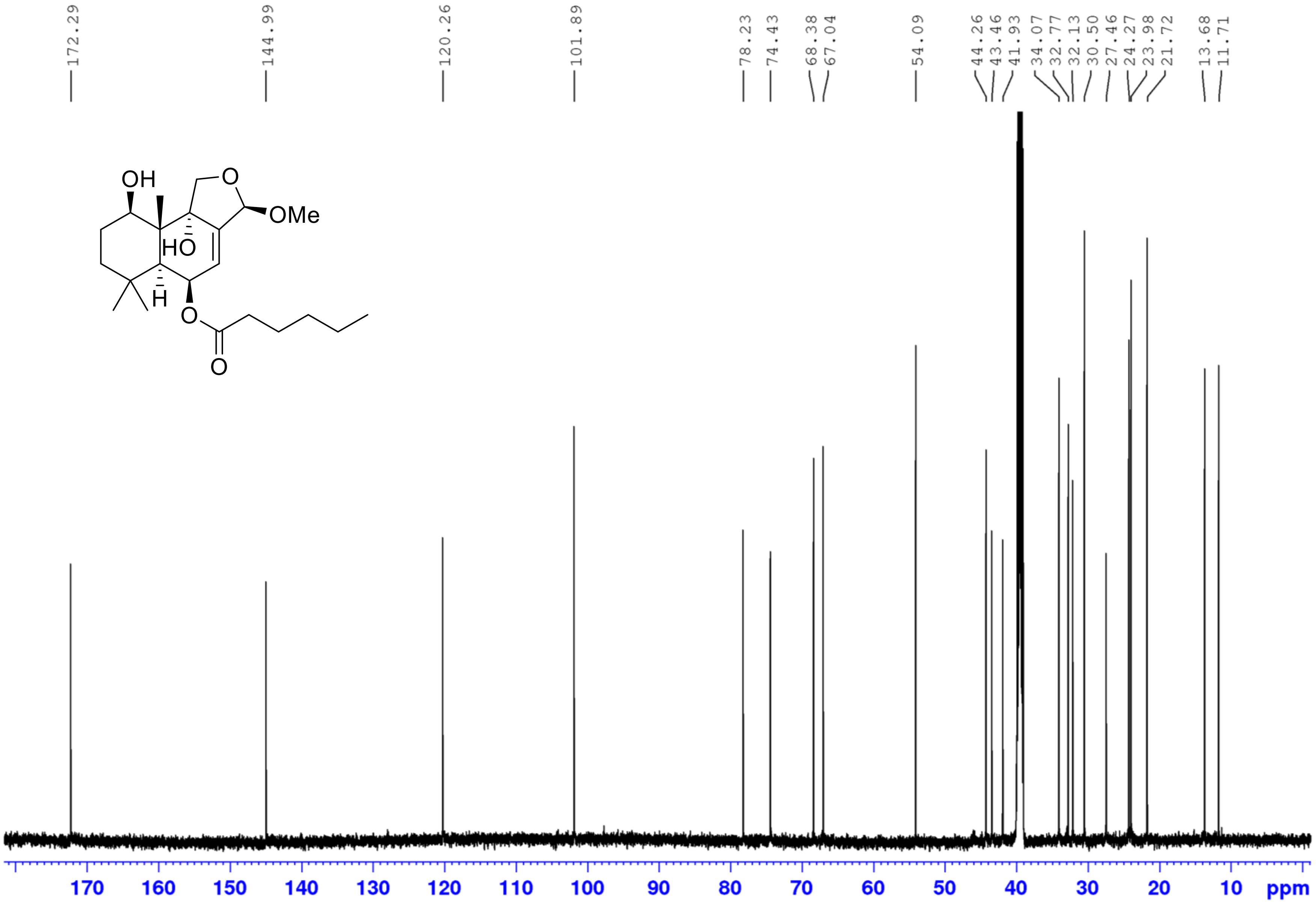

Figure S27. ^{13}C NMR spectrum (150 MHz, DMSO-d_6) of nanangenine H (**10**).

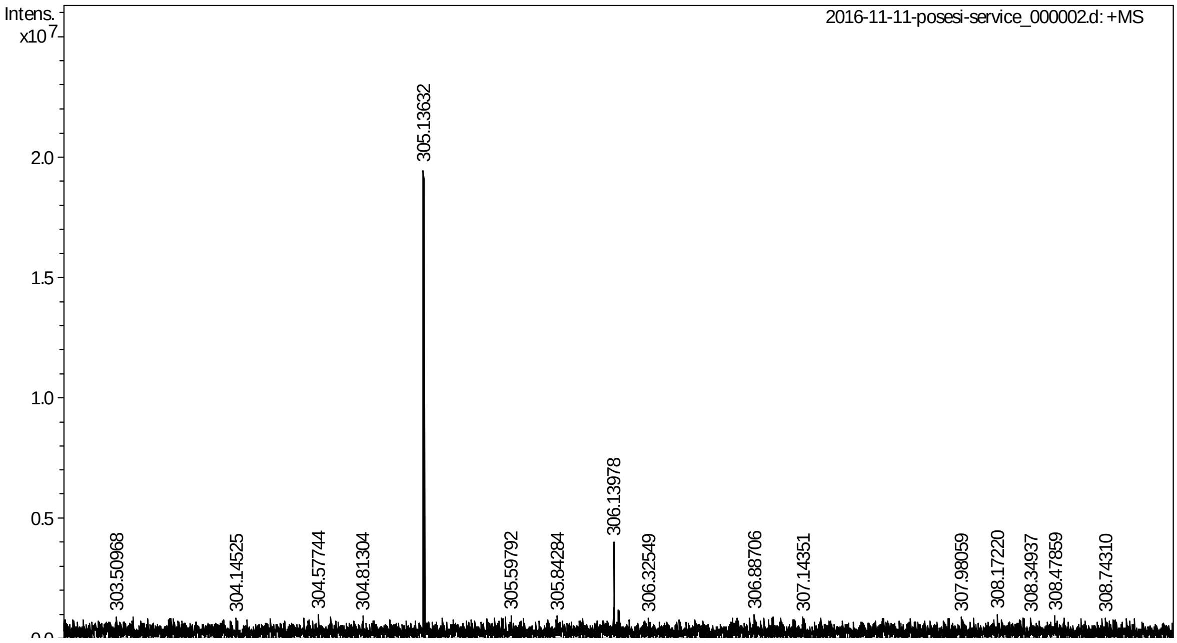

Figure S28. HRMS–ESI(+) spectrum of nanangenine A (**1**)

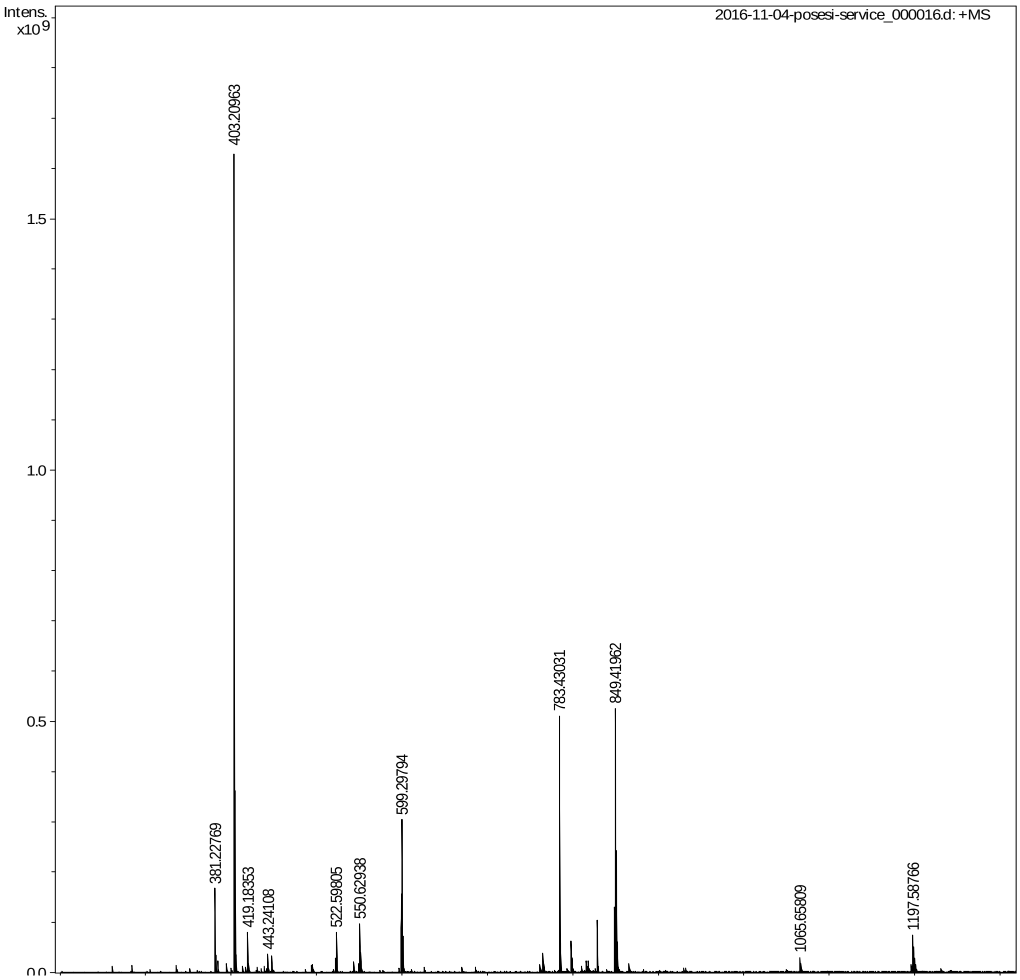

Figure S29. HRMS–ESI(+) spectrum of nanangenine B (**2**).

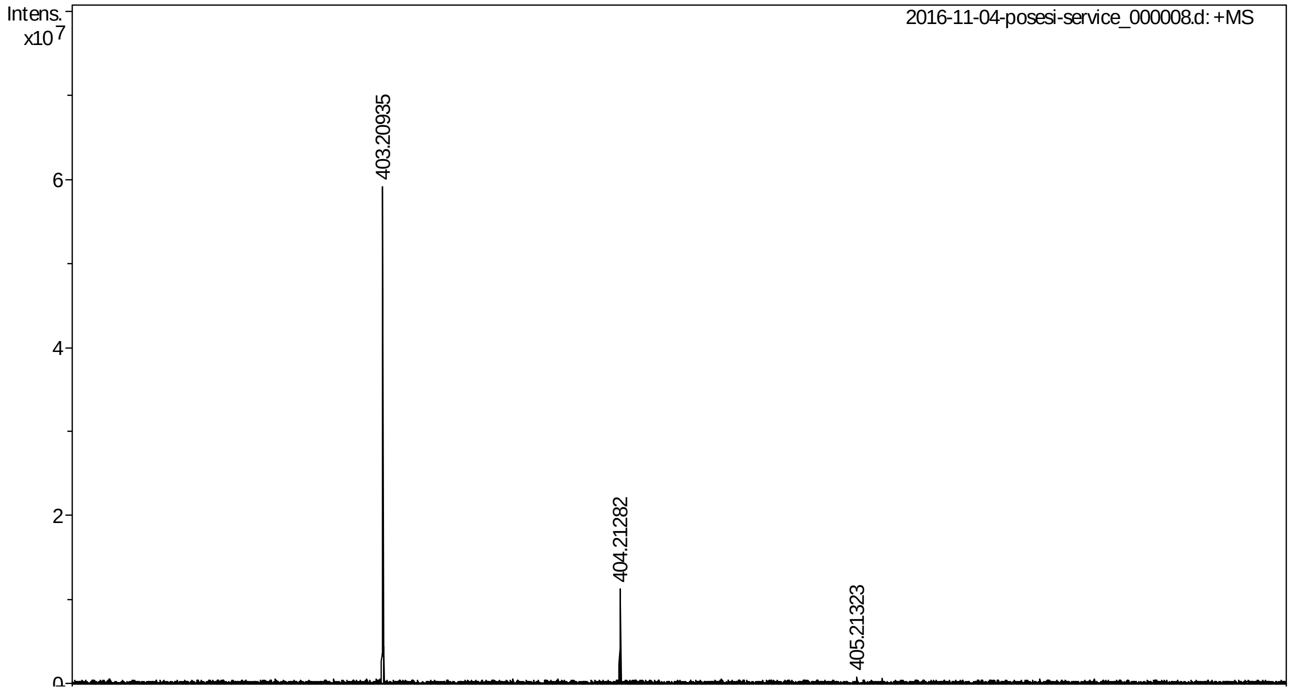

Figure S30. HRMS–ESI(−) spectrum of isonanangenine B (**3**).

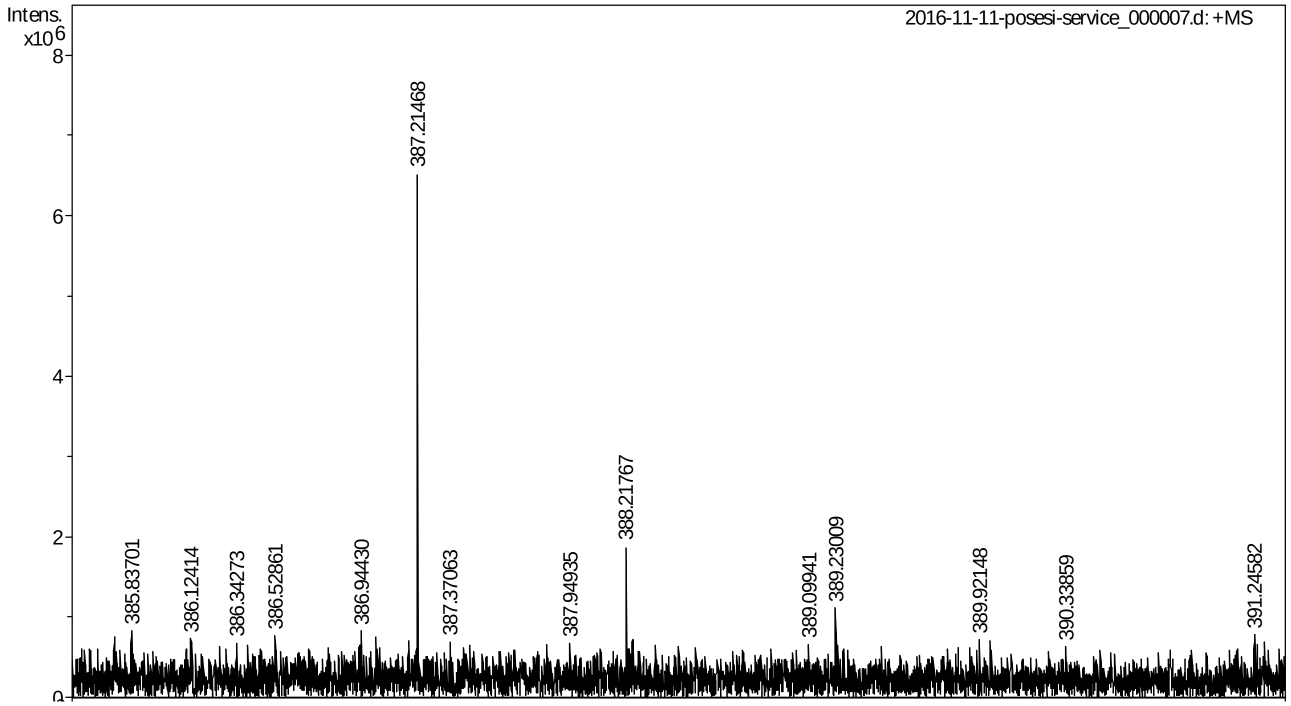

Figure S31. HRMS–ESI(−) spectrum of nanangenine C (**4**).

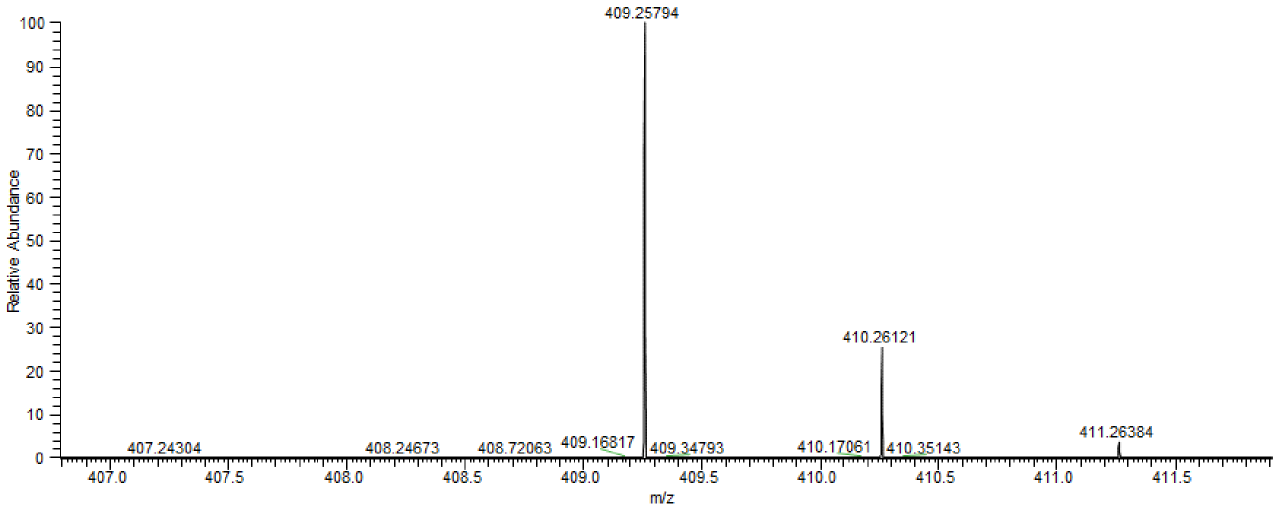

Figure S32. HRMS–ESI(+) spectrum of nanangenine D (**5**).

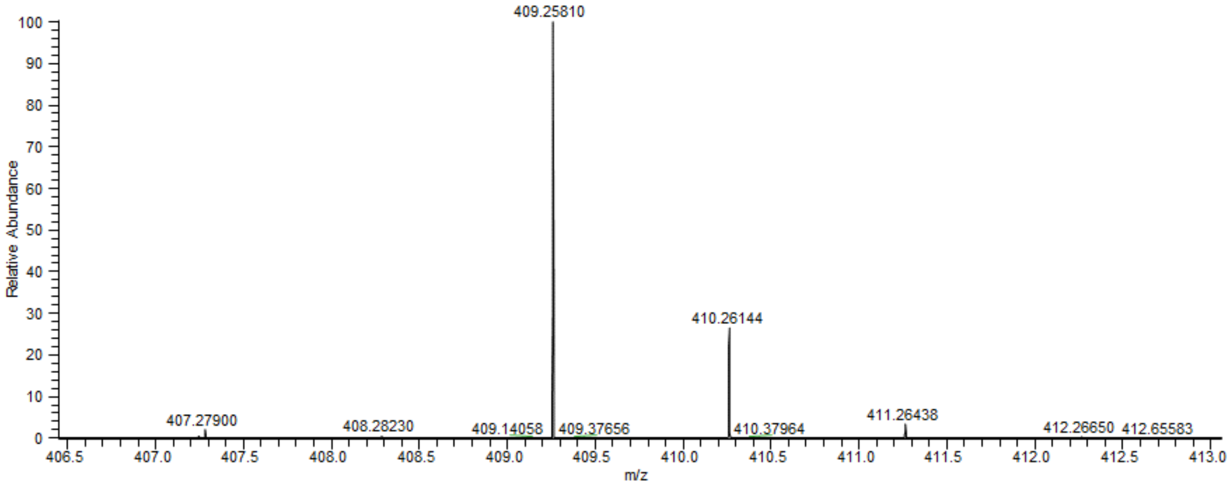

Figure S33. HRMS–ESI(+) spectrum of isonanangenine D (**6**).

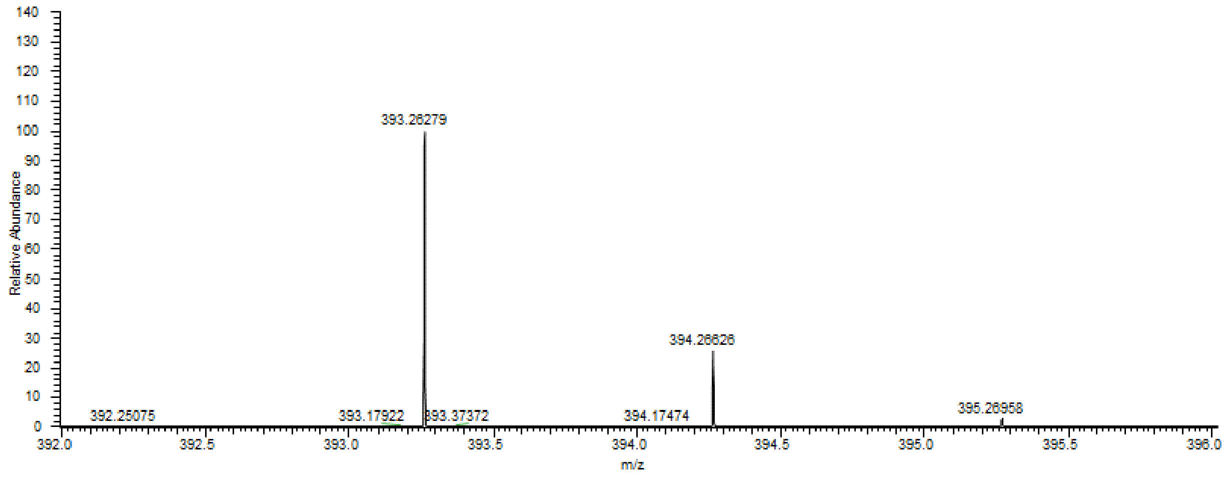

Figure S34. HRMS–ESI(+) spectrum of nanangenine E (**7**).

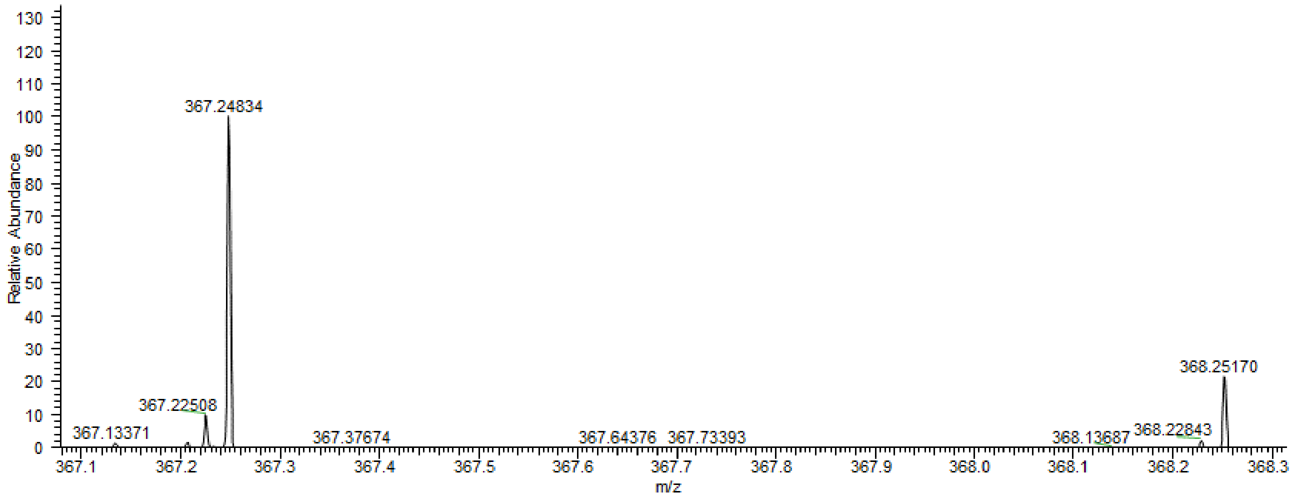

Figure S35. HRMS–ESI(+) spectrum of nanangenine F (**8**).

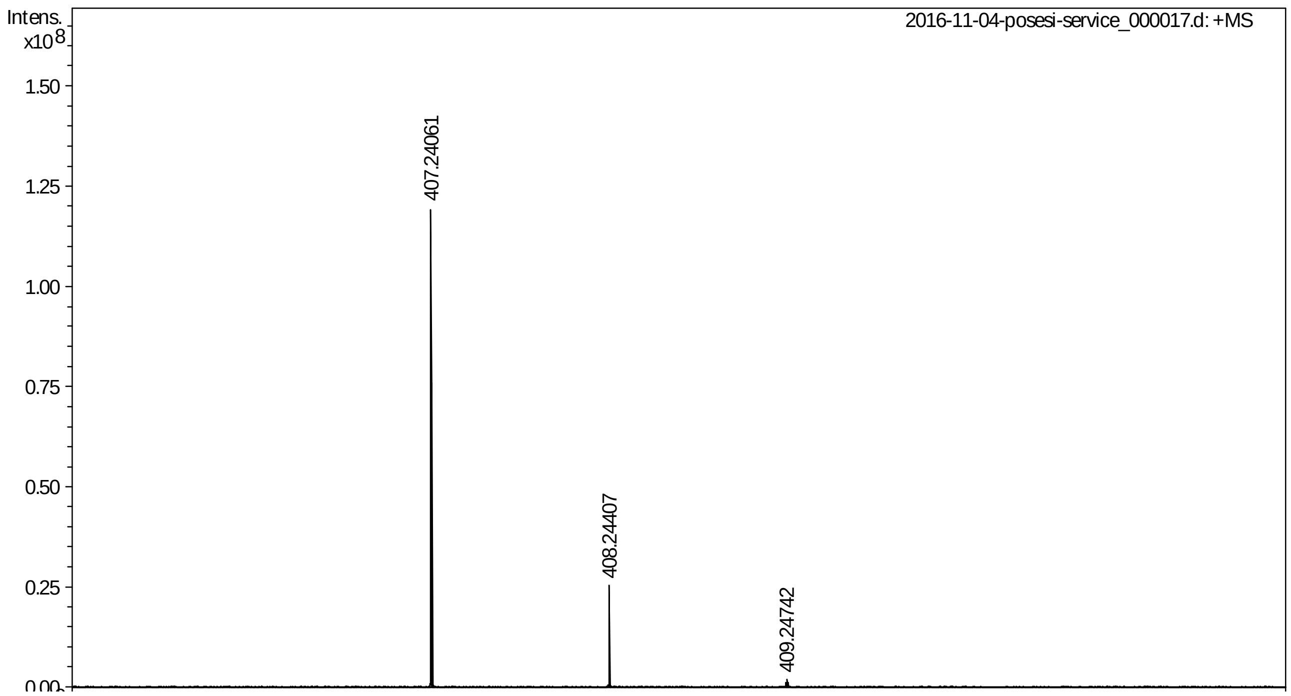

Figure S36. HRMS–ESI(+) spectrum of nanangenine G (**9**).

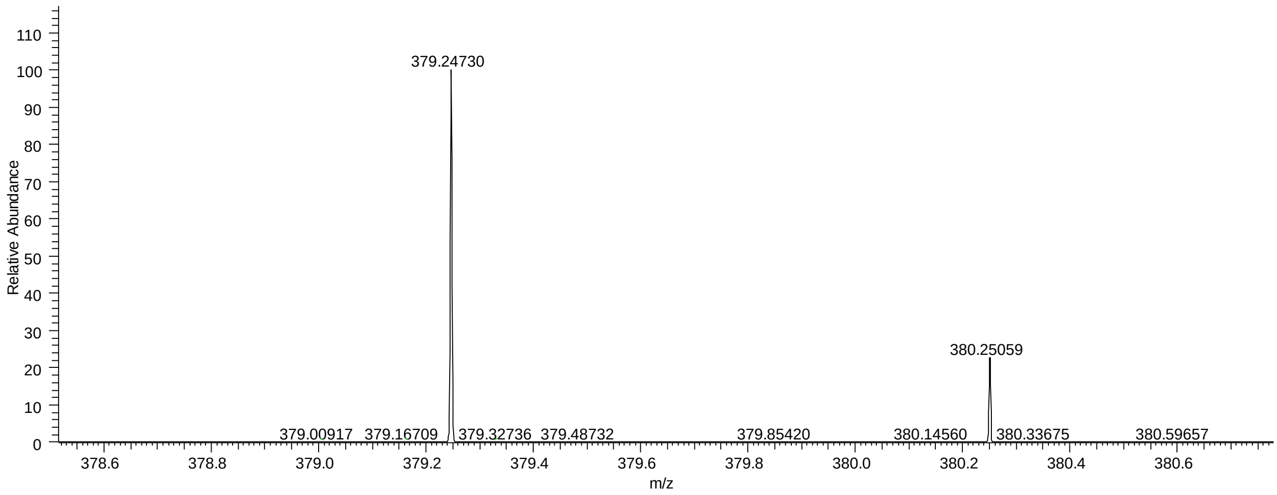

Figure S37. HRMS–ESI(+) spectrum of nanangenine H (**10**).

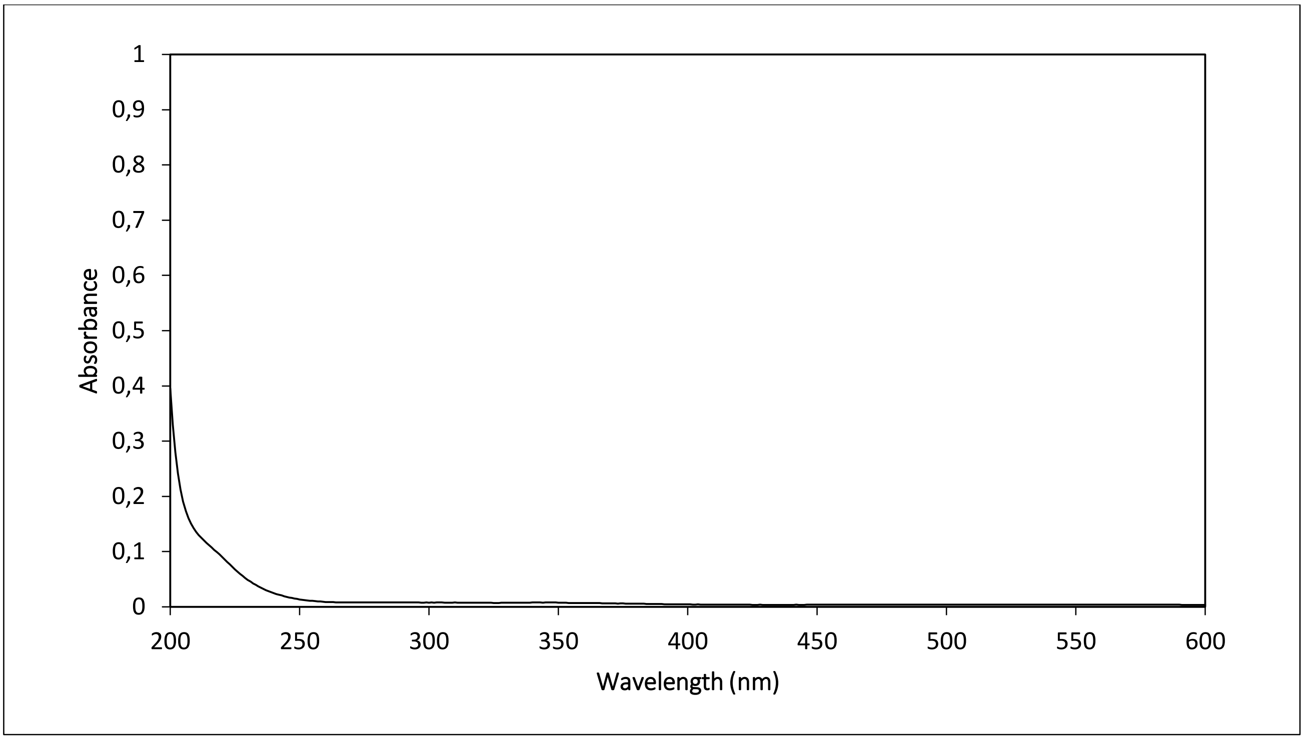

Figure S38. UV–vis spectrum (MeCN) of nanangenine A (**1**).

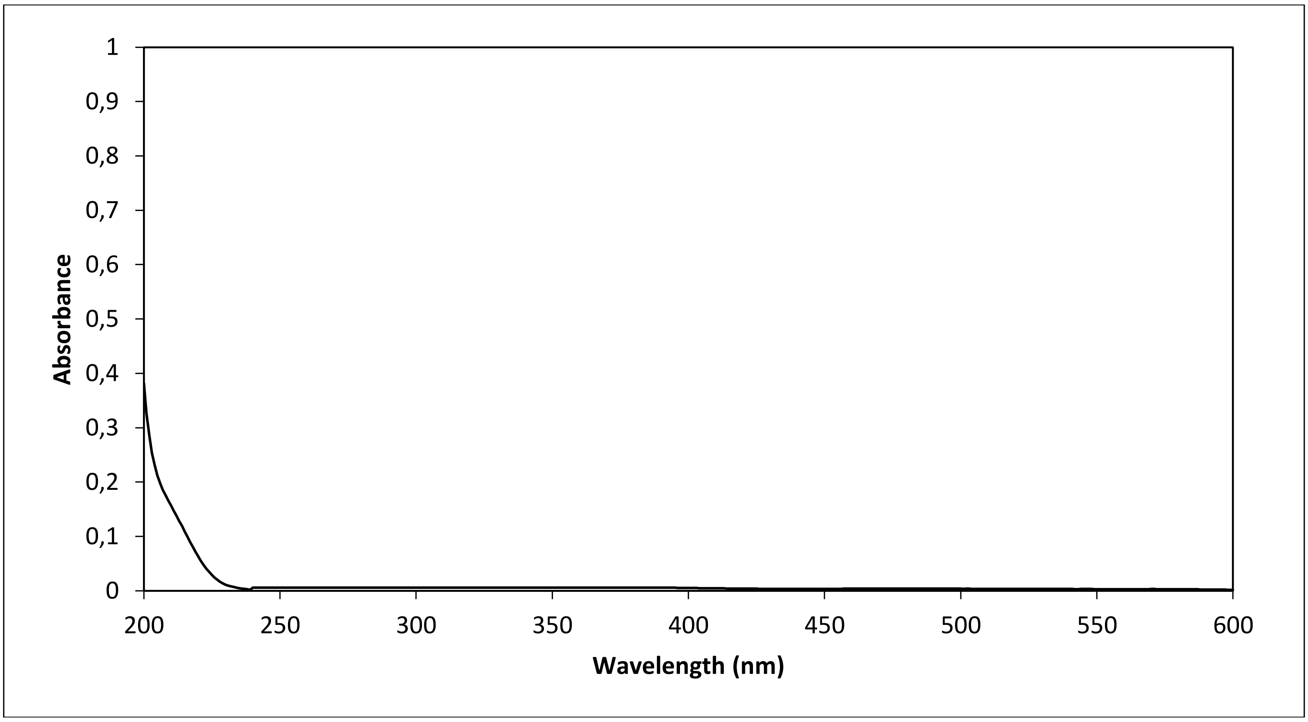

Figure S39. UV–vis spectrum (MeCN) of nanangenine B (**2**).

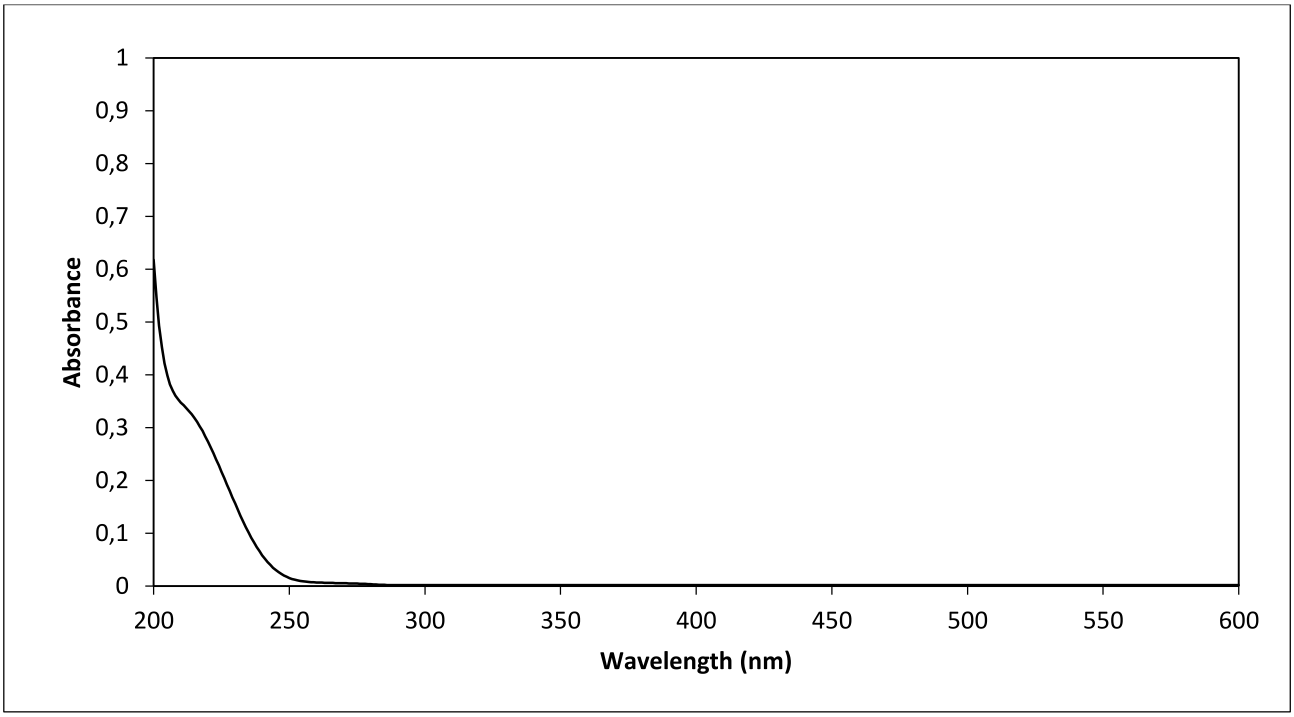

Figure S40. UV–vis spectrum (MeCN) of isonanangenine B (**3**).

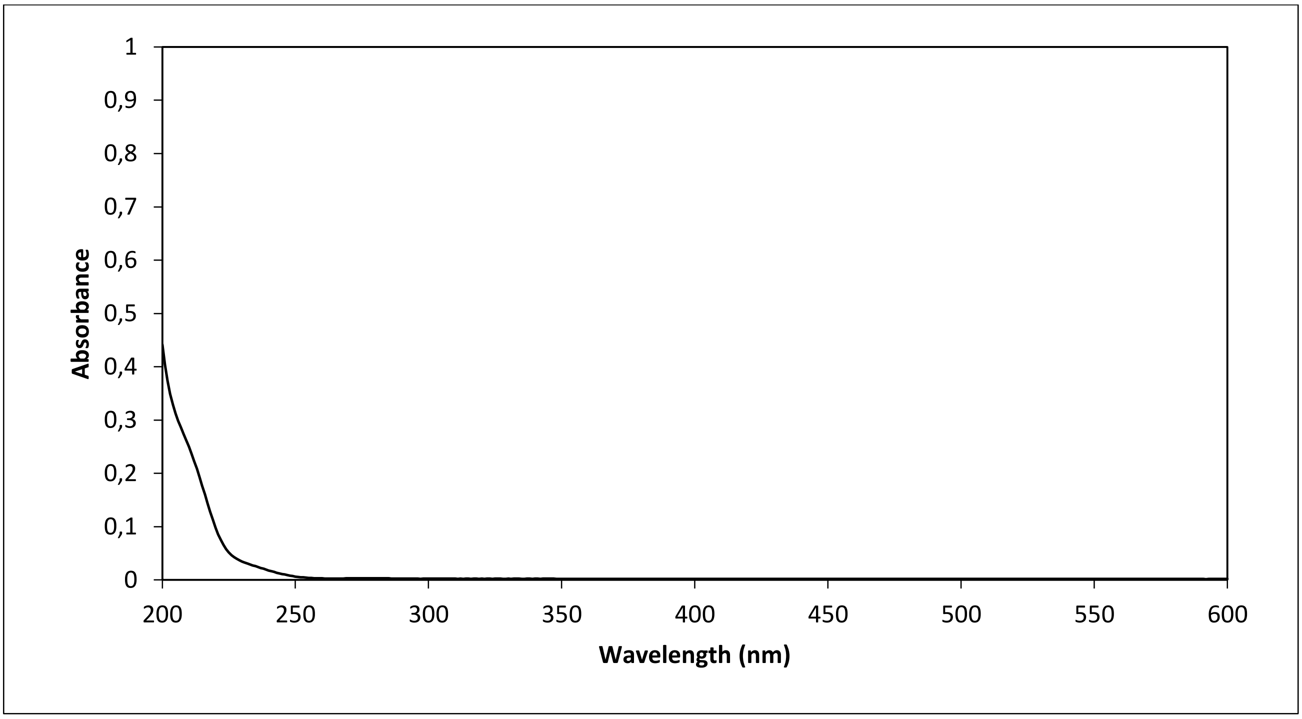

Figure S41. UV–vis spectrum (MeCN) of nanangenine C (**4**)

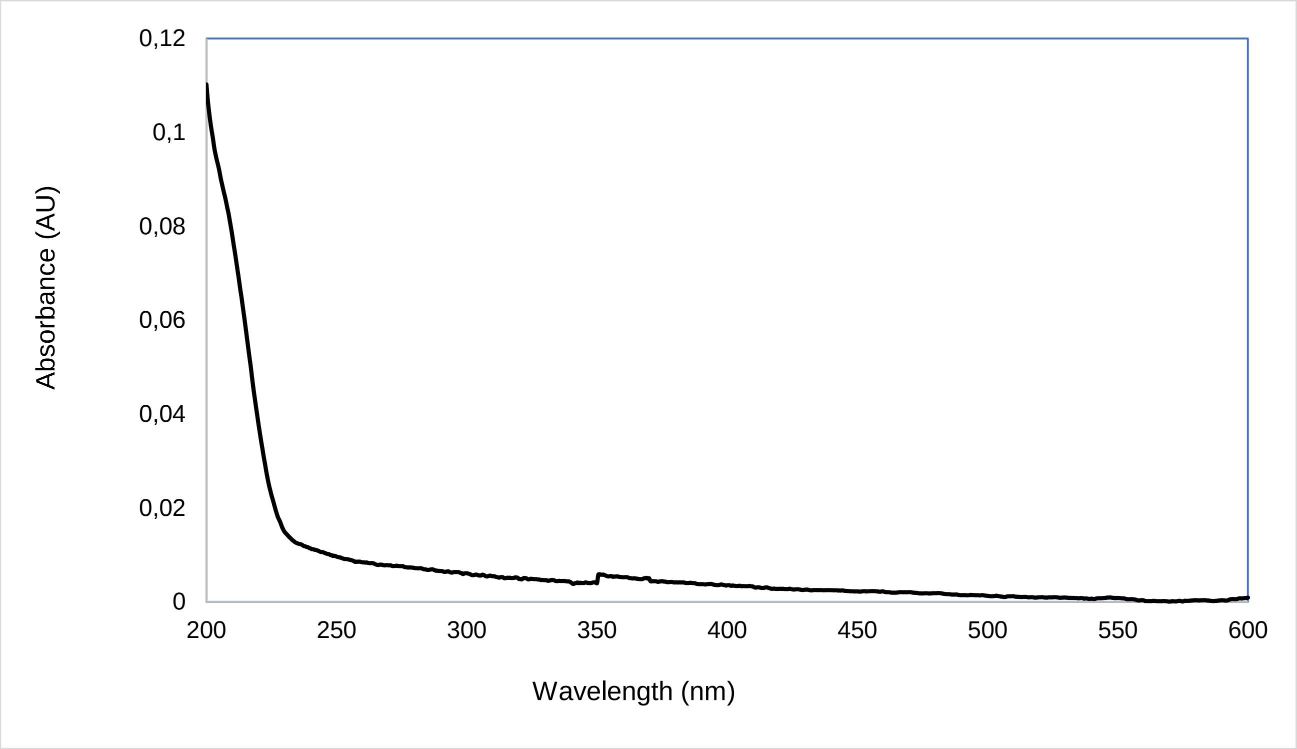

Figure S42. UV–vis spectrum (MeCN) of nanangenine D (**5**)

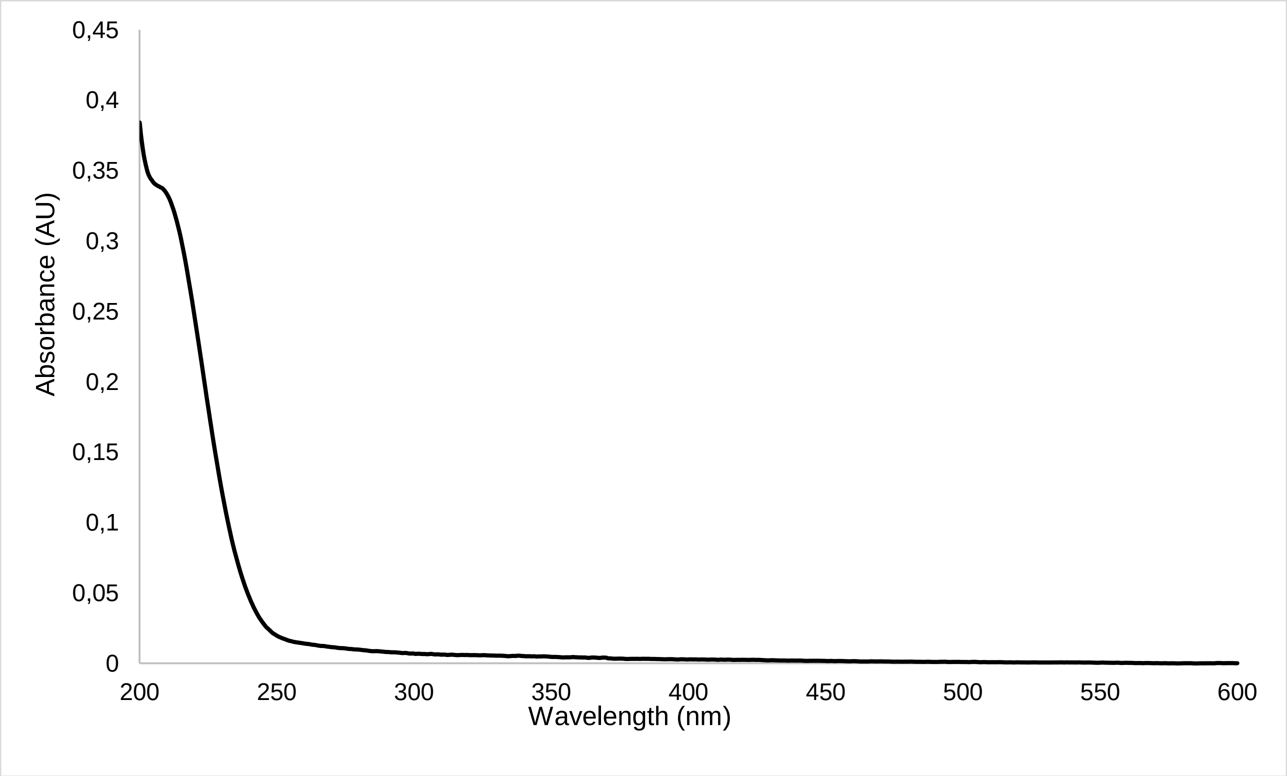

Figure S43. UV–vis spectrum (MeCN) of isonangenine D (**6**)

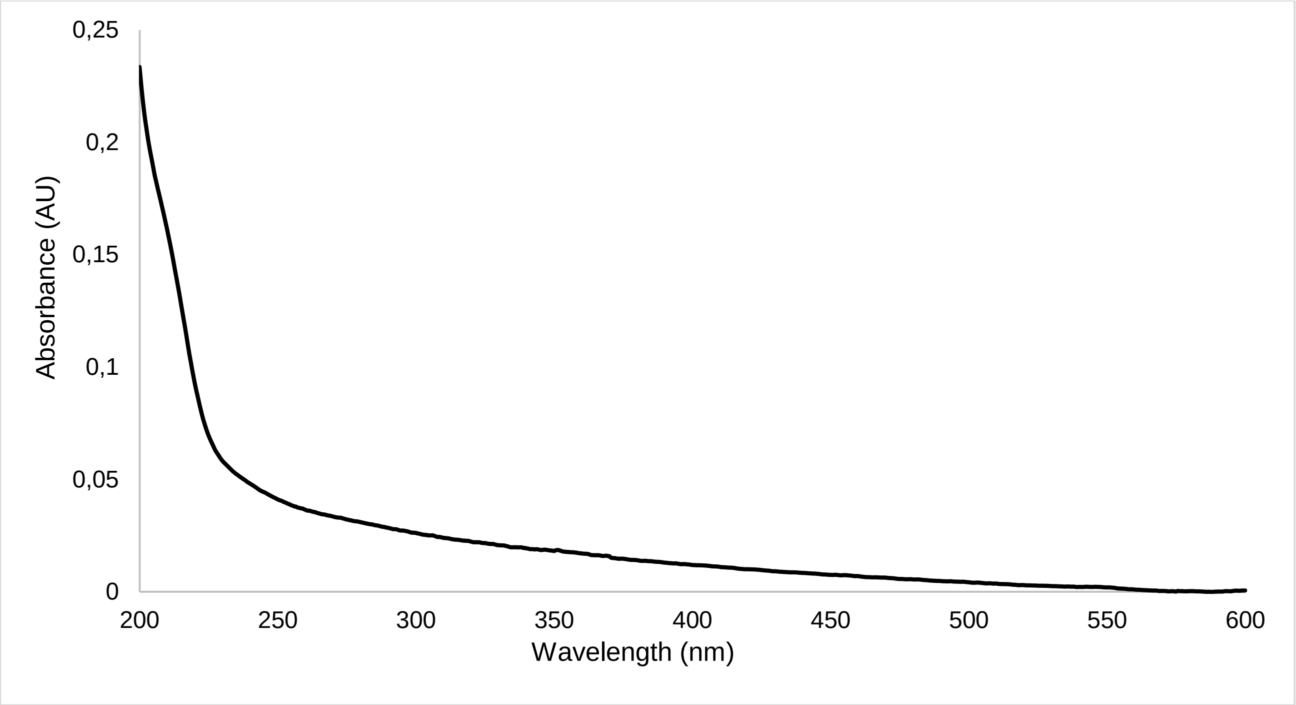

Figure S44. UV–vis spectrum (MeCN) of nanangenine E (**7**)

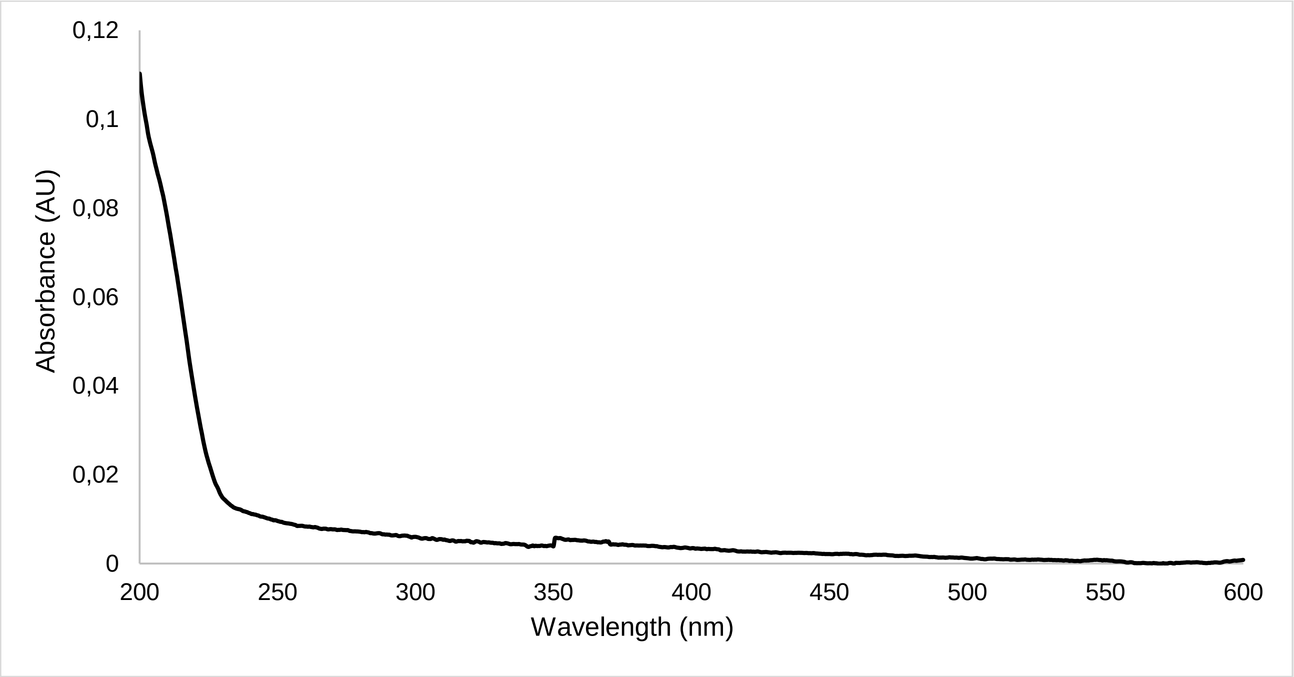

Figure S45. UV–vis spectrum (MeCN) of nanangenine F (**8**)

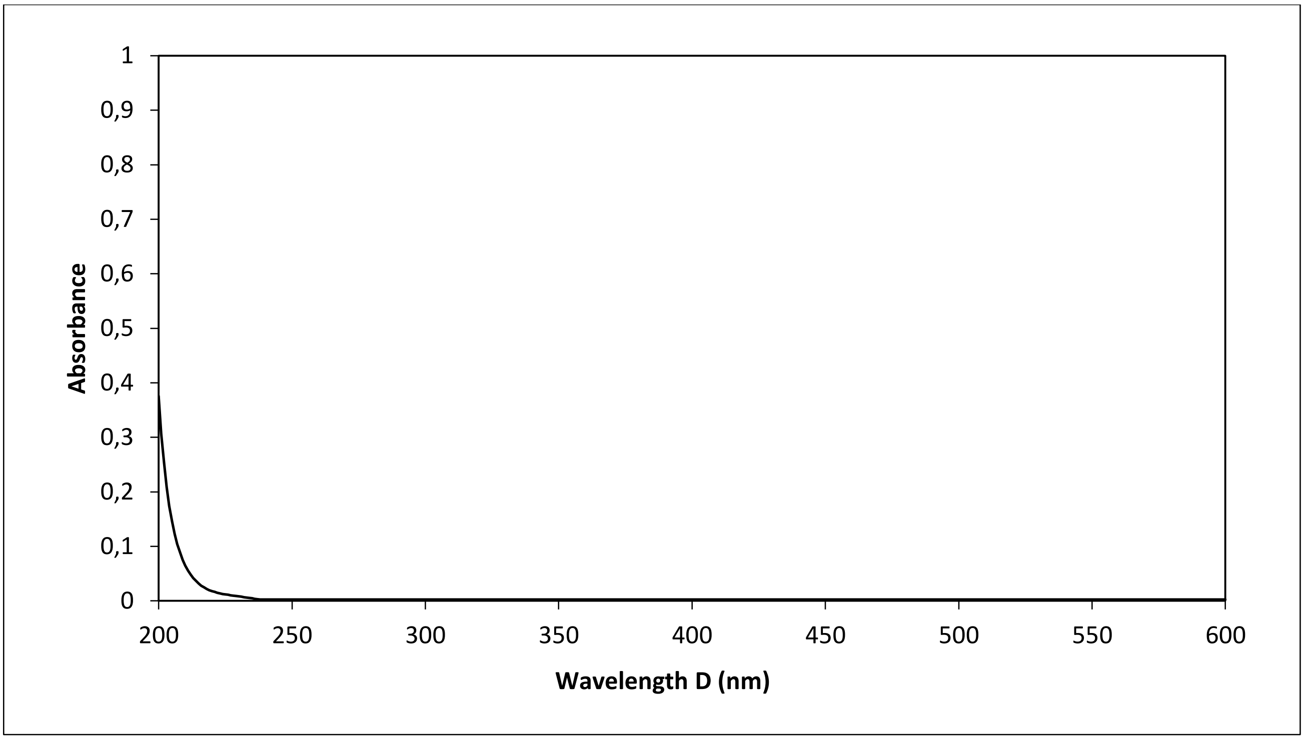

Figure S46. UV–vis spectrum (MeCN) of nanangenine G (**9**)

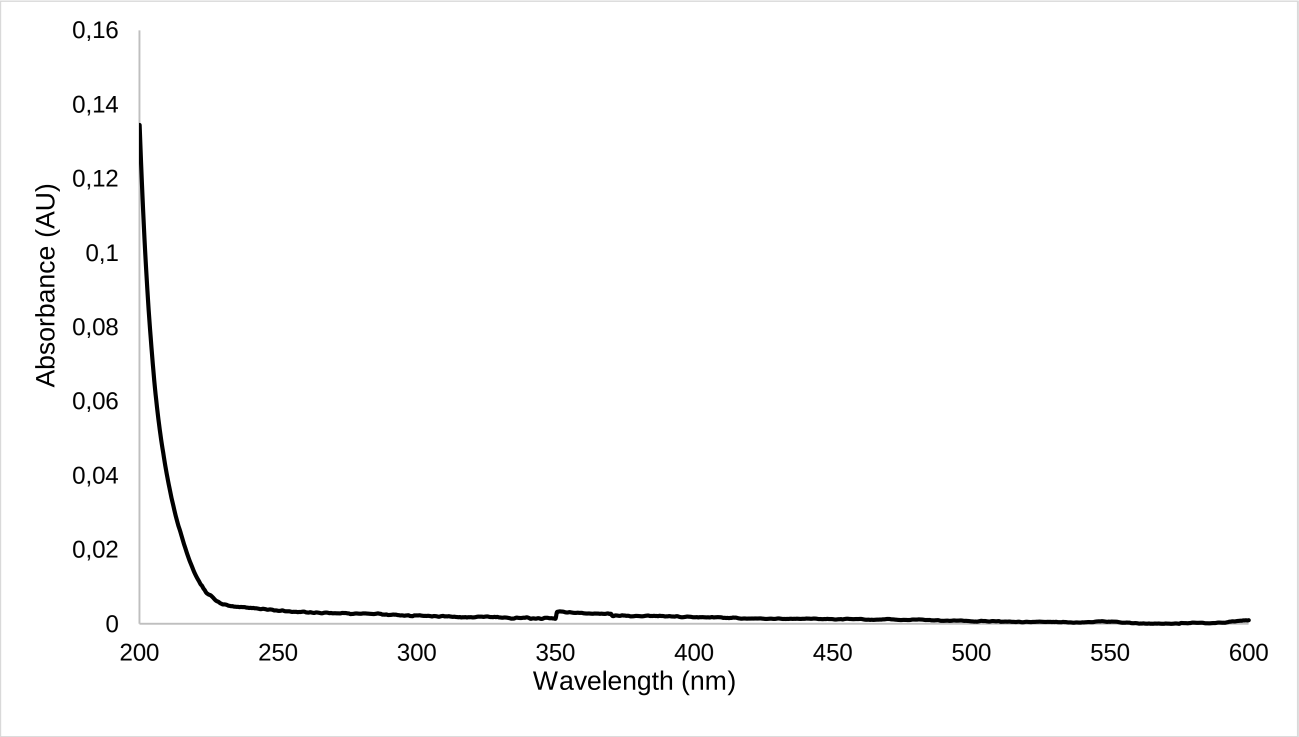

Figure S47. UV–vis spectrum (MeCN) of nanangenine H (**10**)

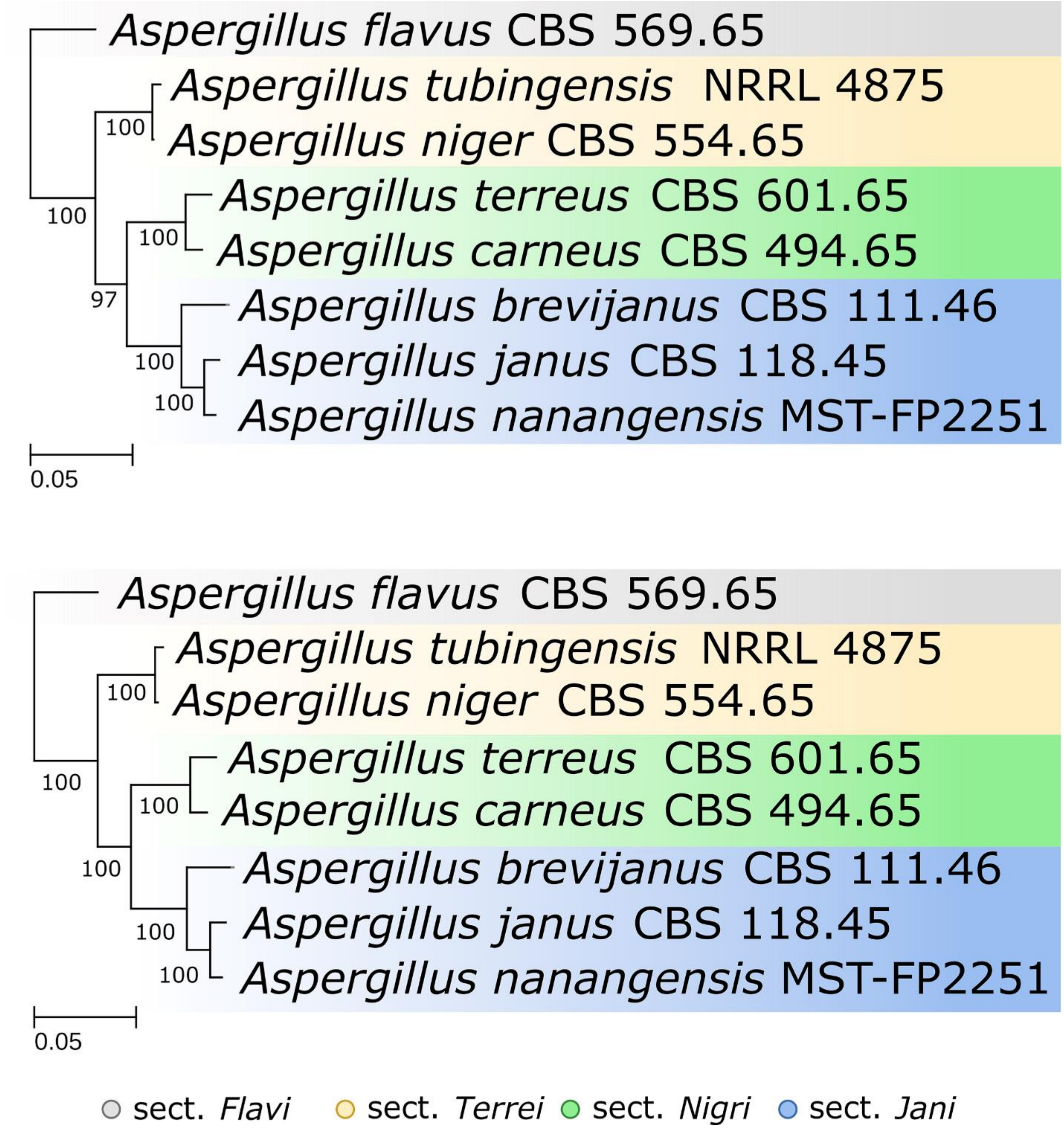

Figure S48. Maximum likelihood (ML; top) and Bayesian Inference (BI; bottom) trees of the internal transcribed spacer (ITS) region of *Aspergillus nanangensis* MST-FP2251 and other Aspergilli. *A. nanangensis* clearly clades within section *Jani*.

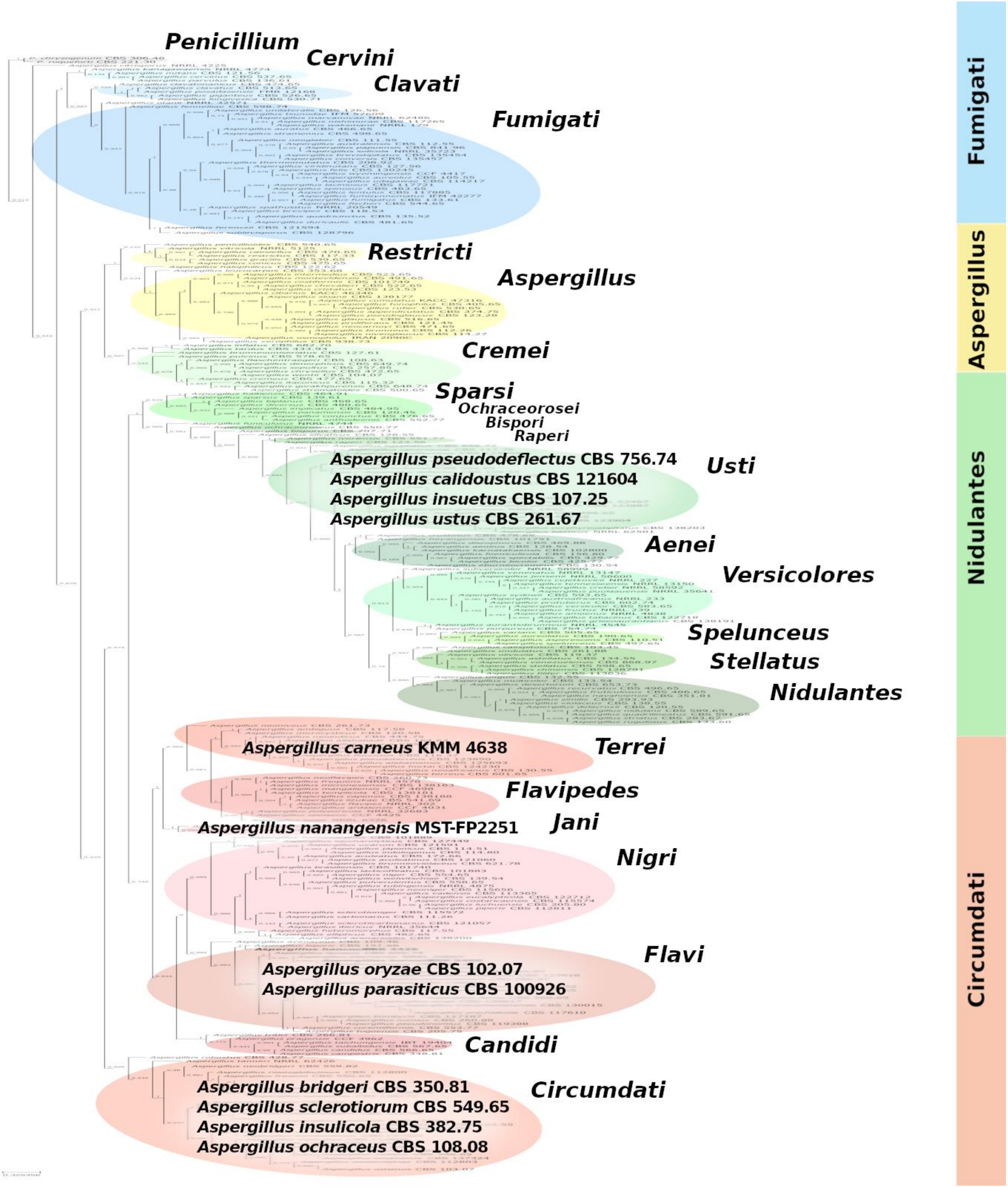

Figure S49. Taxonomic distribution of drimane sesquiterpene production across the *Aspergillus* genus. Underlying phylogenetic tree is built from a concatenated multi-locus alignment consisting of internal transcribed spacer (ITS), beta-tubulin and calmodulin sequences.

```
A_nanangensis_FE257_006542 (AstC) ----------MVRALVLDLGDVLFNWEAPKSTP----VSRKALGNILSSDIWGDYERGHYT-ESACYEALSARYGF-ETSAIAET 69
A_oryzae_AO090026000582           -----MTKINPYKGILVELKDIVFTS-SSDQIK----LPINTFKSILCCGATAQYQCGKIN-RAQYYSRLARDFAL-SLADVTAL 73
A_terreus_XP_001217376            ----MAITKGPVKALILDFSNVLCSWKPPSNVA----VPPQILKMIMSSDIWHDYECGRYS-REDCYARVADRFHI-SAADMEDT 75
A_niger_XP_001391086              MVVAVPVTSSPDRAILVELNHVIFKS-SFVDVG----IPENTYKSILCCGASTEYQCGKAG-LDQYYSRLSKDFDT-PKSQIHDM 78
A_bisporus_XP_006461126           ----MAPPQRPFTAIVFDIGDVLFQWSATTKTS----ISPKTLRSILNCPTWFDYERGRLA-ENACYAAISQEFNV-NPDEVRDA 75
T_atroviride_XP_013943363         MPHSTLTRPKSFHAIVLDLNGVLLSYGGSFPSS---VLKPSQIKNVLDSPTWYDYECGKISSRQECYQRVSSEFEM-DVDVFSDT 81
G_trabeum_XP_007864346            ----MVAAYRRFSTLICDIGDVLFTWSSTTRTT----ISARTLKAMMTSPTWRLYERGHYS-QGQCYDQLSKDFPPYEPEEIGRA 76
P_roqueforti_CDM29845             --MGKIIKRIPYKGVLIELKNIVLHS-SMQNIE----LPTNTLKSILYCGATVEYQCGRIT-EEQYFARLASDFRH-PQEEIKKA 76
L_bicolor_XP_001877382            --------------IIFDLGDVVFKWSPETKTS----ISSRTLRDILSSPTWFNYERGQLA-EEECYQQIGEEFNL-LSGEVRRA 65
A_oryzae_AO090026000576    (AstI) -----MTRQSHYQAIILDLGNVVFEWDTSQNPPTAAPNQISLLRTSMKSPVYHSYERGQLS-TEECHRLLGESLHV-DPGQIKEA 78
A_oryzae_AORIB40_NS.05916  (AstK) -------MCTTFKAAIFDMGGVLFTWNPIVDTQ----VSLKDLGTIINSETWEQFERGKIE-PDDCYHQLGSQIGL-PGSEIAAT 72

A_nanangensis_FE257_006542 (AstC) FVLARESLKIDTTFKVFLHNLKAKSNGNLRVYAMSNISQPDYDILLSKASSEDWHLFDKVFPSGHVGMRKPDLEFFRHVLREIST 154
A_oryzae_AO090026000582           FDTVQATIRPEESFLAFLAELKSRFGEQLKLYAVANMSREDYAMLKSL--PIDWSLFDGVFLSADLGMRKPELRFFRHVLESISM 156
A_terreus_XP_001217376            LKQARKSLQVHHETLLFIQQVKKDAGGELMVCGMTNTPRPEQDVMHSI--NAEYPVFDRIYISGLMGMRKPSICFYQRVMEEIGL 158
A_niger_XP_001391086              FSAINKTVQVDHGILACLARLKAHCRGTLAIYGACNMSCKDFEKVQGY--SIDWNIFDGIFISGDMGISKPELRFFSHILDRLQL 161
A_bisporus_XP_006461126           FSQARDSLQANHDFISLIRELKAQANGRLRVYAMSNISLPDWEVLRMK--PADWDIFDHVFTSGAVGERKPNLAFYRHVIAATDL 158
T_atroviride_XP_013943363         LEQLTKTVKPHSEFIAAIKNIKAAF-PEIKVYGMSNISQPDYEFLKPM--ISSWGILDGFQASGQAGVRKPDNASYITFLQKFEL 163
G_trabeum_XP_007864346            MDEARDSLTSNDAMIALLRQLKSESNGQLRILAMSNISKPDYMFLRTK--PADWDVFDTVFTSSDAGERKPNLGYYRYVVEKGAI 159
P_roqueforti_CDM29845             ILAVRKSLCVNPKVVEALASMKAKSKGLFELYAVTNFSKEDYALVKFL--GFDWSLFERVFVSSDIGMQKPELRFYQHVLNQIGL 159
L_bicolor_XP_001877382            FDQARESLVADEALIDLIRDLKTQSDGRLRIFAMSNISPPDWAVLRTK--PADWSIFDQVFTSGSAGERKPNLGFYEHVLAGTGV 148
A_oryzae_AO090026000576    (AstI) FDLARQSLRSNPALLDFIRQLKQTR--GVAVYAMSNIPQAEIEYLKES-RAGDMEVFDEVFASGYVGSRKPETEFYRRVMGEIGL 160
A_oryzae_AORIB40_NS.05916  (AstK) FRQTTGCLRPDARMTSLLRELKGQ---GVAVYMMTNIPAPDFHQLREM--HYEWDLFDGIFASALEGMRKPDLEFYEHVLKQIDT 152

A_nanangensis_FE257_006542 (AstC) AAEDVVFVDDKLENVMSAWSLGMHGIVFQNKEDVQRQLANLFG-SPTERGMEYLSSRKGQLQSMTTT-----DIPVQDNFAQLLI 233
A_oryzae_AO090026000582           KPEDTILVDNDTDNILCALSMGLKGILFGST-SVPQALTNLLEYDHISRAEQFLRSHAKSLHSVTHT-----GVTIRENFAQLLI 235
A_terreus_XP_001217376            SGDAIMFIDDKLENVIAAQSVGIRGVLFQSQQDLRRVVLNFLG-DPVHRGLQFLAANAKKMDSVTNT-----GDTIQDNFAQLLI 237
A_niger_XP_001391086              APSEVIVVDHNTDNVLTAISMGMSAVLANSPDDVQRSLVNYIERNPTERGRKFLERNAKNMHSVTHT-----GVLIRENFAQLMI 241
A_bisporus_XP_006461126           QPHQTIFVDDKLENVLSARSLGFTGIVFDEPSEVKRALRNLIG-DPVQRGGEFLVRNAGKLGSITRTTAKHESIPLDENFAQLLI 242
T_atroviride_XP_013943363         DSARCVFIDDSVENTVAASALGFKGVKYSNPIEVERTLWNLLG-SPVDRGMEYMERNAKKMMLELST-----GGEQPDNFSQFII 242
G_trabeum_XP_007864346            DPHSTIFIDDKLENVLVARSLGMYGIVFDDQDKVMRALRNLLG-DPIQRGWSFMRSHAGHLESVTNT-----GVLVPETFAQLLI 238
P_roqueforti_CDM29845             SSEQVILVDDDTSNLLAAMSMGMQGVMPSDY-SLYRSILNFVDIDPIGRGTRYLHENAQKHHSFTHT-----GVPVKENFTQLLI 238
L_bicolor_XP_001877382            GPRQTIFVDDKLENVISARSLGFHGIVFDSPEPVKRALRNLTG-DPLARGQAFLHENAGNLVSVTENSDNHEAVLLQENFAQLLI 232
A_oryzae_AO090026000576    (AstI) KAERVVFVDDKEENVDVARGLGLYGVCFGGVEELRGHLLGI*                                            201
A_oryzae_AORIB40_NS.05916  (AstK) SAAETIFVDDKLENVIAAQAVGMVGLHLTDSLATCMELRQLVG-C*                                        196

A_nanangensis_FE257_006542 (AstC) LEATNHPELVAME-PGKRTWNYFIGGPNLTTDTFPDDLDTTSLALSIVPP-APEIISSVMDEIVSRLNKDGIVPTYFDDTRPRVD 316
A_oryzae_AO090026000582           LEATGDIDLVELE-YHPTTWNYFIGTPVLTQTEFPHDLDTTSLATTVLDR-PKDIANEIMDEMLKYRSDDDLMLTFFTDFKNRVD 318
A_terreus_XP_001217376            LELAQDRELVKLQ-AGKRTWNYFIGPPKLTTATFPDDMDTTSMALSVLPV-AEDVVSSVLDEMLKFVTDDGIFMTYFDSSRPRVD 320
A_niger_XP_001391086              LEASGDSTLVDIK-PHATTWNYFIDKPVLTQKNFPDDLDTTSLGLTITNA-TPEVANQVLNKILHYRTYDGLIMTFFTDFKNRVD 324
A_bisporus_XP_006461126           LEITGNRALVNLV-EHPQTWNFFQGKGQLTTEEFPFDLDTTSLGLTILKR-SREIADSVMDEMLEYVDPDGIIQTYFDHRRPRFD 325
T_atroviride_XP_013943363         LELTQDERLIKLERKRGPTWNYFHHSNTFNGTTYSDDCDTTSYAMCTLDDIPAHEKEAAMDIILNNLSPDNLPLCWFNKNRPRLC 327
G_trabeum_XP_007864346            LDVTQDRNLVALPKDCPRRWNFFSGKPILTTEVFPDDLDTTSLGLTIFET-DEAVAQSVIDEMLEYVTPDGIVQTYFDHTRPRFD 322
P_roqueforti_CDM29845             LELTGDRSLIDIG-SHRTTWNLFIVTPVLTQADFPDDMDTTSLGITILNR-PTHVANLVMDKMLQYRTSDGLMQTFFTDFKKRVD 321
L_bicolor_XP_001877382            LEVIGDANLVNLV-EHPRTWSFFQGKGQLTTEEFPFDLDTTSLGLTVMKR-DKAVANSVMNEMLEYVDHDGIIQTYFDHRRPRFD 315

A_nanangensis_FE257_006542 (AstC) PIVCINVSTLFAKHHRESQLSTTISWIRDVLYHRAYFAGTRYYASPEAFLFFFTRLTSTLRPG--ALKDDFAGLLTERLRERIGT 399
A_oryzae_AO090026000582           PVVCCNVLSLFYKYGRGHELHHTLAWVRQVLIRRAYINGTAFYPMPEAFLYFFFRFLQHITHLP-QLYDGLKVLLKERLQERVGV 402
A_terreus_XP_001217376            PVVCINVLGVFCRHNRERDVLPTFHWIRDILINRAYLSGTRYYPSPDLFLFFLARLCLAVRNQ--SLREQLVLPLVDRLRERVGA 403
A_niger_XP_001391086              PVVCCNVLSLFYQYGRGEEVSDTFDWVQLVLRRRAYIHGTAFYPSPEAFLFFFSRLLRRLESPPTPTYNELEQLLRERVAERIGV 409
A_bisporus_XP_006461126           PVVCVNALSLFYAYGRGEQLRSTLTWVHEVLLNRAYLDGTRYYETAECFLYFMSRLLATSGDP--DLHSLLKPLLKERVQERIGA 408
T_atroviride_XP_013943363         HGIIANAFRFFALSGQGHKLAHTYLFLCRLLRTKAYELGSRYYENIDYMPYILSNLCSRRPTD--PSLAEMRELLKKEIQDRSGC 410
G_trabeum_XP_007864346            PVVCVNVLSLFYSHNRKQELRGTLIWVREVLLNRAYLDGTRYYASAECFLYFLSRLLRKAEDH--ELNDLLYNLLKERVQERIGV 405
P_roqueforti_CDM29845             PIVCCNILNIFYQYGWGNELSETFDWVYQVLQTRTYIHGSAFYPLPEAFFFFLSRMMLRLKNHRPCVYIRMRGLLIKRLEERLSV 406
L_bicolor_XP_001877382            PVVCVNALTLFYTHGRGSELSRTLQWIHKVLLNRAYLDGTRYYQTAECFLFFLSRLLASSEDR--ELHALLKPLLRERIQERIGV 398

A_nanangensis_FE257_006542 (AstC) PVDSLALAMRVLSCQALGIEC-AADVGRLSEMQCADGGWPPCVIYKYGSGGLGITNRGVSTAFAVRAIAQFATTSSASA---GMA 480
A_oryzae_AO090026000582           PVDPISLSMRLIACNGVGIHD-RMGLNALLSMQNPDGSWDLGTMYHYASKRLPIGNQGVSTAMAIKAIKQCQANQCAGI------ 480
A_terreus_XP_001217376            PGEAVSLAARILACRSFGIDS-ARDMDSLRGKQCEDGGWPVEWVYRFASFGLNVGNRGLATAFAVRALESPYGESAVKVM----R 483
A_niger_XP_001391086              PVDAISLAMRLLVCHQVGMRD-TLGLEMLLSMQQPDGGWPLGTIYHYASKKQAIGNRGVSTALAVQAIDVCSQWKKSPNGHPKAT 493
A_bisporus_XP_006461126           DGDSLALAMRILACDFVGIRD-EVDLRTLLTLQCEDGGWEVGWMYKYGSSGISIGNRGLATALAIKAVDTMFQPQIRFSESPTDT 492
T_atroviride_XP_013943363         DSDVLGAALRTLSAQAMGVPYAKRDVQVLLESQQLDGGWNRVWLFKYGKEDIKVGSRGVITAMAVKALRQYYADEE--------- 486
G_trabeum_XP_007864346            EGDALALAMRILTCAAVGIRD-DVDMRTLLPLQCEDGGWELGWIYKYGSSGVNIGNRGLATAFAIKAIEALEDMPASPPTSGTTT 489
P_roqueforti_CDM29845             PVDAASLAMRLIVCHQVGVRH-VSGLKFLLSMQEPDGGWEIGTLYQYYSKRLWLGNRGTSTALALDAIRRCQPWLAHRF------ 484
L_bicolor_XP_001877382            EGDSIALAMRILVCDFVGLRD-EIDLRSLLPLQCEDGGWEIGWIYKYGSSGLRIGNRGLTTALALNAL----------------- 465

A_nanangensis_FE257_006542 (AstC) RVDGEGKGEVGRSSPSHGGSRSGTLSAQAKA-LSPKWGLQSLLIRILPALFGVAGVAVVLVFRV    543
A_oryzae_AO090026000582           ----------------------------------------------------------------    480
A_terreus_XP_001217376            RIV-------------------------------------------------------------    486
A_niger_XP_001391086              VYTRTERHY---GSP-------------------------------------------------    505
A_bisporus_XP_006461126           LVENAI---H-KRRPSFSEKFLGKRPRSGSFRKPLQWILQGSKLRKSVEI-GS-----------    540
T_atroviride_XP_013943363         ----------------------------------------------------------------    486
G_trabeum_XP_007864346            PVKHEE---EAPRRVKRSSSRIRNHAVQAHF---------------------------------    517
P_roqueforti_CDM29845             ----------------------------------------------------------------    484
L_bicolor_XP_001877382            ----------------------------------------------------------------    465
```

Pairwise Identity (%) Matrix

A_oryzae_AO090026000582	100.00	34.17	51.25	39.58	23.33	37.92	51.04	39.14	36.46
A_terreus_XP_001217376	34.17	100.00	33.54	39.92	27.37	40.53	32.64	40.65	43.00
A_niger_XP_001391086	51.25	33.54	100.00	37.43	22.63	36.44	49.59	39.35	35.64
A_bisporus_XP_006461126	39.58	39.92	37.43	100.00	29.01	56.87	35.74	72.26	42.04
T_atroviride_XP_013943363	23.33	27.37	22.63	29.01	100.00	26.54	22.93	25.38	27.78
G_trabeum_XP_007864346	37.92	40.53	36.44	56.87	26.54	100.00	34.50	60.65	42.17
P_roqueforti_CDM29845	51.04	32.64	49.59	35.74	22.93	34.50	100.00	36.77	33.06
L_bicolor_XP_001877382	39.14	40.65	39.35	72.26	25.38	60.65	36.77	100.00	45.16
A_nanangensis_FE257_006542	36.46	43.00	35.64	42.04	27.78	42.17	33.06	45.16	100.00

Figure S50. Multiple sequence alignment of FE257_006542, AstC and orthologous proteins. The class I terpene synthase DDxxD motif is highlighted in **bold blue** text; class II DxDTT and QW motifs are highlighted in **bold red** text. Asterisk (*) in bold red text indicates stop codon. Pairwise identity matrix table (bottom): columns correspond to the same sequences in the same order as the rows.

```
A_nanangensis_MST-FP2251     AGGATCATTACCGAGTGAGGGTCCTCGTGGCCCAACCTCCCACCCGTGTCTATCTGTACCCTGTTGCTTCGGCGGGCCCGCCGCT     85
A_janus_NRRL_1787            AGGATCATTACCGAGTGAGGGTCCTTGTGGCCCAACCTCCCACCCGTGTCTATCTGTACCCTGTTGCTTCGGTGGGCCCGCCGCC     85
Uncultured_fungus            AGGATCATTACCGAGTGAGGGTCCTCGTGGCCCAACCTCCCACCCGTGTCTATCTGTACCCTGTTGCTTCGGCGGGCCCGCCGCT     85

A_nanangensis_MST-FP2251     CAGTCGGCCGCCGGGGGGCTTTACAGCCCCCAGGCCCGTGCCCGCCGGAGACCCCCAACTGAACCCTGTTCTGAAAGCTTGCAGT     170
A_janus_NRRL_1787            TGT-CGGCCGCCGGGGGGCTCTCCAGCCCCCGGGCCCGTGCCC-ACCGAAGACCCCAACTGAACCCTGTTCTGAAAGCTTGCAGT     168
Uncultured_fungus            CAGTCGGCCGCCGGGGGGCTTTACAGCCCCCAGGCCCGTGCCCGCCGGAGACCCCCAACTGAACCCTGTTCTGAAAGCTTGCAGT     170

A_nanangensis_MST-FP2251     CTGAGTTGATTATCATTAATCAGTTAAAACTTTCAACAATGGATCTCTTGGTTCCGGCATCGATGAAGAACGCAGCGAAATGCGA     255
A_janus_NRRL_1787            CTGAGTTGATTATCATTAATCAGTTAAAACTTTCAACAATGGATCTCTTGGTTCCGGCATCGATGAAGAACGCAGCGAAATGCGA     253
Uncultured_fungus            CTGAGTTGATTATCATTAATCAGTTAAAACTTTCAACAATGGATCTCTTGGTTCCGGCATCGATGAAGAACGCAGCGAAATGCGA     255

A_nanangensis_MST-FP2251     TAACTAATGTGAATTGCAGAATTCAGTGAATCATCGAGTCTTTGAACGCACATTGCGCCCCCTGGTATTCCGGGGGGCATGCCTG     340
A_janus_NRRL_1787            TAACTAATGTGAATTGCAGAATTCAGTGAATCATCGAGTCTTTGAACGCACATTGCGCCCTCTGGTATTCCGGAGGGCATGCCTG     338
Uncultured_fungus            TAACTAATGTGAATTGCAGAATTCAGTGAATCATCGAGTCTTTGAACGCACATTGCGCCCCCTGGTATTCCGGGGGGCATGCCTG     340

A_nanangensis_MST-FP2251     TCCGAGCGTCATTGCTGCCCTCAAGCCCGGCTTGTGTGTTGGGTCCTCGTCCCCCTTCCCGGGGGGACGGGCCCGAAAGGCAGCG     425
A_janus_NRRL_1787            TCCGAGCGTCATTGCTGCCCTCAAGCCCGGCTTGTGTGTTGGGTCCTCGTCCCCCTTCCCGGGGGGACGGGCCCGAAAGGCAGCG     423
Uncultured_fungus            TCCGAGCGTCATTGCTGCCCTCAAGCCCGGCTTGTGTGTTGGGTCCTCGTCCCCCTTCCCGGGGGGACGGGCCCGAAAGGCAGCG     425

A_nanangensis_MST-FP2251     GCGGCACCGCGTCCGGTCCTCGAGCGTATGGGGCTTTGTCACCCGCTCAGTAGGCCCGGCCGGCGCCTGTCGACCCCCAACCTTT     510
A_janus_NRRL_1787            GCGGCACCGCGTCCGGTCCTCGAGCGTATGGGGCTTTGTCACCCGCTCAGTAGGTCCGGCCGGCGCCTGTCGACCCCCAACCTTT     508
Uncultured_fungus            GCGGCACCGCGTCCGGTCCTCGAGCGTATGGGGCTTTGTCACCCGCTCAGTAGGCCCGGCCGGCGCCTGTCGACCCCCAACCTTT     510

A_nanangensis_MST-FP2251     TATTATTTTTCAGGTTGACCTCGGATCAGGTAGGGATACCCGCTGAACTTAA                                      562
A_janus_NRRL_1787            TATTT-TTTTCAGGTTGACCTCGGATCAGGTAGGGATACCCGCTGAACTTAA                                      559
Uncultured_fungus            TATTATTTTTCAGGTTGACCTCGGATCAGGTAGGGATACCCGCTGAACTTAA                                      562
```

Pairwise identity (%) matrix

A_nanangensis_MST-FP2251	100.00	94.99	100.00
A_janus_NRRL_1787	94.99	100.00	94.99
Uncultured_fungus	100.00	94.99	100.00

Figure S51. Multiple sequence alignment of internal transcribed spacer (ITS) regions from *A. nanangensis* MST-FP2251, *A. janus* NRRL 1787 and an uncultured fungus previously isolated from soil near Nanango, Queensland. Pairwise identity matrix table (bottom): columns correspond to the same sequences in the same order as the rows.

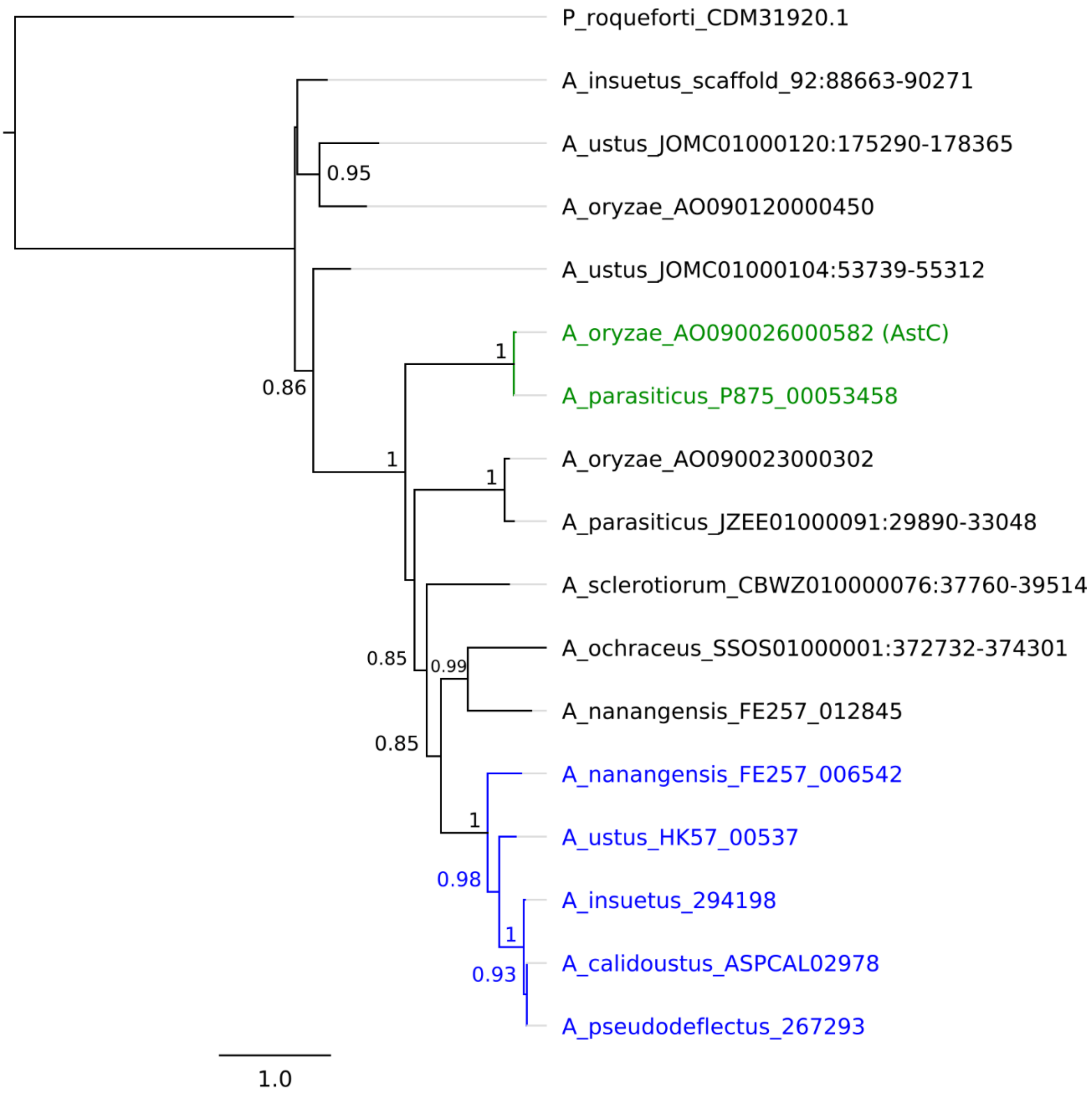

Figure S52. Phylogeny of AstC, the sesquiterpene synthase involved in astellolide C biosynthesis, homologs identified in available genomes of drimane sesquiterpene producing species *A. insuetus*, *A. oryzae*, *A. parasiticus*, *A. ustus*, *A. nanangensis*, *A. calidoustus and A. pseudodeflectus.*

Only support values above 0.8 are shown. Green highlighted clade contains AstC. The putative nanangenine cluster terpene synthase and the homologs in the drimane sesquiterpenoid producing-Aspergilli from section *Usti* form a distinct clade which is highlighted blue. Sequences were identified via local tBLASTn [17] searches against each genome; names include a locus tag if available, otherwise the coordinates on the assembly scaffold is indicated. Amino acid alignments were built with MUSCLE v3.8.31; the tree was constructed with FastTree 2.1.5 [15] in Geneious 10.2.6 [18] using the JTT model of amino acid substitution.

Supplementary references

1. Hübschle, C. B.; Sheldrick, G. M.; Dittrich, B. "ShelXle: A Qt Graphical User Interface for SHELXL". *J. Appl. Crystallogr*. **2011**, *44*, 1281–1284. https://doi.org/10.1107/S0021889811043202.
2. Farrugia, L. "WinGX Suite for Small-Molecule Single-Crystal Crystallography". *J. Appl. Crystallogr*. **1999**, *32*, 837–838.
3. Farrugia, L. J. "WinGX and ORTEP for Windows: An Update". *J. Appl. Crystallogr*. **2012**, *45*, 849–854. https://doi.org/10.1107/S0021889812029111.
4. Spek, A. L. "Structure Validation in Chemical Crystallography". *Acta Crystallogr. D Biol. Crystallogr*. **2009**, *65*, 148–155. https://doi.org/10.1107/S090744490804362X.
5. Farrugia, L. J. "PLATON for MS-Windows Taskbar v1.19"; University of Glasgow, **2018**.
6. Parsons, S.; Flack, H. D.; Wagner, T. "Use of Intensity Quotients and Differences in Absolute Structure Refinement". *Acta Crystallogr. Sect. B Struct. Sci. Cryst. Eng. Mater*. **2013**, *69*, 249–259. https://doi.org/10.1107/S2052519213010014.
7. Sheldrick, G. M. "SHELXT - Integrated Space-Group and Crystal-Structure Determination". *Acta Crystallogr. Sect. Found. Adv*. **2015**, 71 (Pt 1), 3–8. https://doi.org/10.1107/S2053273314026370.
8. Sheldrick, G. M. "Crystal Structure Refinement with SHELXL". *Acta Crystallogr. Sect. C Struct. Chem*. **2015**, *71*, 3–8. https://doi.org/10.1107/S2053229614024218.
9. Dolomanov, O. V.; Bourhis, L. J.; Gildea, R. J.; Howard, J. A. K.; Puschmann, H. "OLEX2: A Complete Structure Solution, Refinement and Analysis Program". *J. Appl. Crystallogr*. **2009**, *42*, 339–341. https://doi.org/10.1107/S0021889808042726.
10. Sievers, F.; Wilm, A.; Dineen, D.; Gibson, T. J.; Karplus, K.; Li, W.; Lopez, R.; McWilliam, H.; Remmert, M.; Soding, J.; Thompson, J. D.; Higgins, D. G. "Fast, scalable generation of high-quality protein multiple sequence alignments using Clustal Omega". *Mol. Syst. Biol*. **2011**, *7*, 539.
11. Kozlov, A. M.; Darriba, D.; Flouri, T.; Morel, B.; Stamatakis, A. "RAxML-NG: A fast, scalable, and user-friendly tool for maximum likelihood phylogenetic inference". *Bioinformatics* **2019**, 1-3.
12. Ronquist, F.; Teslenko, M.; van der Mark, P.; Ayres, D. L.; Darling, A.; Hohna, S.; Larget, B.; Liu, L.; Suchard, M. A.; Huelsenbeck, J. P., "MrBayes 3.2: efficient Bayesian phylogenetic inference and model choice across a large model space". *Syst. Biol*. **2012**, *61*, 539-542.
13. Cerqueira, G. C.; Arnaud, M. B.; Inglis, D. O.; Skrzypek, M. S.; Binkley, G.; Simison, M.; Miyasato, S. R.; Binkley, J.; Orvis, J.; Shah, P.; Wymore, F.; Sherlock, G.; Wortman, J. R. "The Aspergillus Genome Database: multispecies curation and incorporation of RNA-Seq data to improve structural gene annotations". *Nucleic Acids Res*. **2014**, *42* (Database issue), D705-10.
14. Edgar, R. C. "MUSCLE: multiple sequence alignment with high accuracy and high throughput". *Nucleic Acids Res*. **2004**, *32*, 1792-1797.
15. Price, M. N.; Dehal, P. S.; Arkin, A. P. "FastTree 2--approximately maximum-likelihood trees for large alignments". *PLoS One* **2010**, *5*, e9490.
16. Buchfink, B.; Xie, C.; Huson, D. H. "Fast and sensitive protein alignment using DIAMOND". *Nat. Methods* **2015**, *12*, 59-60.
17. Camacho, C.; Coulouris, G.; Avagyan, V.; Ma, N.; Papadopoulos, J.; Bealer, K.; Madden, T. L. "BLAST+: architecture and applications". *BMC Bioinformatics* **2009**, *10*, 421.
18. Kearse, M.; Moir, R.; Wilson, A.; Stones-Havas, S.; Cheung, M.; Sturrock, S.; Buxton, S.; Cooper, A.; Markowitz, S.; Duran, C.; Thierer, T.; Ashton, B.; Meintjes, P.; Drummond, A. "Geneious Basic: an integrated and extendable desktop software platform for the organization and analysis of sequence data". *Bioinformatics* **2012**, *28*, 1647-1649.
